# Clinical outcome and biomarker assessments of a multi-centre phase II trial assessing niraparib with or without dostarlimab in recurrent endometrial carcinoma

**DOI:** 10.1038/s41467-023-37084-w

**Published:** 2023-03-15

**Authors:** Ainhoa Madariaga, Swati Garg, Nairi Tchrakian, Neesha C. Dhani, Waldo Jimenez, Stephen Welch, Helen MacKay, Josee-Lyne Ethier, Lucy Gilbert, Xuan Li, Angela Rodriguez, Lucy Chan, Valerie Bowering, Blaise Clarke, Tong Zhang, Ian King, Gregory Downs, Tracy Stockley, Lisa Wang, Smitha Udagani, Amit M. Oza, Stephanie Lheureux

**Affiliations:** 1 Division of Medical Oncology and Hematology, Princess Margaret Cancer Centre, University Health Network, Toronto, ON Canada; 2 University of Toronto, Toronto, ON Canada; 3 Autonomous University of Barcelona, Barcelona, Spain; 4 Department of Medical Oncology, 12 de Octubre University Hospital, Madrid, Spain; 5 Department of Pathology and Laboratory Medicine, University Health Network, Toronto, ON Canada; 6 Division of Gynecologic Oncology, Juravinski Cancer Centre, Hamilton, ON Canada; 7 Division of Medical Oncology and Hematology, London Health Sciences Center, London Regional Cancer Program, London, ON Canada; 8 Division of Medical Oncology and Hematology, Odette Cancer Centre, Sunnybrook Health Sciences Centre, Toronto, ON Canada; 9 Division of Medical Oncology and Hematology, Kingston Health Sciences Cancer Centre, Kingston, ON Canada; 10 Division of Gynecologic Oncology, McGill University Health Centre, Royal Victoria Hospital, Montréal, QC Canada; 11 Department of Biostatistics, Princess Margaret Cancer Centre, University Health Network, Toronto, ON Canada; 12 Division of Clinical Laboratory Genetics, Laboratory Medicine Program, University Health Network, Toronto, ON Canada; 13 Department of Laboratory Medicine and Pathobiology, University of Toronto, Toronto, ON Canada

**Keywords:** Endometrial cancer, Drug development, Targeted therapies, Cancer immunotherapy

## Abstract

This multi-centre, non-randomized, open-label, phase II trial (NCT03016338), assessed niraparib monotherapy (cohort 1, C1), or niraparib and dostarlimab (cohort 2, C2) in patients with recurrent serous or endometrioid endometrial carcinoma. The primary endpoint was clinical benefit rate (CBR), with ≥5/22 overall considered of interest. Secondary outcomes were safety, objective response rate (ORR), duration of response, progression free survival and overall survival. Translational research was an exploratory outcome. Potential biomarkers were evaluated in archival tissue by immunohistochemistry and next generation sequencing panel. In C1, 25 patients were enrolled, and CBR was 20% (95% CI: 9–39) with median clinical benefit duration of 5.3 months. The ORR was 4% (95% CI: 0–20). In C2, 22 patients were enrolled, and the CBR was 31.8% (95% CI: 16–53) with median clinical benefit duration of 6.8 months. The ORR was 14% (95% CI: 3–35). No new safety signals were detected. No significant association was detected between clinical benefit and IHC markers (PTEN, p53, MMR, PD-L1), or molecular profiling (*PTEN*, *TP53*, homologous recombination repair genes). In conclusion, niraparib monotherapy did not meet the efficacy threshold. Niraparib in combination with dostarlimab showed modest activity.

## Abstract (web summary)

Treatment options in patients with recurrent endometrial carcinoma (EC) are limited and response rates to chemotherapy are poor. Here the authors report the results of a phase II trial of niraparib (PARP inhibitor) monotherapy or in combination with dostarlimab (anti-PD1) in recurrent EC.

## Introduction

Endometrial carcinoma (EC) is the gynaecologic malignancy with highest incidence and remains the fourth most common cancer diagnosis in North American women^1^. The incidence of EC is rising, mainly driven by the more aggressive non-endometrioid histologies^1,2^. Treatment options in recurrent EC are limited, and response rates to single agent chemotherapy are poor. Recent therapeutic breakthroughs have included the incorporation of immune-checkpoint inhibitors (ICI) in monotherapy in mismatch repair deficient (MMRd) patients, and in combination with targeted therapy as a non-biomarker selected strategy^3^.

A single-arm phase I trial assessing treatment with the PD-1 inhibitor dostarlimab (NCT02715284) demonstrated an objective response rate (ORR) of 42.3% (95% confidence interval [CI] 31–55%) in 104 women with MMRd recurrent or advanced EC previously treated with platinum^4^. Another cohort of the same study included 142 patients with mismatch repair proficient (MMRp) tumours, showing an ORR of 13.4% (95% CI 9.3–20.1)^5^. Single agent ICI have shown modest activity in MMRp recurrent EC^6,7^, and combination strategies may be needed to enhance the immune response and improve treatment outcomes.

A randomized phase III trial (NCT03517449) compared pembrolizumab and lenvatinib to single agent chemotherapy in patients with EC previously treated with platinum^8^. The study showed an increase in progression free survival (PFS; 7.2 vs 3.8 months; HR 0.56 [95% CI 0.47–0.66]) and overall survival (OS; 18.3 vs 11.4 months; HR = 0.62 [95% CI: 0.51–0.75]), favouring the pembrolizumab and lenvatinib arm^8^. Yet, the combination was associated with 89% grade ≥3 adverse events, that may require proactive medical management and patient monitoring. Cabozantinib as a single agent has shown a signal of activity in recurrent endometrioid (ORR 14%, PFS 4.8 months) and serous (ORR 12% and PFS 4.0 months) EC in a phase II trial^9^, which may be enhanced when administered in combination with nivolumab (ORR 25%; PFS 5.3 months)^10^.

Other potential combination therapies with ICI in EC include DNA damaging agents. Preclinical studies have shown synergy between combining a PARP inhibitor and ICI^11,12^. Combination of these agents may enhance the immunogenic cell death, alter the tumour microenvironment and/or stimulate neoantigen production, activating an antitumour immune response^12^. In terms of subgroups of patients that may benefit from DNA damaging agents, several potential biomarkers have been proposed. Endometrioid EC often show alterations in *PTEN* (up to 78%)^13^. Loss of PTEN function can cause defects in repair of DNA double-strand breaks by homologous recombination, and in preclinical studies PTEN loss has been described as a possible biomarker of response to PARP inhibitors^14,15^. In non-endometrioid histologies, homologous recombination deficiency (HRd), a biomarker of response to PARPi in ovarian cancer, has been associated with some tumours harbouring *TP53* mutations^16^.

Defining the molecular vulnerabilities of recurrent EC may guide treatment strategy. Blood based biomarkers have shown the potential of capturing multiclonal heterogeneity over time in certain tumour sites. To our knowledge, the potential of ctDNA to monitor the tumour evolution and as a biomarker for treatment selection has not yet been described in EC.

In this work we assess whether the PARP inhibition approach with niraparib, or the combination of niraparib and dostarlimab, provides clinical benefit in patients with recurrent EC. Exploratory analyses include immunohistochemistry (IHC), genomic and ctDNA-based biomarker analysis, and association between a ctDNA-based genomic panel with tissue profiling^17^.

## Results

**Fig. 1:**
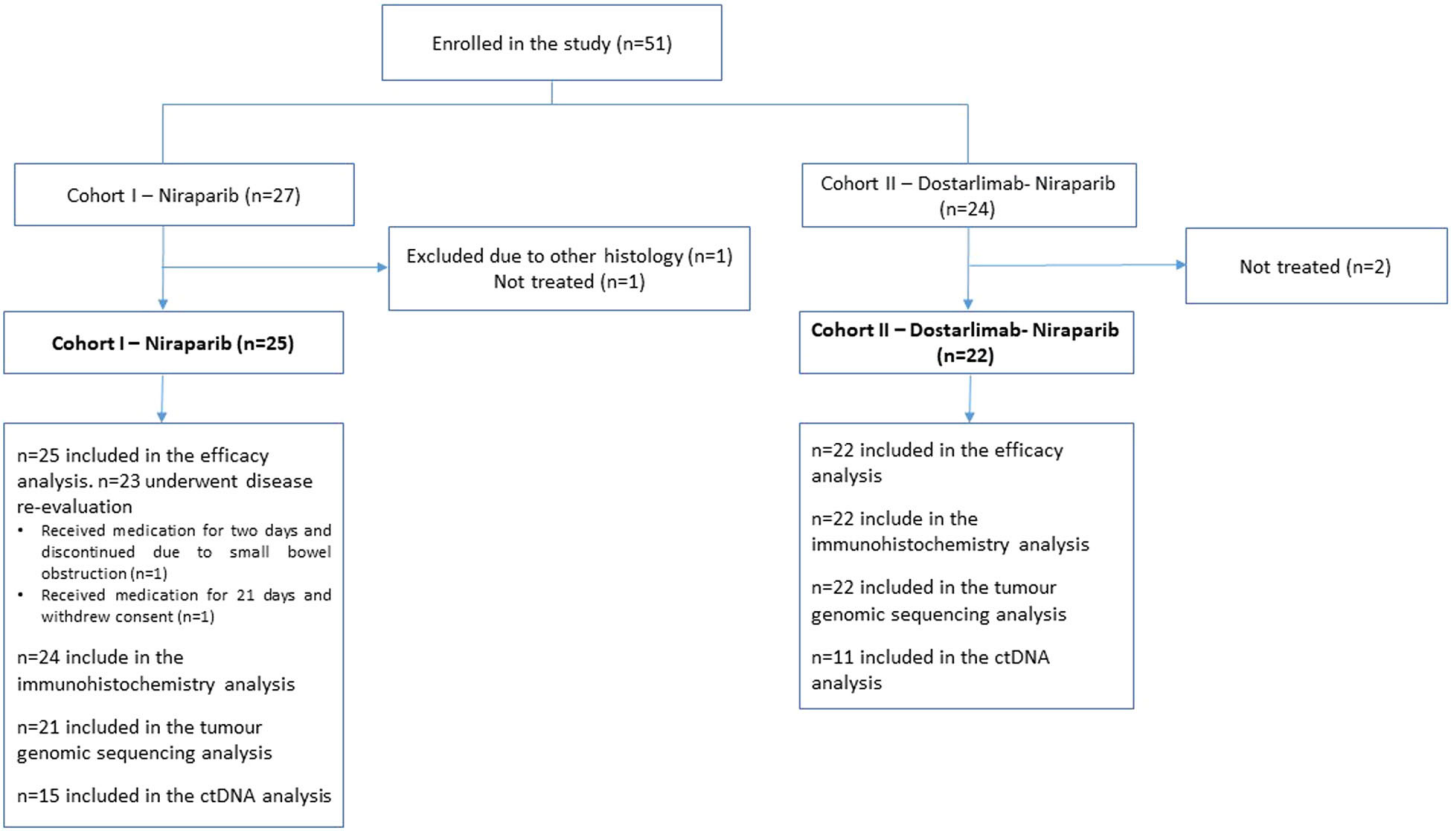
CONSORT flow diagram of patients enrolled in the study. Results are shown per cohort, including the number of patients evaluated for efficacy and translational studies.

Forty-seven patients with recurrent EC were treated between November 2017 and January 2021 (data cut-off) in six Canadian centres (Fig. 1). Two patients in cohort 1 (C1), assessing niraparib, started therapy but were not evaluated for treatment efficacy due to development of malignant bowel obstruction on day 2 of therapy (*n* = 1) and withdrawal of consent during the first cycle (*n* = 1). At data cut-off two patients in cohort 2 (C2), assessing niraparib and dostarlimab, continued treatment. The baseline demographic characteristics of patients are shown in Table 1.

**Table 1:** Baseline patient characteristics

		C1 – Niraparib (*n* = 25)	C2 – Niraparib + Dostarlimab (*n* = 22)
Age	Median (range)	69 (53–80)	64.5 (38–80)
ECOG status	0	5 (20%)	2 (9%)
	1	19 (76%)	18 (82%)
	2	1 (4%)	2 (9%)
Histology	Serous	15 (60%)	9 (41%)
	Endometrioid grade 1	3 (12%)	3 (14%)
	Endometrioid grade 2	3 (12%)	2 (9%)
	Endometrioid grade 3	3 (12%)	7 (32%)
	Mixed serous and endometrioid	1 (4%)	1 (5%)
Molecular characteristics	MMR deficient	4 (16%)	3 (14%)
	p53 abnormal or overexpressed	15 (60%)	12 (55%)
	POLE mutant	0	1 (5%)
Prior Regimens	1	8 (32%)	6 (27%)
	2	9 (36%)	6 (27%)
	3	2 (8%)	5 (23%)
	4	6 (24%)	3 (14%)
	5	0	1 (5%)
	6	0	1 (5%)
Number of prior regimens	Median (range)	2 (1–4)	2 (1–6)
Prior Therapy^a^	Systemic platinum chemotherapy	25 (100%)	22 (100%)
	Radiation	18 (72%)	19 (86%)
	Surgery	21 (84%)	22 (100%)
Platinum sensitivity^b^	Platinum Resistant	19 (76%)	15 (68%)
	Platinum Sensitive	6 (24%)	7 (32%)

^a^None of the patients received prior immune-checkpoint inhibitor therapy.

^b^Platinum sensitivity was defined as per the definition utilized in ovarian cancer, based on platinum free interval time. Platinum sensitive: disease relapse occurs >6 months from last dose of platinum chemotherapy; Platinum resistant: Disease relapse occurs <6 months from last dose of platinum chemotherapy.

### Cohort 1: niraparib monotherapy

Twenty-five patients were enrolled (Fig 1). Median age was 69 years, and 64% of patients had serous EC, being 76% of tumours platinum resistant (Table 1). The median prior lines of therapies was two (range 1–4), including chemotherapy (all patients), hormonal therapy (4 patients), and targeted therapy (2 patients).

Median number of cycles of niraparib was three (1–8). The clinical benefit rate (CBR) was 20% (5/25; 95% CI: 9–39), with a median clinical benefit duration of 5.3 months (range 1.8–7.2). The ORR was 4% (1/25; 95% confidence interval [CI] 0–20), with one patient with serous EC experiencing a partial response (Fig. 1). Considering the platinum free interval (cut-off of 6 months), the ORR was 16.7% (1/6) and 0% in platinum sensitive and resistant disease, respectively. The median PFS was 2.5 months (95% CI 1.8–3.7), and median OS was 12.5 months (95% CI 6.6–19.3) (Supplementary Fig. 1).

Adverse events that were considered to be related to therapy were mostly grade 1–2. Related grade ≥3 adverse events (AEs) occurring in ≥ 10% of patients were anaemia (24%), fatigue (16%) and thrombocytopenia (16%). Any AE occurring in ≥15% of patients is shown on Table 2. There were no grade 5 adverse events. Discontinuations due to AEs occurred in four patients (16%), and reason for discontinuation were fatigue (*n* = 2), bowel obstruction (*n* = 1) and other not specified (*n* = 1). Dose reductions of niraparib occurred in 36% of patients (8/25; one patient had three AEs as cause of dose reduction), due to haematologic toxicity (*n* = 6), followed by fatigue (*n* = 2) and/or gastrointestinal AEs (*n* = 2).

**Table 2:** Adverse events occurring in ≥15% of patients in any treatment group

	C1 – Niraparib (*n* = 25)		C2- Niraparib + Dostarlimab (*n* = 22)	
AE detail	Grade ≥ 3	Total	Grade ≥ 3	Total
Nausea	0 (0%)	14 (56%)	1 (5%)	13 (59%)
Fatigue	5 (20%)	15 (60%)	1 (5%)	11 (50%)
Dyspnoea	0 (0%)	11 (44%)	2 (9%)	13 (59%)
Anaemia	7 (28%)	12 (48%)	6 (27%)	9 (41%)
Constipation	0 (0%)	11 (44%)	0 (0%)	5 (23%)
Dizziness	0 (0%)	6 (24%)	0 (0%)	9 (41%)
Vomiting	0 (0%)	8 (32%)	0 (0%)	8 (36%)
Creatinine increased	0 (0%)	6 (24%)	0 (0%)	7 (32%)
Anorexia	0 (0%)	7 (28%)	0 (0%)	5 (23%)
Cough	0 (0%)	4 (16%)	0 (0%)	7 (32%)
Palpitations	0 (0%)	4 (16%)	0 (0%)	7 (32%)
Platelet count decrease	4 (16%)	5 (20%)	2 (9%)	6 (27%)
Diarrhoea	0 (0%)	4 (16%)	0 (0%)	5 (23%)
Abdominal pain	1 (4%)	5 (20%)	0 (0%)	4 (18%)
Insomnia	0 (0%)	5 (20%)	0 (0%)	4 (18%)
Gastroesophageal reflux	0 (0%)	2 (8%)	0 (0%)	6 (27%)
Headache	0 (0%)	6 (24%)	0 (0%)	2 (9%)
Hypertension	2 (8%)	6 (24%)	1 (5%)	2 (9%)
Neutrophil count decreased	1 (4%)	3 (12%)	3 (14%)	4 (18%)
Hyponatremia	2 (8%)	6 (24%)	0 (0%)	1 (5%)
Back pain	0 (0%)	5 (20%)	0 (0%)	2 (9%)
Bloating	0 (0%)	1 (4%)	0 (0%)	5 (23%)
White blood cell decreased	0 (0%)	4 (16%)	1 (5%)	2 (9%)
Myalgia	0 (0%)	0 (0%)	1 (5%)	5 (23%)
Hypomagnesemia	0 (0%)	4 (16%)	0 (0%)	1 (5%)

Data are represented in *n* (%). The order of the adverse events follows the total frequency in both cohorts. Generalized muscle weakness and general muscle weakness have been merged.

### Cohort 2: niraparib and dostarlimab

Twenty-two patients were enrolled in C2 (Fig 1). Median age was 64 years, 46% had a serous histology and 68% had a platinum-resistant tumour. The median prior lines of therapies was two (range 1–6), including chemotherapy (all patients), hormonal therapy (4 patients) and targeted therapy (2 patients). Three patients had MMR deficient (MMRd) tumours (14%).

**Fig. 2:**
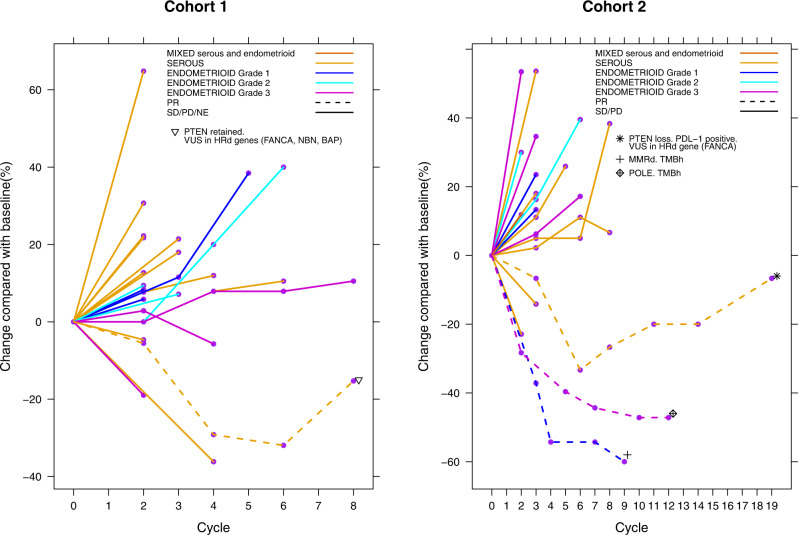
Spider plot showing the best response as per RECIST 1.1 criteria and duration of response as per histological subtype. Cohort 1 with niraparib monotherapy (*n* = 23), and Cohort 2 with niraparib and dostarlimab (*n* = 22).

Median number of cycles was three (range 1–20). The CBR was 31.8% (7/22; 95% CI 16–53) and median clinical benefit duration was 6.8 months (95% CI 3.7–9.5). The ORR was 14% (3/22; 95% CI 3–35), with three patients experiencing a partial response (Fig. 2). Out of the three responders, one had a MMRd tumour, and one harboured a somatic *POLE* mutation. Taking into account the platinum free interval, the ORR was 14.3% (1/7) and 13.3% (2/15) in platinum sensitive and resistant disease, respectively. The ORR was 33.3% (1/3) in MMRd and 10.5% (2/19) in MMRp patients. The median PFS was 2.4 months (95% CI: 1.6–3.7), and median OS was not reached (95% CI: 5.7—not reached).

Adverse events that were considered related to therapy were mostly grade 1–2. Related grade ≥3 AEs occurring in ≥ 10% of women were anaemia (27%) and neutropenia (14%). One patient experienced an AE of special interest, grade 3 myasthenia gravis. Any AE occurring in ≥15% of women is shown on Table 2. There were no grade 5 adverse events. Discontinuation due to AEs occurred in one patient (4.5%); reason for discontinuation was myasthenia gravis (*n* = 1). Dose reductions of niraparib occurred in 45% of patients (10/22), due to haematological AEs (*n* = 5), fatigue (*n* = 2), diarrhea (*n* = 1), palpitations (*n* = 1), hypertension (*n* = 1).

### Correlative studies

Correlative analyses were performed on archival tissue. Forty-six patients had sufficient tissue available and were included in the immunohistochemistry analysis (24/25 from C1 and all from C2), and forty-three in the molecular analysis (21/25 from C1 and all from C2; Fig. 1).

An overview of the immunohistochemistry and genomic findings per cohort and histology are listed in supplementary table 1. PD-L1 positivity (1% combined positive score [CPS] cut-off) was seen in 40% and 64% of samples in C1 and C2, respectively. MMR deficiency was detected in 16% and 14% of samples in C1 and C2, respectively.

PTEN loss by IHC was present in 32% and 50% of samples in C1 and C2, respectively. Based on the molecular profiling results, 33.3% (9.5% serous and 24% endometrioid) of C1, whereas 41% (35.5% endometrioid and 4.5% mixed serous and endometrioid) of cases of C2 harboured *PTEN* alterations by next generation sequencing (NGS). The presence of PTEN alterations by IHC had a sensitivity of 80% and a specificity of 75% in predicting a *PTEN* oncogenic mutation.

Abnormal p53 by IHC was seen in 56% and 55% of patients in C1 and C2, respectively. Alterations in *TP53* by NGS were detected in 76% (57% serous, 14% endometrioid and 5% mixed) of patients in C1, and 54.5% (41% serous, 9% endometrioid and 4.5% mixed) in C2. All tumours that were p53 abnormal on IHC testing also had a *TP53* genomic alteration.

**Fig. 3:**
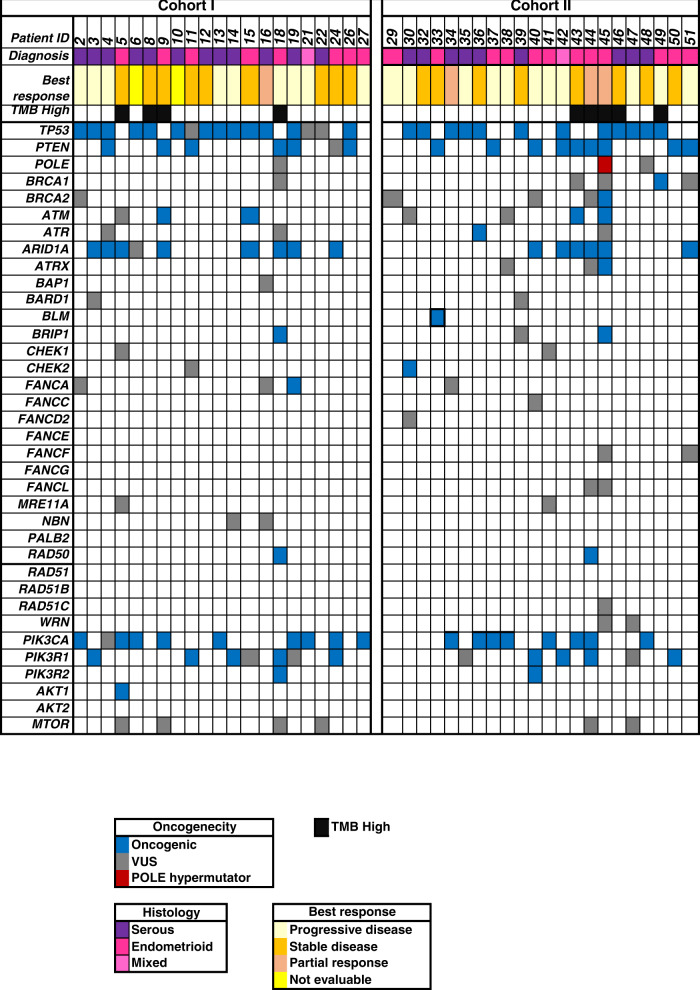
Oncoprint representing distribution of oncogenic and variants of uncertain significance (VUS) from archival tumour tissue. Cohort 1 with niraparib monotherapy (*n* = 21). Cohort 2 with niraparib and dostarlimab (*n* = 22).

Oncogenic alterations in homologous recombination repair (HRR) genes were seen in 38% and 45.4% of patients in C1 and C2, respectively, with *BRCA1/2* oncogenic variants detected in 9% in C2 and none in C1. No *BRCA1/2* reversion variants were detected. An oncogenic *POLE* variant was present in one patient in C2. No *CCNE1* amplifications were detected. Oncogenic alterations in the *PI3K* pathway genes (namely *PIK3CA, PIK3R1, PIK3R2, ATK1, AKT2* and *MTOR*) were detected in 62% and 50% of patients in C1 and C2, respectively (Fig. 3). A tumour mutation burden (TMB) score of >20% was considered high. The TMB-high cases were distributed in C1 and C2 at 19% and 23% respectively, and half of them were MMRd tumours (Fig 3).

No significant association was detected between clinical benefit and IHC markers (PTEN, p53, MMR, PDL-1), or NGS (*PTEN*, *TP53*, HRR genes, TMB-high) in C1 and C2. Similarly, none of the biomarkers had a statistically significant association with longer PFS. In C2, the median PFS was 3.6 months (95% CI 1.6-not reached) in those with PTEN loss vs 1.8 months (95% CI 0.5–3.6) in PTEN retained (*p* = 0.07). The median PFS in TMB-high was 7.4 months (95% CI 1.1-not reached) vs not high TMB 1.8 months (95% CI 1.6–3.6; *p* = 0.06).

**Fig. 4:**
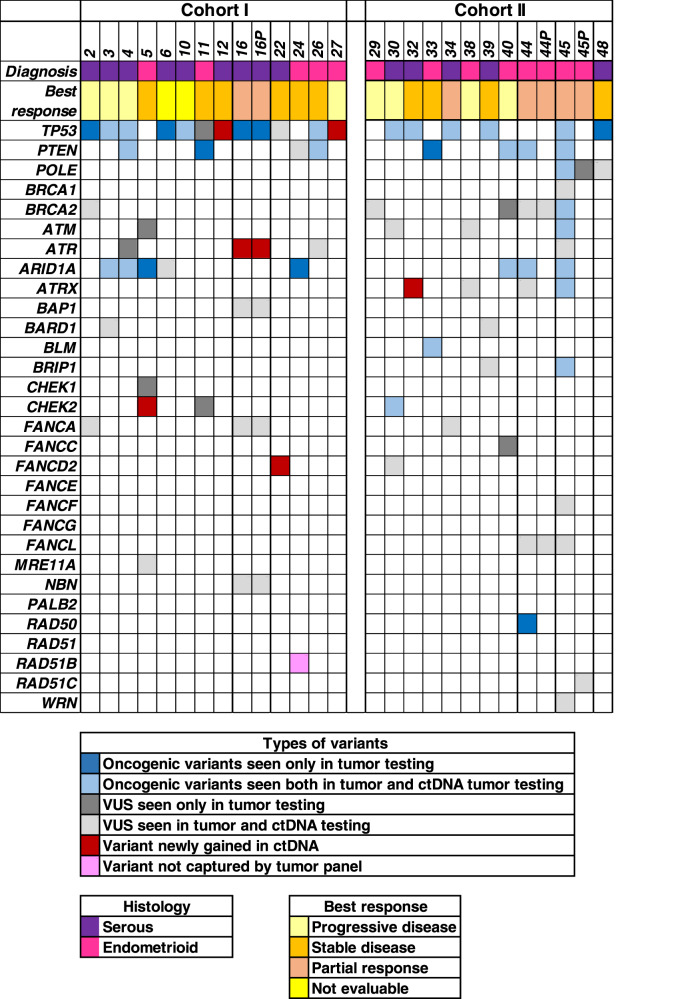
Oncoprint representing distribution of oncogenic and variants of uncertain significance (VUS) in ctDNA at baseline. Cohort 1 includes patient identifications listed as NEC-2–27 (*n* = 13), and Cohort 2 includes patient identifications listed as NEC-29–48 (*n* = 11). p: second sample.

We tested the feasibility of assessing HRR in the baseline ctDNA samples from EC using a custom NGS panel. Baseline blood sample for ctDNA analysis was available in 26 patients (C1 *n* = 15, C2 *n* = 11) and 24 of them had a matching tumour sample. Median time from tumour to blood sample collection was 2.4 years (range 0.32–8.3). Variants in the ctDNA panel were detected in 92% (24/26) of patients (Fig. 4). The detection of oncogenic *TP53, PTEN* or HRR gene variants between tumour and ctDNA was significantly associated (*p* < 0.01). Interestingly, additional variants were detected in 25% (6/24) of patients that were undetected in previous tumour testing (Fig. 4); however, 21% (5/24) of them were VUS (Supplementary Table 2). There was no association between presence of HRR oncogenic variants in the ctDNA and clinical benefit or PFS.

In four patients who had a long response to treatment (PFS > 6 months), ctDNA was collected at a second time point. The two patients from C1 (NEC11—grade 3 endometrioid carcinoma and NEC16—serous EC; Figs. 3 and 4) had ctDNA collected at the time of progression. In NEC-011, no variants were detected in both ctDNA samples (Supplementary Table 2). In patient NEC16 an *ATR* VUS (c.6793G>A; p.Val2265Ile) was detected with increasing variant allele frequency in samples collected prior to start of treatment and upon progression (VAF 1.4% vs 3.4%). There were other variants detected in HRR genes in both the samples for this patient (Supplementary Table 3), corresponding to likely germline variants as observed at VAF close to 50%. The two patients from C2 (NEC44—MMRd grade 1 endometrioid and NEC45—grade 3 endometrioid with a *POLE* variant; Figs. 3 and 4) had a second time point of ctDNA collected while on maintained response to therapy. In both cases, the variants that were seen below VAF of 20% seen in pre-treatment samples were not detected in the ctDNA sample collected while the patients were still responding to treatment. The only variants seen in the samples collected at response to therapy were the likely germline variants as observed at VAF close to 50% (Supplementary Table 3). No reversion *BRCA1/2* variants were detected in ctDNA samples.

## Discussion

In this pilot phase II trial, patients with recurrent EC were enrolled in two consecutive cohorts. In C1, niraparib as a single agent did not meet the pre-specified efficacy criteria. The CBR and ORR observed in C2 with the combination of niraparib and dostarlimab were aligned with other studies that assessed the role of ICI monotherapy in non molecularly selected EC, suggesting no synergistic activity as per the data in this study^5^. One of the limitations of the study is its heterogeneous population, in terms of histological, molecular characteristics and platinum sensitivity. In this study, a predominantly platinum-resistant population was included^18^. While platinum sensitivity is a known biomarker of response to PARP inhibition in ovarian carcinoma^18^, its role in EC is not established and the platinum free interval is not clearly defined to guide treatment strategy in clinic. In the trial, only one partial response was observed in the niraparib monotherapy cohort (C1), corresponding to a patient with platinum sensitive disease, while no response was observed in the platinum resistant. In the combination cohort (C2), partial response was observed in two patients with platinum-resistant disease and biomarkers of response to ICI (MMRd or POLE mutation, both with TMB-high), and one patient with platinum sensitive disease with no clear biomarkers of response to ICI. While numbers are too small to draw any conclusions, given the relation between PARPi and platinum sensitivity^18,19^, assessing the role of PARP inhibition earlier in the EC diagnosis or prior to platinum resistance may be interesting.

PARP inhibitor maintenance therapy has changed the treatment landscape of high-grade serous ovarian carcinoma (HGSOC)^19^. The cancer genome atlas described that HGSOC and serous EC, have pathologic and molecular similarities^13^. Response to PARP inhibition in HGSOC has been determined by molecular subgroups with the presence of *BRCA1/2* mutations suggested best activity, followed by HRd, and at a lesser extent in the non-HRd subgroup^19^. The association between the presence of *BRCA1/2* mutations and response to PARP inhibition in EC is unclear, although anecdotal single patient responses have been reported^20,21^. A profiling study (NGS600 testing) showed that the frequency of alterations in HRR related genes was high in EC, compared to other cancer types, accounting for 34.4%^22^. The most frequently altered genes were *ARID1A* (27%), *ATM* (4.61%), *ATRX* (3.13%) and *BRCA2* (3.05%)^22^. Results according to histological subtype were not reported. De Jonge et al. assessed the functional HRd in EC using a RAD51 assay^16^. The study showed that 24% of all EC were HRd, which was only restricted to non-endometrioid histologies (46% of non-endometrioid carcinomas classified as HRd)^16^. In the current study, oncogenic alterations in HRR genes were detected in 24% of serous and 60% of endometrioid carcinomas, respectively (Supplementary Table 1). Amongst these alterations, oncogenic variants in *ARID1A* contributed largely. Therefore, data were reassessed after removal of *ARID1A* from the HRR gene list. Following exclusion of *ARID1A* oncogenic variants, oncogenic HRR gene variants were detected in 14.3% (3/21) of serous and 38% (8/21) of endometrioid carcinomas. No association was detected in this study between HRR gene status and clinical benefit with or without oncogenic *ARID1A* alterations. The role of alterations in HRR genes as a biomarker of response is not established in EC.

The optimal way of defining and evaluating HRd, both genotypically and phenotypically, is not well established. In HGSOC, companion diagnostics can identify patients with a ‘genomic scar’ that reflects an underlying genomic instability or HRd phenotype, which is considered a biomarker of response to PARP inhibition^23^. However, the HRd phenotype is dynamic over time and with treatment pressure, not reflecting potential acquired resistance mechanisms^23^. The definition of HRd genotype, beyond *BRCA1/2* variant, as biomarker of response to PARP inhibition is under investigation. Small studies have suggested the role of *RAD51C* variants and promoter methylation as a biomarker of better outcomes with PARP inhibition in HGSOC^23–25^. However, studies assessing the predictive role of non-*BRCA* HRR mutations have been inadequately powered to draw conclusions, and HRR gene selection is not well established. In the current study, the HRd phenotype through companion diagnostics was not measured, given that the study population was platinum resistant enriched and archival tissue was employed, which would have limited the interpretations of the ‘genomic scarring’ results. The HRR gene selection was performed based on previously defined most frequent HRR mutations across multiple tumours^22^.

*PTEN* variant is the most common molecular-genetic event in endometrioid EC, and is rarely seen in serous subtype^13^. PTEN IHC is not widely used in routine clinical practice, in part owing to ill-defined staining interpretation criteria^26^. Although there is good agreement between PTEN IHC and *PTEN* loss of function mutation, it is not considered a surrogate^27^. In this study, a complementary interpretation algorithm has been implemented^27^, whereby PTEN status is designated abnormal if detected by IHC, NGS, or both. Based on preclinical data in EC cell lines, we anticipated that tumours with alterations in PTEN, would be more likely to respond to PARP inhibition^14,15^. PTEN protein has an important role in maintaining the genomic integrity, as it upregulates the RAD51 expression levels^14,15^. It has also been proposed that PTEN loss may mediate resistance to ICI through activation of the PI3K pathway^28^. In the trial we detected PTEN loss in 29%, 45% and 60% of serous, low and high-grade endometrioid carcinomas, respectively. No association with clinical benefit were detected according to PTEN status (genomic, protein loss, or combination) in C1 or C2. There were differences in PFS in patients according to PTEN status by IHC (PTEN lost median 3.6 months [95% CI 1.6-not reached] vs PTEN retained 1.8 months [95% CI 0.5–3.6]; *p* = 0.07) in C2, which did not reach statistical significance.

The selection of patients for anti-PD-1/PD-L1 therapy may be guided by PD-L1 IHC assays. Scoring cut-offs vary according to tumour type and individual ICI agents. In EC, several studies have reported PD-L1 expression in tumour cells and tumour-associated inflammatory cells^28^. In an exploratory analysis of a phase II trial assessing durvalumab in recurrent EC, the presence of tumour-associated immune cells correlated better with outcomes than PD-L1 staining of tumour cells and immune cells^29^. In our study, no association with clinical outcomes was detected according to PD-L1 CPS status. Another biomarker that has been proposed to predict response to ICI includes the TMB^30^. Treatment with pembrolizumab as monotherapy was granted approval from the Food and Drug Administration for solid tumours with ≥10 mutations per megabase that had progressed to prior line of therapy^30^. The cut-off used to define TMB-high in this study was the top 20% mutation load within EC patients assessed, following the approach described in Samstein et al.^31^. In C2 numbers were too small to establish an association between TMB-high and response (PFS in TMB-high 7.4 months [95% CI 1.1-not reached] vs not high TMB 1.8 months [95% CI 1.6–3.6]; *p* = 0.06).

The combination of ICI and the PARP inhibitor talazoparib showed an ORR of 11.4% in a small phase II trial in MMRp recurrent EC^32^. Other combinations that have been assessed with both PARP inhibition and ICI include antiangiogenics. In this setting, a randomized phase III trial assessing pembrolizumab and lenvatinib has demonstrated improved PFS and OS in advanced EC following prior therapy, when compared to single agent chemotherapy^8^. The combination of antiangiogenics with PARP inhibition has also been assessed in a phase II trial (NCT03660826)^33^. In this three-arm randomized trial, PFS was 3.8 months for cediranib alone, 2 months for olaparib and 5.5 months for olaparib and cediranib combination^33^. However, the between-arm differences were not statistically significant. The role of triplet therapy with antiangiogenics, immune-checkpoint therapy and PARP inhibition has not yet been reported. A phase I/II study showed promising activity of the PARP inhibitor olaparib in combination with metronomic cyclophosphamide and metformin in recurrent or metastatic EC^34^. In fact, metformin may have a synergistic activity with PARP inhibition, via direct (insulin-independent) and indirect effects, through the PIK3CA-AKT-mTOR pathway^35^. Targeting the cell cycle modulation and replication stress has also a special interest in EC, particularly in the serous subtype^3^. In this setting, a small non-randomized phase II study assessing Wee1 inhibition in monotherapy with adavosertib in serous EC, showed promising clinical activity, with an ORR of 29.4% and 6-month PFS of 47.1%^3,36^.

Circulating tumour DNA (ctDNA) is increasingly becoming important for disease monitoring as the tumour evolves, and potentially guiding which patients may experience a benefit from treatment. In ovarian cancer presence of *BRCA* reversion mutations in ctDNA, is a known marker of absence of benefit from the PARP inhibitor^17^. Disease evolution overtime also plays a critical role in EC, as newly acquired MMRd has been described in the recurrent setting^37^. One study suggests that ctDNA might be used as a tool for early detection and monitoring disease recurrence in EC^38^. In this study, we aimed to test the feasibility and clinical utility of monitoring HRR gene status in the ctDNA samples of EC and guiding response towards niraparib using a targeted sequencing customized panel. Even though the median time from archival sample retrieval to ctDNA sample was 2.4 years, the results indicated a high degree of concordance in the detection of oncogenic *TP53*, *PTEN* and HRR gene variants between tumour and ctDNA. Further evaluation of the peripheral blood PBMCs would help exclude contribution from mutations arising from age related clonal hematopoiesis^39^. There was no significant association between HRR gene status in ctDNA and clinical outcome, However, our results indicate that ctDNA analysis may be feasible for biomarker selection in clinical trials (i.e. oncogenic *ARID1A* detected in 20% of blood samples), as suggested by the significant association of archival tumour mutations and ctDNA.

The role of PARP inhibition and ICI is currently being assessed earlier in the therapeutic armamentarium of EC, with several ongoing studies assessing these agents along with chemotherapy in the front-line setting, prior to the development of resistance to platinum. Ongoing studies include chemotherapy with maintenance PARP inhibition (CAN-STAMP NCT04159155, RAINBO), ICI (NCT03981796, NCT03914612,NCT04269200,NCT03603184), and both strategies (NCT03981796, NCT04269200). It will be important to determine the therapeutic selection at each time point, including the role of early administration of PARP inhibition and/or ICI therapy, and potential biomarker selection.

## Methods

A multi-centre, open-label, two-stage, phase II study assessed niraparib monotherapy or in combination with dostarlimab in recurrent EC (NCT03016338). The study initially enrolled patients with recurrent EC to the niraparib monotherapy cohort (cohort 1—C1). Once C1 was completed, a sequential second cohort assessed niraparib in combination with dostarlimab (cohort 2—C2). Cross-over between cohorts was not permitted. The trial complied with all relevant ethical regulators. The protocol was approved by the Ontario Cancer, McGill University, Alberta Health Research Ethics Board, and Health Canada. All patients provided written informed consent. The study design and conduct complied with all relevant regulations regarding the use of human study participants and was conducted in accordance with the criteria set by the Declaration of Helsinki. There was no compensation for study participants. Enrollment occurred between the 17 November 2017 and 29 January 2019 in Cohort 1, and 2 October 2019 and 8 October 2020 in Cohort 2.

Patients with recurrent serous or endometrioid EC were enrolled. There was no limit on prior lines of therapy, and prior platinum-based chemotherapy was required with no limitation on timing. Previous treatment with a PARP inhibitor, or other targeted therapy directed against the homologous recombination pathway was not allowed. Enrolled patients had an Eastern Cooperative Group (ECOG) performance status of ≤2. Within 7 days of the proposed start of treatment, patients had adequate organ and marrow function (protocol in supplementary note 1). In cohort 2, prior ICI was not allowed, and participants receiving corticosteroids were eligible if the dose was stable for at least four weeks prior to initiating protocol therapy. Refer to protocol for full eligility criteria (Supplementary note 1). Mandatory archival tissue was requested for molecular profiling and blood samples were collected for ctDNA at baseline for patients (correlative studies performed as part of NCT03420118, NCT03702309 and NCT02906943 studies).

In the first cohort patients received niraparib 200 or 300 mg orally once daily, based on baseline body weight and platelet count, in a four-week cycle. In the second cohort niraparib (same dose and schedule) was given with dostarlimab 500 mg intravenously every three weeks for four cycles, followed by 1,000 mg every six weeks thereafter.

The primary endpoint of the trial was clinical benefit rate (CBR) in the intention-to-treat population, which includes complete or partial response, or stable disease ≥16 weeks. Secondary endpoints included ORR, PFS, OS, and safety and tolerability assessment. Response assessment was performed per RECIST (Response Evaluation Criteria in Solid Tumours) v1.1 every eight weeks. All patients who initiated treatment were evaluable for safety and toxicity from first treatment dose. Adverse event (AE) grading was per the Common Terminology Criteria for Adverse Events (CTCAE) v4.0. Exploratory objectives included assessment of PTEN, MMR status and PD-L1 by IHC as a predictor of response to therapy, as well as the role of genes involved in the HRR pathway, *CCNE1* amplifications and alterations in *PTEN* by NGS as biomarkers of outcome.

### Correlative studies

Formalin fixed, paraffin-embedded (FFPE) sections of archival tumour tissue were used. Haematoxylin & eosin (H&E) and immunohistochemistry (IHC) stains were examined (Supplementary Fig. 2). The stains were performed on 4μm whole sections of FFPE tissue, which were processed using standard techniques. A single H&E stain was undertaken to assess routine histological features. The IHC panel comprised PD-L1, p53, PTEN, and mismatch repair (MMR) proteins MLH1, PMS2, MSH2 and MSH6. IHC staining was undertaken according to the manufacturer’s instructions using the following antibodies: PD-L1 (Agilent Technologies, clone 22C3 pharmDx, 1:100), p53 (Leica, clone D0-7, 1:1000), PTEN (Cell Signaling, clone 138G6, 1:50), MLH1 (DAKO, clone ESOS, pre-dilute), PMS2 (BD Pharmigen, clone 556415, 1:200), MSH2 (BD Pharmigen, clone 556349, 1:500) and MSH6 (Abcam, clone ab92471, 1:150).

The H&E- and IHC-stained slides were assessed by a gynaecology expert pathologist blinded to clinical data. A second pathologist examined equivocal cases to reach consensus. PD-L1 expression was defined as complete or partial membrane staining in tumour cells (TC) and membranous and/or cytoplasmic staining in immune cells (IC) – namely, lymphocytes and macrophages. We determined the percentage of positive TCs and ICs in combination, using the combined positive score (CPS). CPS was derived by dividing the total number of PD-L1 positive cells (TCs and ICs) by the number of viable TCs and multiplying by 100. The cut-off value for positive PD-L1 staining was set at 1%. Normal tonsil was used as positive control. For p53, strong positive nuclear expression in >80% of TCs (overexpression pattern) and complete loss of expression in TCs with a positive non-tumour internal control (null pattern) were considered mutation-type. Wild-type (normal) expression was defined as heterogeneous weak to moderate staining. PTEN was scored as either retained (staining of similar intensity seen in TCs relative to non-tumour internal control) or complete absence (negative PTEN staining in TCs with retained expression in non-tumour internal control). MMR protein status was considered deficient (MMRd) when the tumour showed complete loss of nuclear expression in any MMR protein (MLH1, PMS2, MSH2, MSH6). Stromal cells, inflammatory cells and non-tumour epithelial cells served as internal control for MMR, similar to p53 and PTEN.

Tumour genomic profiling was conducted as part of two correlative studies (NCT03420118, NCT02906943). A multigene targeted panel spanning exonic regions of 555 cancer-related genes (UHN Hi5 Panel) at the College of American Pathologists/Clinical Laboratory Improvement Amendment (CAP/CLIA)-accredited Advanced Molecular Diagnostics Laboratory (AMDL) at Princess Margaret Cancer Centre^40^. Besides, *TP53, PTEN* we reviewed mutations in HRR pathway, *ARID1A, ATM, ATR, BAP1, BARD1, BLM, BRIP1, CHEK1/2, FANCA/C/D2/E/F/G/L, MRE11A, NBN, PALB2, POLE, RAD50, RAD51, RAD51B*, and *WRN*^20^. In addition, we reviewed mutations in genes involved in the PI3Kinase pathway- mainly, *PIK3CA, PIK3R1, PIK3R2, MTOR* and *AKT1/2*. For *CCNE1* amplifications, copy number variants were examined in NGS data using two callers-CNVkit (version 0.7.11) and Contra (version 2.0.8)^41^. A fold change of ≥2.5 observed by both pipeline callers was considered a *CCNE1* amplification. Tumour mutational burden (TMB) was calculated as mutations per megabase, counting variants in coding regions with a depth greater than 50, and a variant allele frequency greater than 8%, while excluding driver mutations (COSMIC), technical artifacts, and variants with minor allele frequency greater than 0.001 in the gnomAD database. TMB-high was defined as falling within the top 20% mutation burden of all historic endometrial cancers.

The ctDNA analysis was performed as part of LIBERATE (NCT03702309) study. Extraction of ctDNA was performed from baseline plasma samples and analyzed using a custom designed panel. Exonic coding regions and ±20 bp of the intron for the following genes (*ARID1A, ATM, ATR, ATRX, BAP1, BARD1, BLM, BRCA1, BRCA2, BRIP1, CHEK1, CHEK2, CCNE1, FANCA, FANCC, FANCD2, FANCE, FANCF, FANCG, FANCL, MRE11A, NBN, PALB2, POLE, PTEN, RAD50, RAD51, RAD51B, RAD51C, RAD51D, TP53* and *WRN*) were examined using SureSelect Target Enrichment hybrid capture followed by paired-end sequencing (Illumina, California, USA). Variant calls are generated using the UHN AMDL custom bioinformatics pipeline with alignment to genome build GRCh37/hg19, and variants assessed using Alissa Interpret (Agilent, California, USA). The reportable range was 1–100% variant allele frequency, and test sensitivity >94% for detection of substitutions and small insertions/ deletions (≤25 bp).

### Statistics and trial design

The trial was designed as a multicenter, non-randomized, open-label, phase II study. A Simon two-stage design was employed, with the null hypothesis that CBR, *p* ≤ 0.10 versus the alternative that p ≥ 0.35 and setting alpha = beta = 0.10. In C1 stage I, the accrual of 10 patients was planned. If at least one clinical benefit instance was observed at the end of stage I, the study would proceed to stage II with 12 additional patients to be accrued (total 22 evaluable patients). If at least five instances of clinical benefit were observed among the 22 patients, this agent would be considered worthy of further investigation. If the CBR does not reach the pre-defined level (positive ≥5/22 overall) after stage II in C1, PTEN analysis will be performed, and the study may be considered to expand to PTEN-loss subgroup. After the enrollment in C1 (niraparib alone) is completed, new patients were registered in C2 with the combination of niraparib and dostarlimab. In C2, the same criteria (≥1/10 CBR to proceed to stage II, and positive study ≥5/22 CBR overall) was used.

Patient demographics, clinical features and response details were described using summary statistics, such as medians, ranges, frequencies and proportions. Progression free survival and OS analyses were conducted using the Kaplan-Meier method by cohort.

Medians and confidence intervals were reported to assess PFS and OS. Treatment related toxicity was evaluated using frequencies and proportions of adverse events based on severities and attributions. The clinical benefit rate and 95% CI for each cohort were estimated to evaluate the efficacy of treatment. Association between clinical benefit and biomarkers was evaluated using Chi-squared test or Fisher exact test. Association between biomarkers and survival outcomes was evaluated using Cox proportional hazards models. Individual patient’s changes in tumour response over time were displayed using spider plots.

### Reporting summary

Further information on research design is available in the Nature Portfolio Reporting Summary linked to this article.

## Supplementary information

Supplementary Information

Reporting Summary

## Data Availability

The individual, de-identified genomic data are deposited in the European Genome-Phenome Archive (EGA) database at https://ega-archive.org/studies/EGAS00001007013. The data are available under restricted access, access can be obtained by contacting the corresponding author (stephanie.lheureux@uhnresearch.ca). Source data for Fig. 2, Supplementary Table 1 and Supplementary Fig. 1 are provided as a Source Data file. The study protocol, including the statistical analysis plan has been uploaded as Supplementary Note 1 in the Supplementary Information file. The remaining data are available within the Article, Supplementary Information or Source Data File. Additional de-identified clinical data will be made available upon request by contacting the corresponding author. Source data are provided with this paper.

## Source data

Source Data

## References

[1] Siegel RL (2021). Cancer Statistics, 2021. CA Cancer J. Clin..

[2] Clarke MA (2019). Hysterectomy-corrected uterine corpus cancer incidence trends and differences in relative survival reveal racial disparities and rising rates of nonendometrioid cancers. J. Clin. Oncol..

[3] Madariaga A, Oza AM (2021). Wee1 inhibition in recurrent serous uterine cancer: science paving the way in a challenging disease. J. Clin. Oncol..

[4] Oaknin A (2020). Clinical activity and safety of the anti-programmed death 1 monoclonal antibody dostarlimab for patients with recurrent or advanced mismatch repair-deficient endometrial cancer: a nonrandomized phase 1 clinical trial. JAMA Oncol..

[5] Oaknin A (2020). LBA36 safety and antitumor activity of dostarlimab in patients (pts) with advanced or recurrent DNA mismatch repair deficient (dMMR) or proficient (MMRp) endometrial cancer (EC): results from GARNET. Ann. Oncol..

[6] Konstantinopoulos PA (2019). Phase II study of avelumab in patients with mismatch repair deficient and mismatch repair proficient recurrent/persistent endometrial cancer. J. Clin. Oncol..

[7] Ott PA (2017). Safety and antitumor activity of pembrolizumab in advanced programmed death ligand 1-positive endometrial cancer: results from the KEYNOTE-028 study. J. Clin. Oncol..

[8] Makker, V. et al. A multicenter, open-label, randomized, phase III study to compare the efficacy and safety of lenvatinib in combination with pembrolizumab versus treatment of physician’s choice in patients with advanced endometrial cancer. *Gynecologic Oncology* **162**, S4 (2021).

[9] Dhani NC (2020). Phase II trial of cabozantinib in recurrent/metastatic endometrial cancer: a study of the Princess Margaret, Chicago, and California Consortia (NCI9322/PHL86). Clin. Cancer Res..

[10] Lheureux, S. et al. Translational randomized phase II trial of cabozantinib in combination with nivolumab in advanced, recurrent, or metastatic endometrial cancer. *J. Immunother. Cancer.* **10**, e004233 (2022). 10.1136/jitc-2021-004233 PMC8921950 35288469

[11] Higuchi T (2015). CTLA-4 blockade synergizes therapeutically with PARP inhibition in BRCA1-deficient ovarian cancer. Cancer Immunol. Res..

[12] Brown JS, Sundar R, Lopez J (2018). Combining DNA damaging therapeutics with immunotherapy: more haste, less speed. Br. J. Cancer.

[13] Kandoth C, Cancer Genome Atlas Research N (2013). Integrated genomic characterization of endometrial carcinoma. Nature.

[14] Dedes KJ (2010). PTEN deficiency in endometrioid endometrial adenocarcinomas predicts sensitivity to PARP inhibitors. Sci. Transl. Med..

[15] Mukherjee A (2018). Nuclear PTEN localization contributes to DNA damage response in endometrial adenocarcinoma and could have a diagnostic benefit for therapeutic management of the disease. Mol. Cancer Ther..

[16] de Jonge MM (2019). Frequent homologous recombination deficiency in high-grade endometrial carcinomas. Clin. Cancer Res..

[17] Lin KK (2019). BRCA reversion mutations in circulating tumor DNA predict primary and acquired resistance to the PARP inhibitor rucaparib in high-grade ovarian carcinoma. Cancer Discov..

[18] McMullen M, Madariaga A, Lheureux S (2021). New approaches for targeting platinum-resistant ovarian cancer. Semin Cancer Biol..

[19] Madariaga, A. et al. Manage wisely: poly (ADP-ribose) polymerase inhibitor (PARPi) treatment and adverse events.. *Int. J. Gynecol. Cancer* **30**, 903–915 (2020). 10.1136/ijgc-2020-001288 PMC7398227 32276934

[20] Musacchio L (2020). PARP inhibitors in endometrial cancer: current status and perspectives. Cancer Manag. Res..

[21] Gockley AA (2018). Durable response in a woman with recurrent low-grade endometrioid endometrial cancer and a germline BRCA2 mutation treated with a PARP inhibitor. Gynecol. Oncol..

[22] Heeke A. L. et al. Prevalence of homologous recombination-related gene mutations across multiple cancer types. *JCO Precis. Oncol.* **2018**, PO.17.00286 10.1200/PO.17.00286 (2018). 10.1200/PO.17.00286 PMC6139373 30234181

[23] Ngoi NYL, Tan DSP (2021). The role of homologous recombination deficiency testing in ovarian cancer and its clinical implications: do we need it?. ESMO Open.

[24] Swisher EM (2021). Characterization of patients with long-term responses to rucaparib treatment in recurrent ovarian cancer. Gynecol. Oncol..

[25] Swisher EM (2021). Molecular and clinical determinants of response and resistance to rucaparib for recurrent ovarian cancer treatment in ARIEL2 (Parts 1 and 2). Nat. Commun..

[26] Garg K (2012). Pathologic scoring of PTEN immunohistochemistry in endometrial carcinoma is highly reproducible. Int J. Gynecol. Pathol..

[27] Wang, L. et al. Immunohistochemistry and next-generation sequencing are complementary tests in identifying PTEN abnormality in endometrial carcinoma biopsies. *Int. J. Gynecol. Pathol.* **41**, 12–19 (2022). 10.1097/PGP.0000000000000763 33720084

[28] Kir G (2020). Correlation of PD-L1 expression with immunohistochemically determined molecular profile in endometrial carcinomas. Virchows Arch..

[29] Smith D (2021). Tumor-associated immune cells and progression-free survival in advanced endometrial cancer (EC), results from the PHAEDRA trial (ANZGOG 1601). J. Clin. Oncol..

[30] Marabelle A (2020). Association of tumour mutational burden with outcomes in patients with advanced solid tumours treated with pembrolizumab: prospective biomarker analysis of the multicohort, open-label, phase 2 KEYNOTE-158 study. Lancet Oncol..

[31] Samstein RM (2019). Tumor mutational load predicts survival after immunotherapy across multiple cancer types. Nat. Genet..

[32] Konstantinopoulos PA (2022). Evaluation of treatment with talazoparib and avelumab in patients with recurrent mismatch repair proficient endometrial cancer. JAMA Oncol..

[33] Rimel BJ (2021). A randomized, phase ii study comparing single-agent olaparib, single agent cediranib, and the combination of cediranib/olaparib in women with recurrent, persistent or metastatic endometrial cancer. Gynecol. Oncol..

[34] You, B. et al. Abstract CT005: Safety and efficacy of olaparib combined to metronomic cyclophosphamide and metformin in recurrent advanced/metastatic endometrial cancer patients: ENDOLA trial. *Cancer Res.* **82**, CT005 (2022).

[35] Madariaga A, Goodwin PJ, Oza AM (2020). Metformin in gynecologic cancers: opening a new window for prevention and treatment?. Clin. Cancer Res..

[36] Liu JF (2021). Phase II study of the WEE1 inhibitor adavosertib in recurrent uterine serous carcinoma. J. Clin. Oncol..

[37] Spinosa, D. et al. To test or re-test, that is the question: Comparison of the mismatch repair deficiency between primary and metastatic sites of uterine cancers. Presented at SGO 2022; 18–21, (2022).

[38] Moss EL (2020). Utility of circulating tumor DNA for detection and monitoring of endometrial cancer recurrence and progression. Cancers.

[39] Jaiswal, S. & Ebert, B. L. Clonal hematopoiesis in human aging and disease. *Science* **366**, eaan4673 (2019). 10.1126/science.aan4673 PMC8050831 31672865

[40] Lheureux S (2018). A clinical and molecular phase II trial of oral ENMD-2076 in ovarian clear cell carcinoma (OCCC): a study of the Princess Margaret Phase II Consortium. Clin. Cancer Res..

[41] Li J (2012). CONTRA: copy number analysis for targeted resequencing. Bioinformatics.

