# Supplementary material for: Clinical outcome and biomarker assessments of a multi-centre phase II trial assessing niraparib with or without dostarlimab in recurrent endometrial carcinoma
Source: Nat Commun. 2023 Mar 15;14:1452. doi: 10.1038/s41467-023-37084-w (PMC10017680; doi:10.1038/s41467-023-37084-w)
Supplement: Supplementary file 1 — Supplementary Information [file 41467_2023_37084_MOESM1_ESM.pdf]

Cohort 1 – Niraparib (n=25)

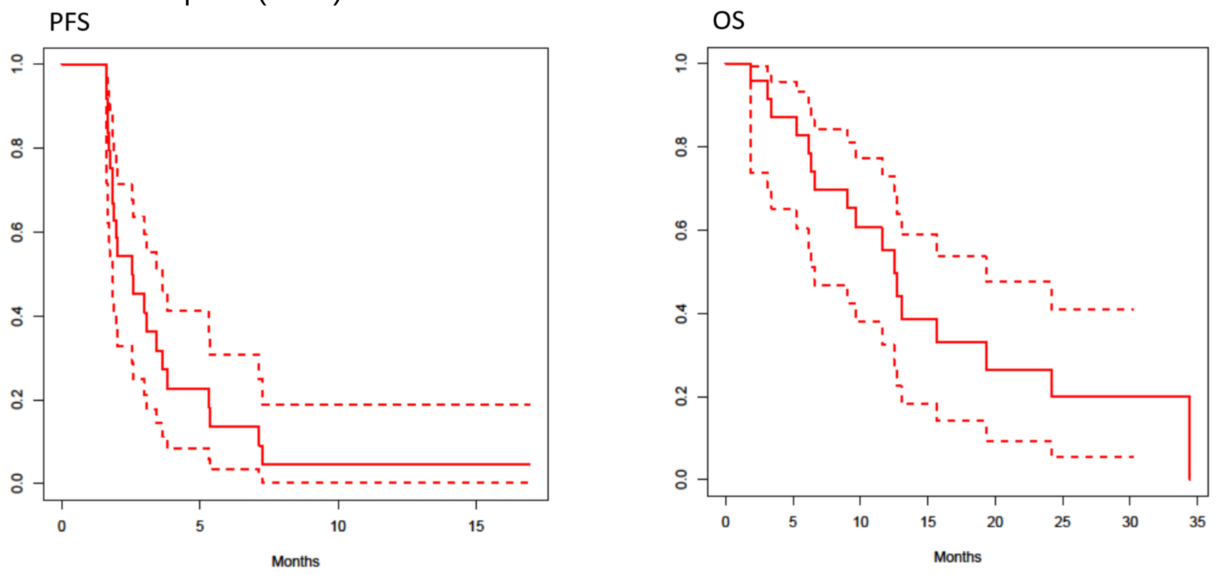

Cohort 2 – Niraparib + Dostarlimab (n=22)

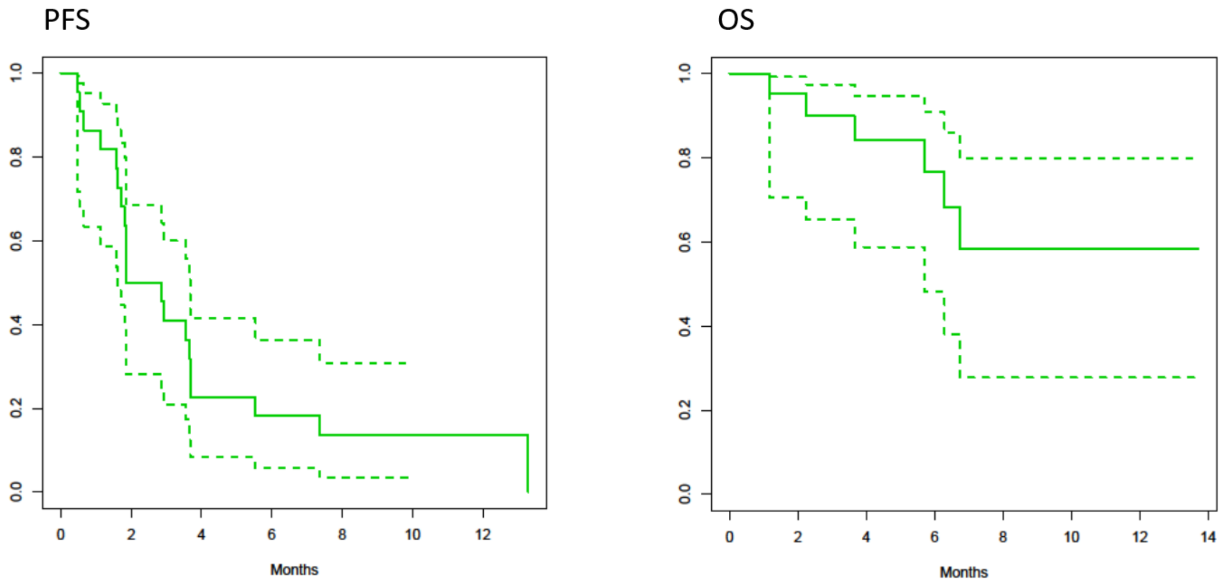

Supplementary Figure 1. Progression free survival and overall survival per cohort.

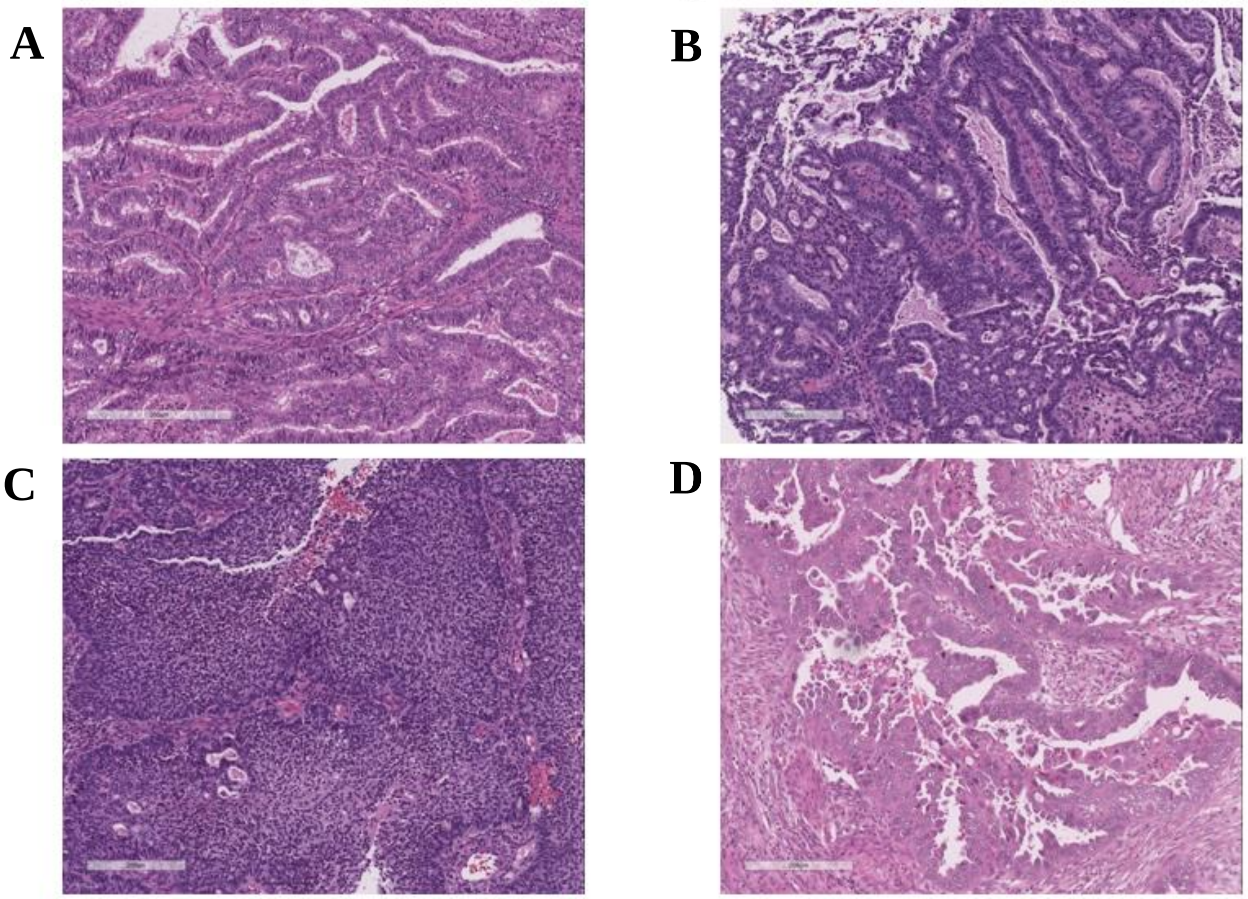

E

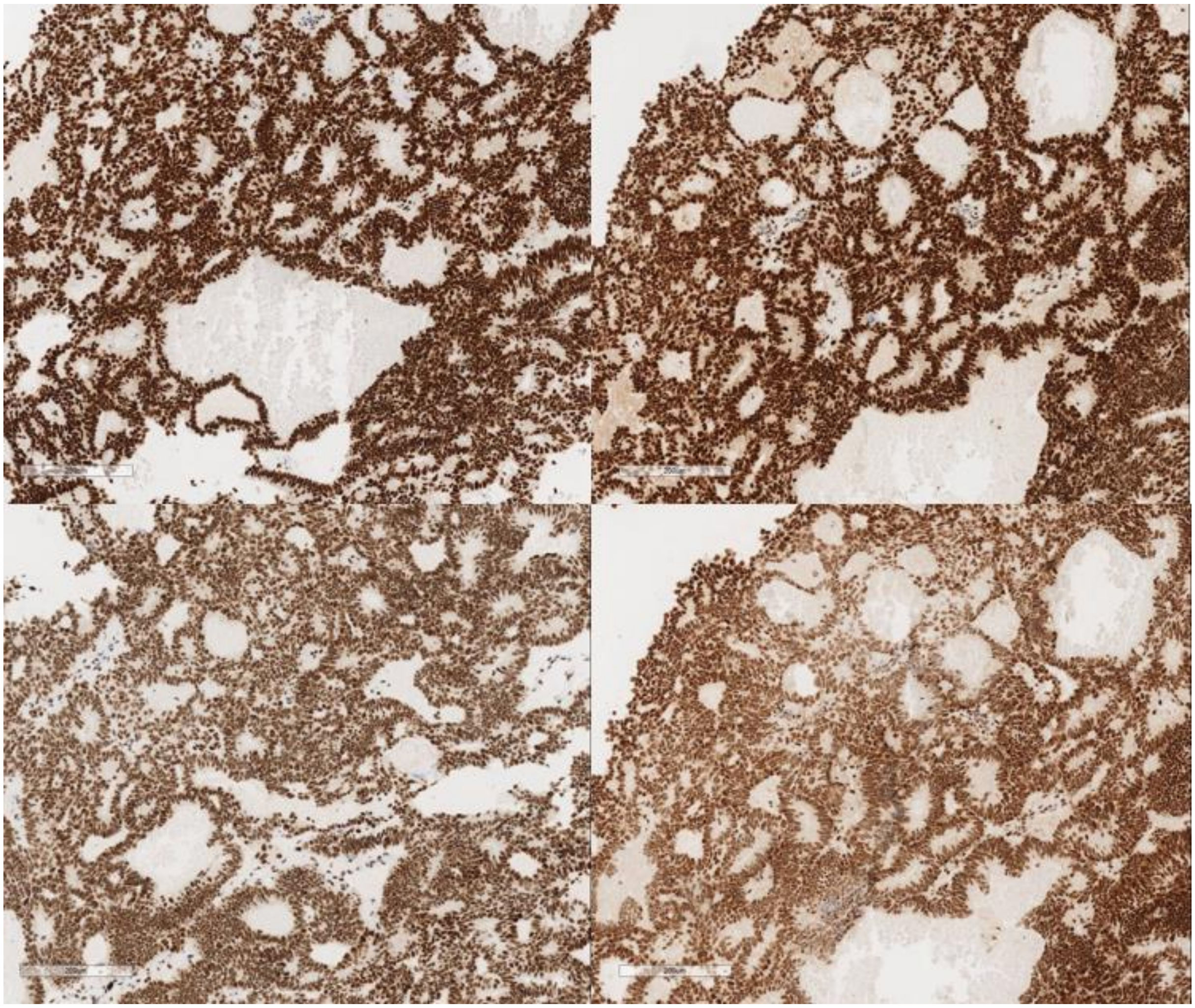

F

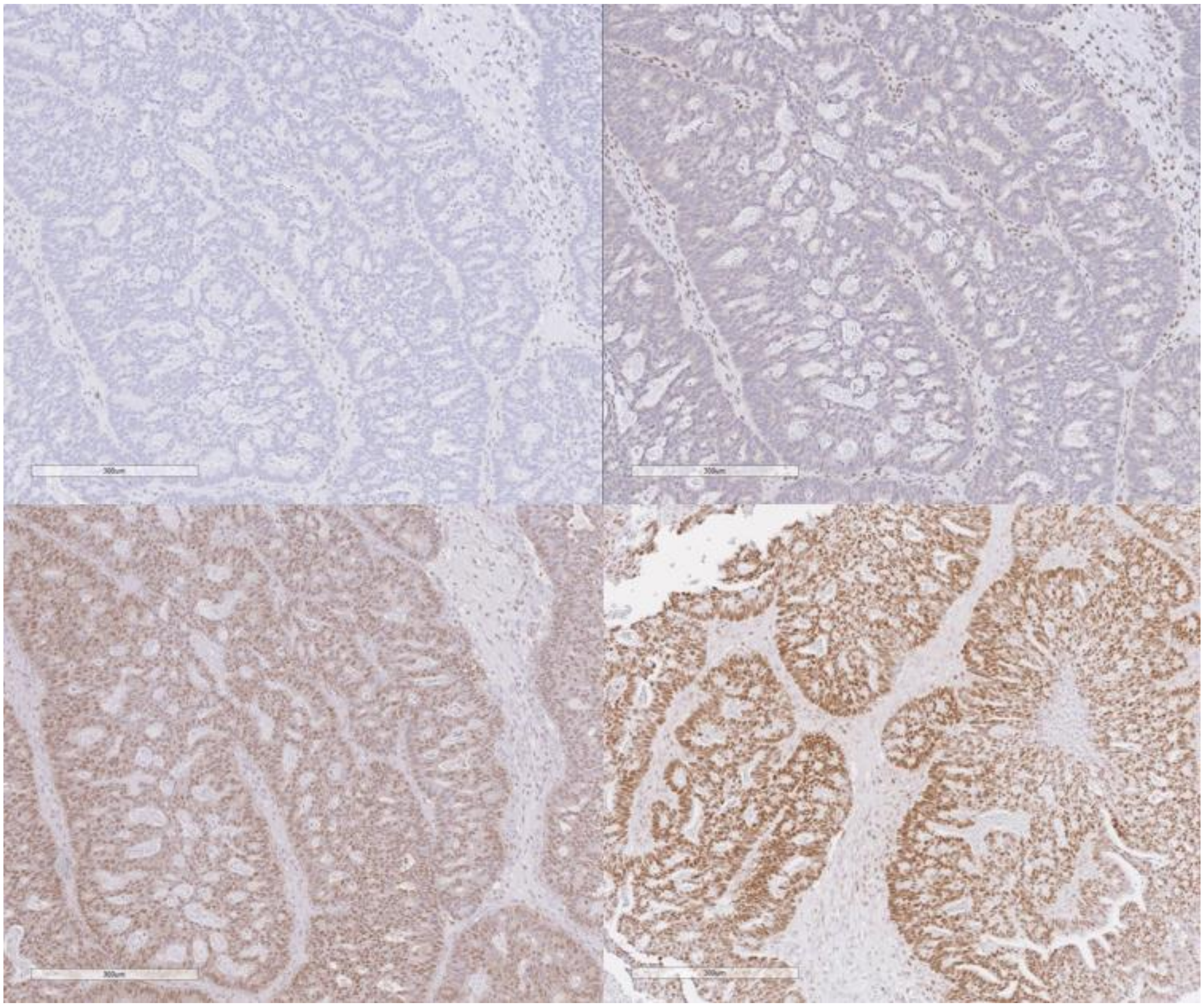

G

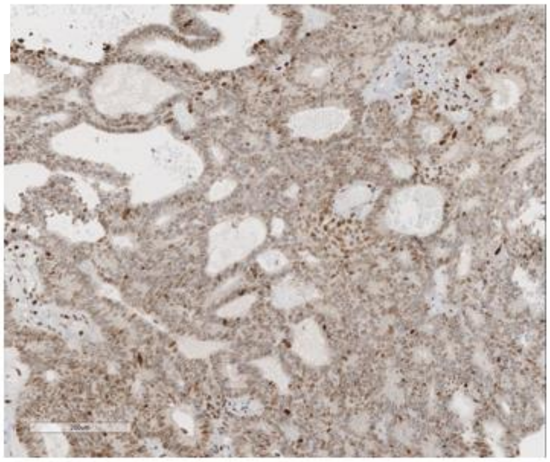

H

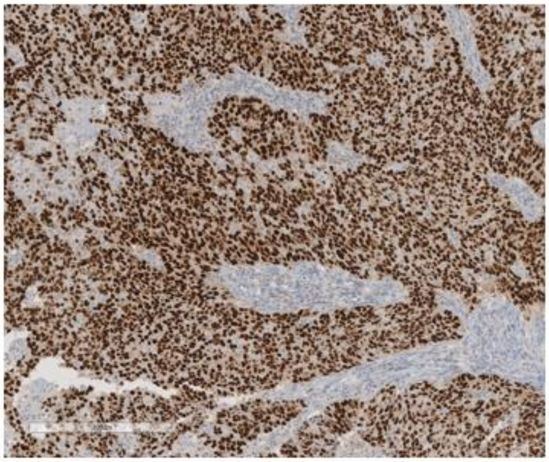

I

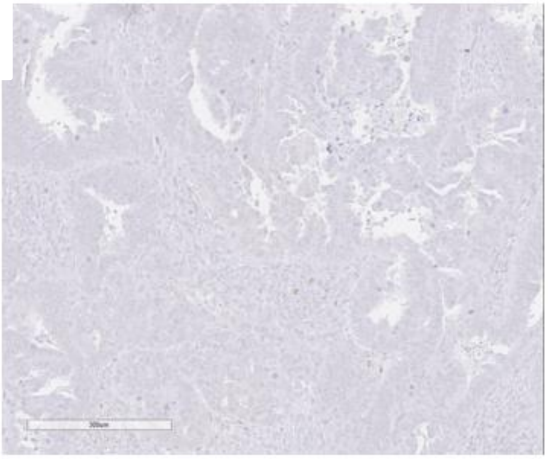

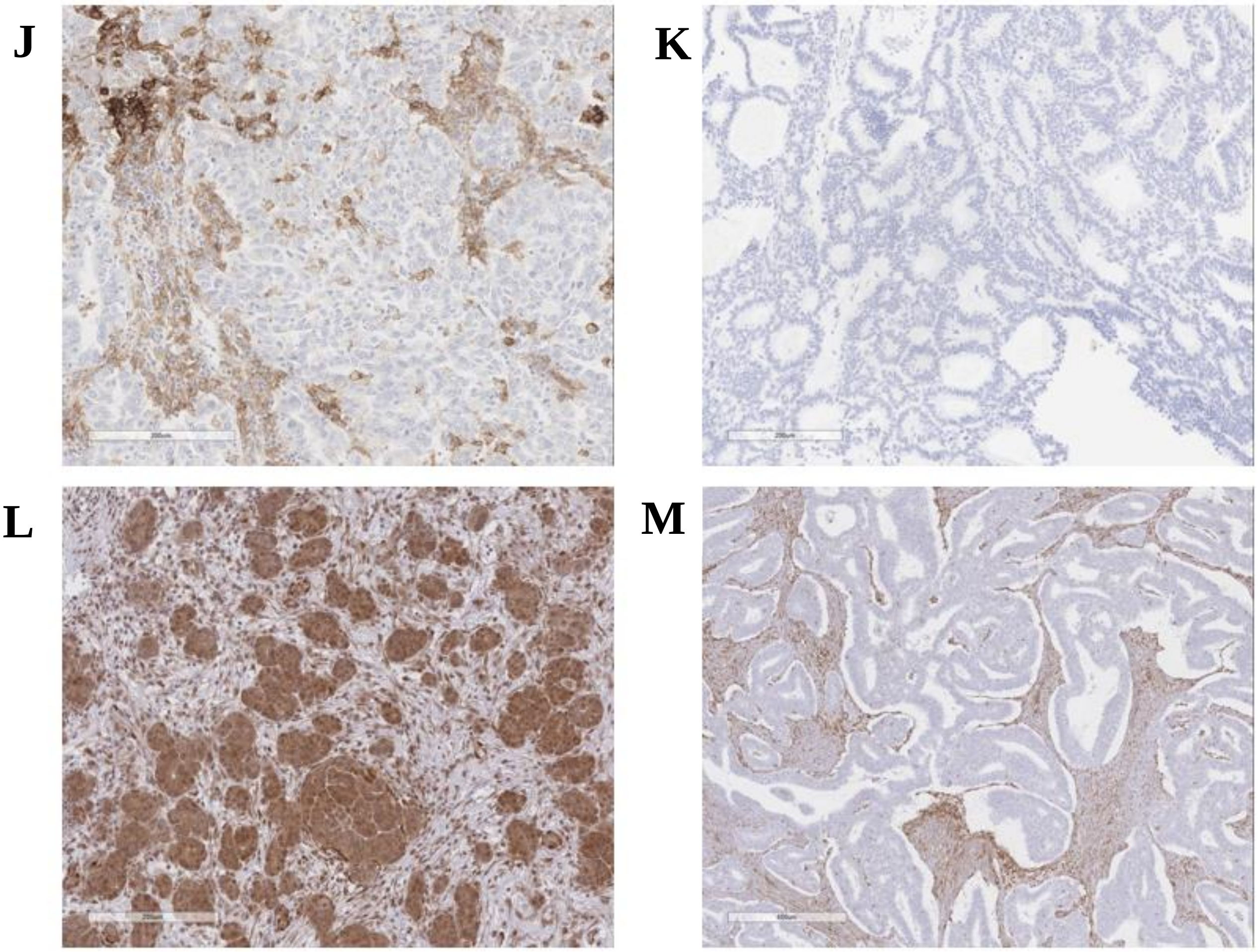

Supplementary Figure 2. Immunohistochemistry markers used in the study.

A) Endometrioid endometrial carcinoma, FIGO grade 1, showing <5% solid growth (H&E stain)
B) Endometrioid endometrial carcinoma, FIGO grade 2, showing between 5% and 50% solid/microacinar growth (H&E stain)
C) Endometrioid endometrial carcinoma, FIGO grade 3, showing >50% solid/microacinar growth (H&E stain)
D) Serous carcinoma, characterized by papillary/tufted architecture, irregular luminal borders, high-grade cytologic atypia and brisk mitotic activity (H&E stain)
E) Mismatch repair proficient (MMRp) endometrial carcinoma – intact nuclear staining seen in tumor cells stained with MLH1, PMS2, MSH2 and MSH6 (clockwise from top left)
F) Mismatch repair deficient (MMRd) endometrial carcinoma – loss of nuclear staining with MLH1 and PMS2 (note the retained staining in peritumoral stromal and inflammatory cells) with intact nuclear expression of MSH2 and MSH6 (clockwise from top left)
G) Normal p53 staining (wild-type pattern) in endometrial carcinoma, whereby a proportion of tumor nuclei show positive nuclear expression of variable intensity
H) Abnormal p53 staining (overexpression pattern) in endometrial carcinoma, in which >80% of tumor nuclei show strong positivity

I) Abnormal p53 staining (null pattern) in endometrial carcinoma; no staining is seen in is seen in tumor cell nuclei. Note the presence of faint wild-type staining in background non-tumor nuclei, serving as an internal positive control.
J) Positive PD-L1 staining in endometrial carcinoma, predominantly seen in intratumoral immune cells, with lesser expression in viable tumor cells
K) PD-L1-negative endometrial carcinoma
L) Endometrial carcinoma with retained PTEN staining
M) Loss of PTEN expression in endometrial carcinoma; note retained staining in peritumoral stroma, serving as a positive internal control

	**Cohort**	**n (%)**	**Serous**	**Endometrioid low grade**	**Endometrioid high grade**	**Mixed**
**PTEN IHC status**	C1	Retained	8 (57%)	4 (67%)	0 (0%)	1 (100%)
		Lost	4 (29%)	1 (17%)	3 (100%)	0 (0%)
		Heterogeneous	2 (14%)	1 (17%)	0 (0%)	0 (0%)
		NA	1	0	0	0
	C2	Retained	6 (67%)	1 (20%)	4 (57%)	0 (0%)
		Lost	3 (33%)	4 (80%)	3 (43%)	1 (100%)
		Heterogeneous	0 (0%)	0 (0%)	0 (0%)	0 (0%)
		NA	0	0	0	0
	Overall	Retained	14 (61%)	5 (45%)	4 (40%)	1 (50%)
		Lost	7 (30%)	5 (45%)	6 (60%)	1 (50%)
		Heterogeneous	2 (8.7%)	1 (9.1%)	0 (0%)	0 (0%)
		NA	1	0	0	0
***PTEN* mutation**	C1	no	10 (83%)	2 (40%)	1 (33%)	1 (100%)
		yes	2 (17%)	3 (60%)	2 (67%)	0 (0%)
		NA	3	1	0	0
	C2	no	9 (100%)	1 (20%)	3 (43%)	0 (0%)
		yes	0 (0%)	4 (80%)	4 (57%)	1 (100%)
		NA	0	0	0	0
	Overall	no	19 (90%)	3 (30%)	4 (40%)	1 (50%)
		yes	2 (9.5%)	7 (70%)	6 (60%)	1 (50%)
		NA	3	1	0	0
***PTEN* mutation with oncogenicity as per OncoKB**	C1	Negative	10 (83%)	3 (60%)	1 (33%)	1 (100%)
		Oncogenic	2 (17%)	2 (40%)	2 (67%)	0 (0%)
		NA	3	1	0	0
	C2	Negative	9 (100%)	1 (20%)	3 (43%)	0 (0%)
		Oncogenic	0 (0%)	4 (80%)	4 (57%)	1

						(100%)
		NA	0	0	0	0
	Overall	Negative	19 (90%)	4 (40%)	4 (40%)	1 (50%)
		Oncogenic	2 (9.5%)	6 (60%)	6 (60%)	1 (50%)
		NA	3	1	0	0
**MMR status**	C1	Intact	12 (92%)	3 (50%)	2 (67%)	1 (100%)
		Deficient	0 (0%)	3 (50%)	1 (33%)	0 (0%)
		Equivocal (Subclonal)	1 (7.7%)	0 (0%)	0 (0%)	0 (0%)
		NA	2	0	0	0
	C2	Intact	9 (100%)	2 (40%)	7 (100%)	1 (100%)
		Deficient	0 (0%)	3 (60%)	0 (0%)	0 (0%)
		Equivocal (Subclonal)	0 (0%)	0 (0%)	0 (0%)	0 (0%)
		NA	0	0	0	0
	Overall	Intact	21 (95%)	5 (45%)	9 (90%)	2 (100%)
		Deficient	0 (0%)	6 (55%)	1 (10%)	0 (0%)
		Equivocal (Subclonal)	1 (4.5%)	0 (0%)	0 (0%)	0 (0%)
		NA	2	0	0	0
**PDL1 status**	C1	Negative	9 (64%)	5 (83%)	0 (0%)	0 (0%)
		Positive	5 (36%)	1 (17%)	3 (100%)	1 (100%)
		NA	1	0	0	0
	C2	Negative	2 (22%)	2 (40%)	3 (43%)	1 (100%)
		Positive	7 (78%)	3 (60%)	4 (57%)	0 (0%)
		NA	0	0	0	0
	Overall	Negative	11 (48%)	7 (64%)	3 (30%)	1 (50%)
		Positive	12 (52%)	4 (36%)	7 (70%)	1 (50%)
		NA	1	0	0	0
**p53 IHC**	C1	Abnormal	14 (100%)	0 (0%)	0 (0%)	0 (0%)
		Wild-type	0 (0%)	6 (100%)	2 (100%)	0 (0%)
		Overexpressed	0 (0%)	0 (0%)	0 (0%)	1 (100%)
		NA	1	0	1	0
	C2	Abnormal	9 (100%)	0 (0%)	2 (29%)	1 (100%)
		Wild-type	0 (0%)	5 (100%)	5 (71%)	0 (0%)
		Overexpressed	0 (0%)	0 (0%)	0 (0%)	0 (0%)
		NA	0	0	0	0

	Overall	Abnormal	23 (100%)	0 (0%)	2 (22%)	1 (50%)
		Wild-type	0 (0%)	11 (100%)	7 (78%)	0 (0%)
		Overexpressed	0 (0%)	0 (0%)	0 (0%)	1 (50%)
		NA	1	0	1	0
***TP53* mutation**	C1	yes	12 (100%)	0 (0%)	3 (100%)	1 (100%)
		no	0 (0%)	5 (100%)	0 (0%)	0 (0%)
		NA	3	1	0	0
	C2	yes	9 (100%)	0 (0%)	2 (29%)	1 (100%)
		no	0 (0%)	5 (100%)	5 (71%)	0 (0%)
		NA	0	0	0	0
	Overall	yes	21 (100%)	0 (0%)	5 (50%)	2 (100%)
		no	0 (0%)	10 (100%)	5 (50%)	0 (0%)
		NA	3	1	0	0
***TP53* mutation with oncogenicity as per OncoKB**	C1	Oncogenic	11 (92%)	0 (0%)	2 (67%)	0 (0%)
		Negative	0 (0%)	5 (100%)	0 (0%)	0 (0%)
		VUS	1 (8.3%)	0 (0%)	1 (33%)	1 (100%)
		NA	3	1	0	0
	C2	Oncogenic	9 (100%)	0 (0%)	2 (29%)	1 (100%)
		Negative	0 (0%)	5 (100%)	5 (71%)	0 (0%)
		VUS	0 (0%)	0 (0%)	0 (0%)	0 (0%)
		NA	0	0	0	0
	Overall	Oncogenic	20 (95%)	0 (0%)	4 (40%)	1 (50%)
		Negative	0 (0%)	10 (100%)	5 (50%)	0 (0%)
		VUS	1 (4.8%)	0 (0%)	1 (10%)	1 (50%)
		NA	3	1	0	0
**HRR gene alterations**	C1	yes	7 (58%)	4 (80%)	2 (67%)	0 (0%)
		No	5 (42%)	1 (20%)	1 (33%)	1 (100%)
		NA	3	1	0	0
	C2	yes	6 (67%)	3 (60%)	6 (86%)	1 (100%)
		No	3 (33%)	2 (40%)	1 (14%)	0 (0%)
		NA	0	0	0	0
	Overall	yes	13 (62%)	7 (70%)	8 (80%)	1 (50%)
		No	8 (38%)	3 (30%)	2 (20%)	1 (50%)
		NA	3	1	0	0
**HRR alterations**	C1	Negative	9 (75%)	1 (20%)	2 (67%)	1 (100%)

**with oncogenicity as per OncoKB**		Oncogenic	3 (25%)	4 (80%)	1 (33%)	0 (0%)
		NA	3	1	0	0
	C2	Negative	7 (78%)	3 (60%)	2 (29%)	0 (0%)
		Oncogenic	2 (22%)	2 (40%)	5 (71%)	1 (100%)
		NA	0	0	0	0
	Overall	Negative	16 (76%)	4 (40%)	4 (40%)	1 (50%)
		Oncogenic	5 (24%)	6 (60%)	6 (60%)	1 (50%)
		NA	3	1	0	0
**HRR alterations, excluding *ARID1A*, with oncogenicity as per OncoKB**	C1	negative	11 (92%)	3 (60%)	2 (67%)	1 (100%)
		positive	1 (8.3%)	2 (40%)	1 (33%)	0 (0%)
		NA	3	1	0	0
	C2	negative	7 (78%)	4 (80%)	3 (43%)	1 (100%)
		positive	2 (22%)	1 (20%)	4 (57%)	0 (0%)
		NA	0	0	0	0
	Overall	negative	18 (86%)	7 (70%)	5 (50%)	2 (100%)
		positive	3 (14%)	3 (30%)	5 (50%)	0 (0%)
		NA	3	1	0	0
**TMB high**	C1	no	11 (92%)	2 (40%)	3 (100%)	1 (100%)
		yes	1 (8.3%)	3 (60%)	0 (0%)	0 (0%)
		NA	3	1	0	0
	C2	no	8 (89%)	4 (80%)	4 (57%)	1 (100%)
		yes	1 (11%)	1 (20%)	3 (43%)	0 (0%)
		NA	0	0	0	0
	Overall	no	19 (90%)	6 (60%)	7 (70%)	2 (100%)
		yes	2 (9.5%)	4 (40%)	3 (30%)	0 (0%)
		NA	3	1	0	0

Supplementary Table 1. Immunohistochemistry and genomic alterations per histological subtype. Data is presented per cohort, and overall.
C1: Cohort 1, C2: Cohort 2, NA: Not available.

**Study number**	**New variants seen in ctDNA alone**
NEC-005	CHEK2 (NM_007194.3) c.283C>T (p.Arg95*, aka p.R95*) AF: 1.7%, likely onc
NEC-012	TP53 (NM_000546.5) c.808T>C (p.Phe270Leu, aka p.F270L) AF: 1.5%; VUS

NEC-016	ATR (NM_001184.3) c.6793G>A (p.Val2265Ile, aka p.V2265I) AF: 1.4%; VUS
NEC-022	FANCD2 (NM_033084.3) c.3931C>T (p.Pro1311Ser, aka p.P1311S) AF: 1.0%; VUS
NEC-027	TP53 (NM_000546.5) c.827C>A (p.Ala276Asp, aka p.A276D) AF: 11.7%; VUS
NEC-032	ATRX (NM_000489.4) c.2677G>A (p.Gly893Ser, aka p.G893S) AF: 1.9%; VUS

Supplementary Table 2. New variants seen in baseline ctDNA, not detected in archival tissue, in cohort 1 (n=5, NEC-005 to NEC-027) and cohort 2 (n=1, NEC-032).

**NEC study number**	**1st ctDNA sample**	**2nd ctDNA sample**
NEC-011	no variant detected	no variants detected
NEC-016	**ATR (NM_001184.3) c.6793G>A (p.Val2265Ile, aka p.V2265I) AF: 1.4%**<br>BAP1 (NM_004656.2) c.878C>T (p.Pro293Leu, aka p.P293L) AF: 45.6%<br>FANCA (NM_000135.2) c.308C>T (p.Ser103Leu, aka p.S103L) AF: 47.0%<br>NBN (NM_002485.4) c.1361C>A (p.Ser454Tyr, aka p.S454Y) AF: 44.6%	**ATR (NM_001184.3) c.6793G>A (p.Val2265Ile, aka p.V2265I) AF: 3.4%**<br>BAP1 (NM_004656.2) c.878C>T (p.Pro293Leu, aka p.P293L) AF: 44.7%<br>FANCA (NM_000135.2) c.308C>T (p.Ser103Leu, aka p.S103L) AF: 47.9%<br>NBN (NM_002485.4) c.1361C>A (p.Ser454Tyr, aka p.S454Y) AF: 46.7%
NEC-044	**PTEN (NM_000314.4) c.277C>T (p.His93Tyr, aka p.H93Y) AF: 2.8%; Onc**<br>**ARID1A (NM_006015.4) c.4495C>T (p.Gln1499*, aka p.Q1499*) AF: 2.4%; Onc**<br>**ARID1A (NM_006015.4) c.4856del (p.Pro1619Glnfs*7, aka p.P1619Qfs*7) AF: 1.8%; Onc**<br>**PTEN (NM_000314.4) c.697C>T (p.Arg233*, aka p.R233*) AF: 2.6%; Onc**<br>**ATRX (NM_000489.4) c.110G>A (p.Arg37Gln, aka p.R37Q) AF: 2.1%; VUS**<br>BRCA2 (NM_000059.3) c.7522G>A (p.Gly2508Ser, aka p.G2508S) AF: 46.8%; VUS<br>FANCL (NM_018062.3) c.677G>A (p.Arg226His, aka p.R226H) AF: 50.5%; VUS	BRCA2 (NM_000059.3) c.7522G>A (p.Gly2508Ser, aka p.G2508S) AF: 48.8%<br>FANCL (NM_018062.3) c.677G>A (p.Arg226His, aka p.R226H) AF: 48.0%

NEC-045	**PTEN (NM_000314.4) c.518G>A (p.Arg173His, aka p.R173H) AF: 1.4%**<br>ARID1A (NM_006015.4) c.5965C>T (p.Arg1989*, aka p.R1989*) AF: 85.2%<br>ATM (NM_000051.3) c.2002G>T (p.Glu668*, aka p.E668*) AF: 34.0%<br>ATM (NM_000051.3) c.2510C>A (p.Ser837*, aka p.S837*) AF: 39.5%<br>**ATR (NM_001184.3) c.7075G>T (p.Glu2359*, aka p.E2359*) AF: 3.1%**<br>**ATR (NM_001184.3) c.7756G>T (p.Glu2586*, aka p.E2586*) AF: 1.3%**<br>ATRX (NM_000489.4) c.271G>T (p.Glu91*, aka p.E91*) AF: 21.0%<br>ATRX (NM_000489.4) c.6997G>T (p.Glu2333*, aka p.E2333*) AF: 28.9%<br>BLM (NM_000057.2) c.503C>A (p.Ser168*, aka p.S168*) AF: 8.0%<br>**BRCA1 (NM_007294.3) c.2158G>T (p.Glu720*, aka p.E720*) AF: 4.1%**<br>BRCA2 (NM_000059.3) c.289G>T (p.Glu97*, aka p.E97*) AF: 34.5%<br>BRCA2 (NM_000059.3) c.4552G>T (p.Glu1518*, aka p.E1518*) AF: 38.7%<br>BRCA2 (NM_000059.3) c.6082G>T (p.Glu2028*, aka p.E2028*) AF: 36.2%<br>BRCA2 (NM_000059.3) c.926C>A (p.Ser309*, aka p.S309*) AF: 36.3%<br>CHEK2 (NM_007194.3) c.235G>T (p.Glu79*, aka p.E79*) AF: 6.5%<br>**MRE11A (NM_005591.3) c.571C>T (p.Arg191*, aka p.R191*) AF: 15.4%**<br>POLE (NM_006231.3) c.1270C>A (p.Leu424Ile, aka p.L424I) AF: 41.2%<br>PTEN (NM_000314.4) c.388C>T (p.Arg130*, aka p.R130*) AF: 36.7%<br>TP53 (NM_000546.5) c.637C>T (p.Arg213*, aka p.R213*) AF: 40.5%<br>TP53 (NM_000546.5) c.916C>T (p.Arg306*, aka p.R306*) AF: 41.4%<br>Additional Variants:<br>ARID1A (NM_006015.4) c.148A>G (p.Met50Val, aka p.M50V) AF: 53.8%<br>ATM (NM_000051.3) c.7199G>T (p.Arg2400Ile, aka p.R2400I) AF: 37.8%<br>ATR (NM_001184.3) c.440A>C (p.Lys147Thr, aka p.K147T) AF: 39.3%<br>**ATR (NM_001184.3) c.6208C>A (p.Leu2070Ile, aka p.L2070I) AF: 18.3%**<br>**ATRX (NM_000489.4) c.1671G>T (p.Glu557Asp, aka p.E557D) AF: 1.2%**<br>ATRX (NM_000489.4) c.1964G>T (p.Arg655Ile, aka p.R655I) AF: 32.7%	POLE (NM_006231.3) c.5609G>T (p.Arg1870Leu, aka p.R1870L) AF: 48.1%<br>RAD51C (NM_058216.1) c.640C>T (p.Arg214Cys, aka p.R214C) AF: 46.0%

	**ATRX (NM_000489.4) c.2716G>T (p.Asp906Tyr, aka p.D906Y) AF: 7.0%**<br>ATRX (NM_000489.4) c.2958A>C (p.Lys986Asn, aka p.K986N) AF: 32.1%<br>**ATRX (NM_000489.4) c.3804G>T (p.Glu1268Asp, aka p.E1268D) AF: 7.9%**<br>**ATRX (NM_000489.4) c.4441C>T (p.Arg1481Trp, aka p.R1481W) AF: 7.2%**<br>ATRX (NM_000489.4) c.6236G>A (p.Arg2079Gln, aka p.R2079Q) AF: 27.0%<br>ATRX (NM_000489.4) c.7210T>G (p.Cys2404Gly, aka p.C2404G) AF: 4.3%<br>**BARD1 (NM_000465.2) c.1409A>G (p.Asn470Ser, aka p.N470S) AF: 6.0%**<br>BRCA1 (NM_007294.3) c.3237A>C (p.Lys1079Asn, aka p.K1079N) AF: 41.3%<br>**BRCA1 (NM_007294.3) c.7T>G (p.Leu3Val, aka p.L3V) AF: 1.7%**<br>BRCA2 (NM_000059.3) c.3668A>G (p.His1223Arg, aka p.H1223R) AF: 5.5%<br>BRCA2 (NM_000059.3) c.4416G>T (p.Lys1472Asn, aka p.K1472N) AF: 35.1%<br>BRCA2 (NM_000059.3) c.4676T>G (p.Phe1559Cys, aka p.F1559C) AF: 37.6%<br>BRCA2 (NM_000059.3) c.4949G>T (p.Ser1650Ile, aka p.S1650I) AF: 30.0%<br>**BRCA2 (NM_000059.3) c.7850G>T (p.Arg2617Ile, aka p.R2617I) AF: 12.6%**<br>**BRCA2 (NM_000059.3) c.8618T>G (p.Phe2873Cys, aka p.F2873C) AF: 5.3%**<br>BRIP1 (NM_032043.2) c.3723T>G (p.Asn1241Lys, aka p.N1241K) AF: 26.7%<br>BRIP1 (NM_032043.2) c.3734T>G (p.Phe1245Cys, aka p.F1245C) AF: 24.1%<br>**BRIP1 (NM_032043.2) c.479G>T (p.Arg160Ile, aka p.R160I) AF: 5.6%**<br>**CHEK1 (NM_001274.5) c.1136G>A (p.Arg379Gln, aka p.R379Q) AF: 1.2%**<br>**CHEK1 (NM_001274.5) c.215G>A**	

	**(p.Gly72Asp, aka p.G72D) AF: 7.2%**<br>**FANCA (NM_000135.2) c.334C>A (p.Leu112Ile, aka p.L112I) AF: 1.0%**<br>**FANCC (NM_000136.2) c.238A>C (p.Ile80Leu, aka p.I80L) AF: 14.3%**<br>**FANCD2 (NM_033084.3) c.4373T>C (p.Val1458Ala, aka p.V1458A) AF: 5.8%**<br>**MRE11A (NM_005591.3) c.1380A>C (p.Glu460Asp, aka p.E460D) AF: 12.3%**<br>**MRE11A (NM_005591.3) c.391G>A (p.Asp131Asn, aka p.D131N) AF: 5.8%**<br>**NBN (NM_002485.4) c.927A>T (p.Glu309Asp, aka p.E309D) AF: 1.6%**<br>POLE (NM_006231.3) c.1269T>A (p.Asn423Lys, aka p.N423K) AF: 41.3%<br>POLE (NM_006231.3) c.3446C>T (p.Ala1149Val, aka p.A1149V) AF: 35.9%<br>POLE (NM_006231.3) c.5609G>T (p.Arg1870Leu, aka p.R1870L) AF: 43.9%<br>PTEN (NM_000314.4) c.424C>T (p.Arg142Trp, aka p.R142W) AF: 42.6%<br>PTEN (NM_000314.4) c.460T>G (p.Phe154Val, aka p.F154V) AF: 40.6%<br>**RAD50 (NM_005732.3) c.3080G>T (p.Arg1027Ile, aka p.R1027I) AF: 11.3%**<br>RAD51C (NM_058216.1) c.640C>T (p.Arg214Cys, aka p.R214C) AF: 42.7%<br>**FANCF (NM_022725.3) c.459G>T (p.Glu153Asp, aka p.E153D) AF: 7.2%**<br>FANCL (NM_018062.3) c.288G>T (p.Lys96Asn, aka p.K96N) AF: 34.8%<br>**FANCL (NM_018062.3) c.358A>C (p.Thr120Pro, aka p.T120P) AF: 5.7%**<br>WRN (NM_000553.4) c.1486A>C (p.Lys496Gln, aka p.K496Q) AF: 22.4%<br>**WRN (NM_000553.4) c.1781T>G (p.Val594Gly, aka p.V594G) AF: 2.3%**<br>WRN (NM_000553.4) c.269A>C (p.Asn90Thr, aka p.N90T) AF: 39.2%	

Supplementary Table 3. Variants detected in patients were two ctDNA samples were tested.

All patients were long response to treatment with PFS> 6 months (n=2, NEC-011 and NEC-016 from cohort 1; n=2, NEC-044 and NEC-045 from cohort 2).
The second sample from NEC-011 and NEC-016 was collected at the time of progression. The second sample from NEC-044 and NEC-045 was collected in patients with ongoing response to therapy. Variants detected with VAF<20% are marked in **bold.**

Supplementary Note 1. Study protocol.

Protocol Version/ Date: Initial/15 December 2016
A1 / 15 January 2018
A2 / 11 Jan 2019
A3 / 23 Apr 2020

TITLE: A Phase II, Open label Study of the Poly(ADP-ribose) Polymerase Inhibitor Niraparib in monotherapy or in combination with anti-PD1 inhibitor TSR-042 in Recurrent Endometrial Cancer

Coordinating Center: **Drug Development Program, Princess Margaret Cancer Centre**
Principal Investigator: Dr. Stephanie Lheureux
[redacted]

***Sponsor and Scientific Director:** Dr. Amit Oza
[redacted]

Participating Centers [redacted]

[redacted]

[redacted]

[redacted]

[redacted]

[redacted]

[redacted]

Medical Oncology Fellow:
Dr Ainhoa Madariaga
[redacted]

Statistician:
Lisa Wang
[redacted]

Central Office Coordinator:
[redacted]

[redacted]

SCHEMA

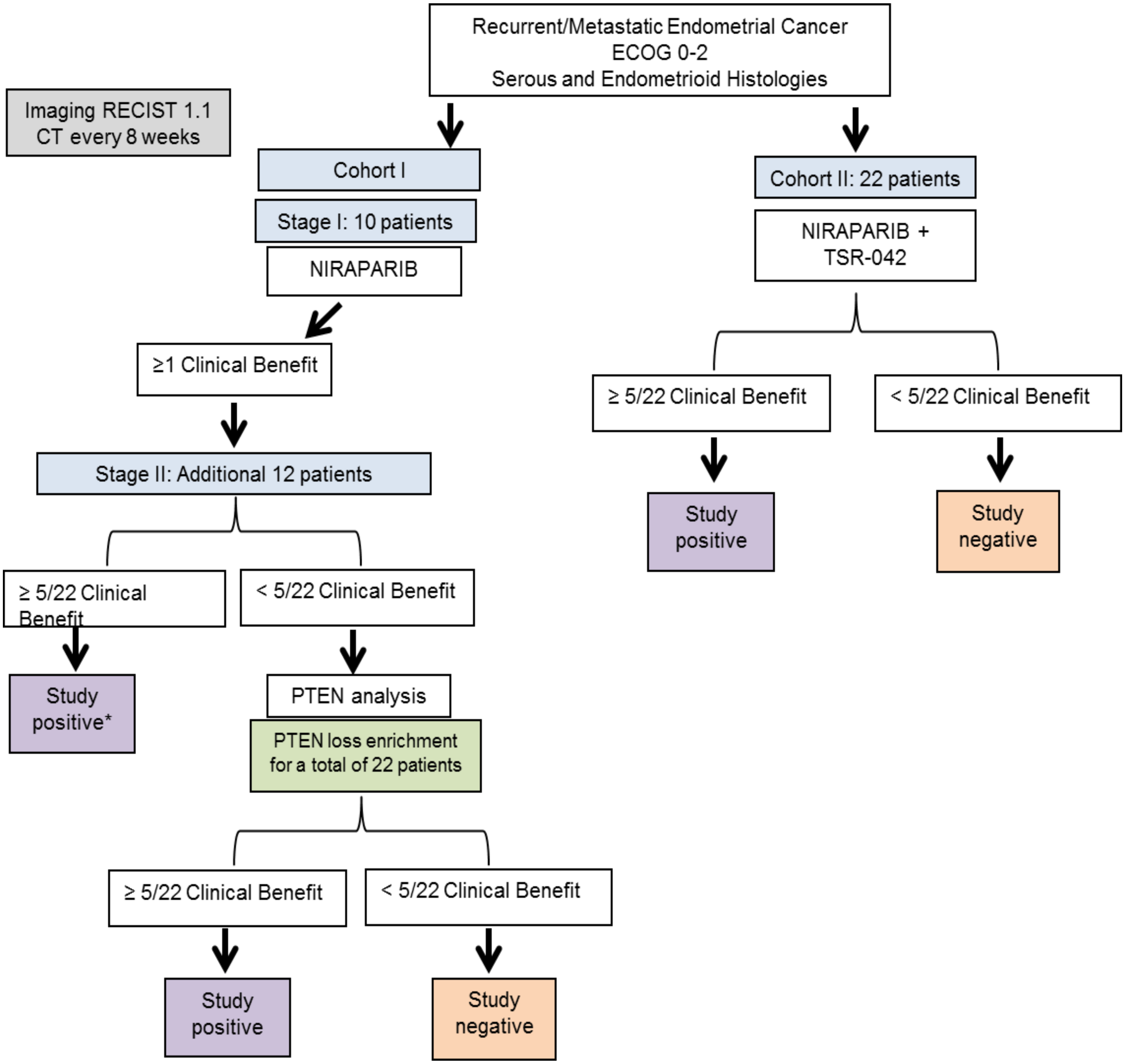

SYNOPSIS

**Title of study:** A Phase II, Open label Study of Poly(ADP-ribose) Polymerase Inhibitor Niraparib in monotherapy or in combination with anti-PD1 inhibitor TSR-042 in Recurrent Endometrial Cancer
**Objectives**:<br><br><u>Primary Objectives</u><br>• To determine the antitumor activity of single agent niraparib and of niraparib in combination with TSR-042 in women with metastatic endometrial cancer who previously received platinum-based chemotherapy, via assessment of clinical benefit rate (complete response, partial response or stable disease ≥16 weeks) according to RECIST v 1.1.<br><br><u>Secondary Objectives</u><br>• To assess the safety and tolerability of single agent niraparib and the combination of niraparib and TSR-042<br>• To determine overall response rate, duration of response, progression free survival and overall survival of niraparib and the combination of niraparib and TSR-042<br><br><u>Exploratory/Translational Objectives</u><br>• To assess if phosphatase and tensin homolog (PTEN) loss as assessed by immunohistochemistry (IHC) in archival tumor is predictive of response to treatment<br>• To assess if microsatellite instability (MSI) in archival tumor is predictive of response to treatment<br>• To assess the correlation of homologous recombination defect (HRD) status and response to treatment<br>• To correlate HRD with PTEN and MSI status respectively<br>• To assess genomic molecular analysis and response to treatment<br>• To assess the immune infiltration by PD-L1, CD3, CD8, CD20, CD21.
**Study Design:**<br>The purpose of this trial is to elucidate whether the PARP inhibition approach with niraparib, or the combination of niraparib and TSR-042, provides clinical benefit in patients with recurrent endometrial cancer.<br>The trial is designed as a multicenter, open-label, phase II study of niraparib in monotherapy or in combination with anti-PD1 inhibitor TSR-042 in recurrent endometrial cancer. Patients must have received prior platinum based chemotherapy.<br><br>The study will initially enroll patients with recurrent endometrial cancer to the niraparib monotherapy cohort not selected according to the PTEN status (cohort I).<br>Once the initial assessment with niraparib monotherapy is completed (inclusion of 22 evaluable patients), patients will be enrolled in the combination arm with niraparib and TSR-042 (cohort II).<br>If patients do not meet the pre-specified criteria for clinical efficacy, retrospective

analysis of PTEN status in cohort I will be performed while we enroll patients in cohort II and consider further enrichment of PTEN loss. Results will be available prior to end of cohort II. If the archival tissue was inadequate for PTEN analysis, patient might be replaced for the total number of patients required in cohort I. The study may continue on the selected population of patients with PTEN-loss on IHC (archival tissue or tumor biopsy).

Number of patients:
The minimal accrual in cohort I, stage I and II is 22 patients; 10 patients for stage I and 12 patients for stage II.
After 22 patients are enrolled in cohort I, additional 22 patients will be enrolled in cohort II, assessing the combination of niraparib and TSR-042.

The cohort may be considered for expansion up to 44 patients after completion of enrollment of initial 22 patients in cohort I.

Main criteria for Inclusion/Exclusion (refer section 3.1 & 3.2 for details)

<u>Inclusion Criteria</u>
1. Histologically confirmed epithelial endometrial cancer. All histological subtypes are allowed except for endometrial sarcoma, carcinosarcoma, clear cell, mixed and adenosquamous tumors.
2. Patients must have radiographic evidence of disease progression following the most recent line of treatment.
3. Patients must have previously received at least one line of platinum-based chemotherapy. Prior hormonal and immunotherapy are allowed. There is no restriction on the total number prior lines of therapy.
4. Patients must have measurable disease, defined as at least one lesion that can be accurately measured in at least one dimension (longest diameter to be recorded for non-nodal lesions and short axis for nodal lesions) as ≥10 mm with CT scan, MRI, or calipers by clinical exam, and ≥15mm for nodal lesions. Areas of previous radiation may not serve as measurable disease unless there is evidence of progression post radiation.
5. Patients must have archival tumor sample available for PTEN analysis. If archival tissue is not available, the patient will have the option to undergo tumor biopsy.
6. Eastern Cooperative Group (ECOG) performance status ≤ 2.
7. Life expectancy of greater than 12 weeks.
8. Within 7 days of the proposed start of treatment, patients must have normal organ and marrow function as defined below:

Hemoglobin	≥ 90 g/L
Leukocytes (WBC)	≥ 3.0×10^9/L
Absolute neutrophil count	≥ 1.5×10^9/L
Platelets	≥ 100×10^9/L
Total bilirubin	≤ 1.5 × institutional upper limit of normal (ULN)
AST(SGOT)/ALT(SGPT)	≤ 3.0 × institutional upper limit of normal unless liver metastases are present, in which case they must be ≤ 5 x ULN
Creatinine	≤ 1.5 × institutional upper limit of normal OR
Calculated creatinine clearance	≥ 60 mL/min using Cockcroft-Gault equation

Serum albumin ≥ 28g/L

9. Women must agree to not donate blood during the study or for 90 days after the last dose of study treatment, in cohort II.

Additional inclusion for Cohort II only:

10. International normalized ratio (INR) or prothrombin time (PT) ≤ 1.5 x ULN
Activated partial thromboplastin time (aPTT) ≤ 1.5 x ULN

11. Participant receiving corticosteroids may continue as long as their dose is stable for at least 4 weeks prior to initiating protocol therapy

12. Participant must agree not to breastfeed during the study or for 150 days after the last dose of study treatment.

Exclusion Criteria

1. Chemotherapy or biologic agents received within 4 weeks of starting study treatment.
2. Hormonal therapy within 2 weeks of starting study treatment.
3. Pelvic radiotherapy (as treatment of primary disease) within 4 weeks, or palliative radiotherapy encompassing >20% of the bone marrow within 1 week of starting study treatment.
4. Previous treatment with a PARP inhibitor, or any other targeted therapy directed against the homologous recombination pathway.
5. Patients who are receiving any other investigational agents.
6. Ongoing ≥ Grade 2 toxicities related to prior cancer therapy, with the exceptions of alopecia, neuropathy, lymphopenia and skin depigmentation.
7. Received transfusion (platelets or red blood cells) ≤4 weeks of the first dose of study treatment.
8. Major surgery within 4 weeks of registration or ongoing clinically significant post-surgical complications. Study biopsy is not considered major surgery.
9. Known brain metastases, except if stable for greater than 28 days following definitive treatment. The patient must have no new or progressive signs or symptoms related to the CNS disease and must be either off or taking a stable dose of corticosteroids. A scan to confirm the absence of brain metastases is not required.
10. History of myelodysplastic syndrome (MDS)/acute myeloid leukemia (AML).
11. History of bowel obstruction within 3 months, or other reason preventing effective oral administration of medication.
12. Known history of human immunodeficiency virus (type 1 or 2 antibodies).
13. Uncontrolled inter-current illness.
14. History of other malignancy ≤ 3 years prior to registration with the exceptions of a) cone-biopsied in situ carcinoma of the cervix uteri; b) basal or squamous cell carcinoma of the skin. All second malignancies in this context should be discussed with the PI.

Additional exclusion for Cohort II only:

15. Previous treatment with anti PD-1, anti PD-L1, antiPDL2, anti CTLA4 agents
History of fistula, or high-risk of developing a fistula.
16. Diagnosis of immunodeficiency or systemic steroids therapy or other form of immunosuppressive therapy within 7 days prior to initiating the protocol therapy.
17. Known active hepatitis (e.g., hepatitis B surface antigen [HBsAg] reactive) or

hepatitis C (e.g., hepatitis C virus [HCV] ribonucleic acid [qualitative] is detected).<br>Active autoimmune disease that has required systemic treatment in the past 2 years (ie, with use of disease-modifying agents, corticosteroids, or immunosuppressive drugs). Replacement therapy (eg, thyroxine, insulin, or physiologic corticosteroid replacement therapy for adrenal or pituitary insufficiency, etc.) is not considered a form of systemic treatment.19. History of interstitial lung disease for cohort II.<br>18. Received a live vaccine within 14 days of initiating protocol therapy
**Intervention**:<br>In the absence of treatment delays due to adverse event(s), treatment may continue until one of the following criteria applies:<br>• Any treatment-related Common Terminology Criteria for Adverse Events (CTCAE) Grade 3 or 4 events that have not reverted to baseline or CTCAE Grade 1 or better within 28 days. At the Investigator's discretion, following dose interruption (no longer than 28 days), patients may be considered for dose reductions provided they have not already undergone the maximum allowed number of 2 dose reductions. If upon re-challenging with study treatment at the lowest allowable dose, any CTCAE Grade 3 or 4 adverse events recur, the patient should stop treatment<br>• If platelet count has not reverted to $\geq 100x10^9/L$ within 28 days of treatment interruption.<br>• Disease progression as defined by section 11<br>• Clinical progression<br>• Diagnosis of MDS or AML (as confirmed by a hematologist)<br>• Patients requiring more than 2 dose reductions of niraparib.<br>• Delays in TSR-042 treatment will be allowed for 4 weeks, if there is benefit in restarting the treatment, it will be discussed with the PI. TSR-042 can be continued for a maximum of 2 years.<br>• Patient decides to withdraw from the study, or<br>• General or specific changes in the patient's condition render the patient unacceptable for further treatment in the judgment of the investigator.
**Correlatives:**<br>• To assess whether the presence PTEN deficiency in archival tumor is predictive of clinical benefit and response to treatment<br>• To explore whether the presence of MSI status is predictive of clinical benefit and response rate to treatment<br>• To correlate HRD with PTEN and MSI status respectively.<br>• To explore whether HRD positivity in endometrial cancer is predictive of clinical benefit and response rate to treatment<br>• To assess the immune infiltration by PD-L1, CD3, CD8, CD20, CD21

Statistics:

The Simon two-stage design is employed. With the null hypothesis that Clinical benefit rate (CBR), $p \leq 0.10$ versus the alternative that $p \geq 0.35$ and setting alpha=beta=0.10, Stage I has a planned accrual of 10 patients. If at least 1 clinical benefit instance is observed at the end of stage I, the study will proceed to stage II with 12 additional patients to be accrued for the total of 22 patients. If at least 5 instance of clinical benefit are observed among the 22 patients, this agent would be considered worthy of further investigation.

The study will enroll patients with recurrent endometrial cancer unselected for PTEN status. Mandatory archival tissue is requested for retrospective analysis.

After the enrollment of a total of 22 patients in cohort I (niraparib alone), new patients will be registered in cohort II with the combination of niraparib and TSR-042.

If at least 5 instance of clinical benefit are observed among the 22 patients in cohort II, this agent would be considered worthy of further investigation.

If the clinical benefit rate does not reach the pre-defined level (positive ≥5/22 overall) after stage II in cohort I, PTEN analysis will be performed and the study will be considered to expand to PTEN-loss subgroup if this subgroup's CBR is not worse than the rest of the patients initially in cohort 1 with niraparib single agent. The same criteria as calculated by the design above (≥1/10 CBR go to stage II and positive ≥5/22 CBR overall) will be used.

LIST OF ABBREVIATIONS AND DEFINITIONS OF TERMS

PARP	Poly(ADP-ribose) polymerases
DNA	Deoxyribonucleic acid
BER	Base-excision-repair
NHEJ	Non-homologous end joining
gBRCA	germline BRCA
gBRCAmut	germline BRCA mutated
HRD	Homologous Recombination Deficiency
HGS-OvCa	High Grade Serous Ovarian Cancer
PFS	Progression Free Survival
HR	Hazard Ratio
PROs	Patient Reported Outcomes
FOSI	Functional Assessment of Cancer Therapy – Ovarian Symptoms Index
MDS	Myelodysplastic Syndrome
AML	Acute Myeloid Leukemia
TCGA	The Cancer Genome Atlas
MMR	Mismatch Repair
MSI	Microsatellite Instability
PDX	Patient-derived Xenograft
IC50	Half Maximal Inhibitory Concentration
shRNA	Short Hairpin Ribonucleic Acid
CC	Cytotoxic Concentration
QD	Once per day
BID	Twice per day
NGS	Next Generation Sequencing
RP2D	Recommended Phase 2 Dose
MTD	Maximum Tolerated Dose
DLT	Dose Limiting Toxicity
TMZ	Temozolomide
GBM	Glioblastoma Multiforme
RECIST	Response Evaluation Criteria In Solid Tumors
NCI	National Cancer Institute
AE	Adverse Events
CTCAE	Common Terminology Criteria for Adverse Events
CI	Confidence Interval
SD	Stable Disease
IHC	Immunohistochemistry
CT	Computed Tomography
MRI	Magnetic Resonance Imaging
CBC	Complete Blood Count
PD	Progressive disease
PK	Pharmacokinetics
CSR	Clinical Study Report

TABLE OF CONTENTS

SCHEMA....................17

SYNOPSIS....................4
Primary Objectives....................18
Secondary Objectives....................18
Exploratory/Translational Objectives....................18

List of Abbreviations and Definitions of Terms....................23

1. OBJECTIVES....................26
1.1 Primary Objectives....................26
1.2 Secondary Objectives....................26
1.3 Exploratory Objectives....................26

2. BACKGROUND....................27
2.1 Disease Background....................27
2.2 Niraparib....................30
2.4 Background TSR-042....................23
2.5 TSR and Niraparib combination....................24
2.6 Rationale....................39
2.7 Correlative Studies Background....................41

3. PATIENT SELECTION....................43
3.1 Eligibility Criteria....................43
3.2 Exclusion Criteria....................45
3.3 Inclusion of Women and Minorities....................47

4. REGISTRATION PROCEDURES....................48
4.1 General Guidelines....................48
4.2 Registration Process....................48

5. TREATMENT PLAN....................49
5.1 Agent Administration....................49
5.2 General Concomitant Medication and Supportive Care Guidelines....................51
5.3 Duration of Therapy....................52
5.4 Duration of Follow Up....................54
5.5 Criteria for Removal from Study....................40

6. DOSING DELAYS/DOSE MODIFICATIONS....................54
6.1 General Guidelines....................541
6.2 General guidelines: TSR-042....................44

7. ADVERSE EVENTS: LIST AND REPORTING REQUIREMENTS....................63
7.1 List of Adverse Events and Reporting Requirements....................63
7.2 Adverse Event Characteristics....................65

7.3 Serious Adverse Event Reporting 67
7.4 Routine Adverse Event Reporting 68
7.5 Documentation of Adverse Events 69
7.6 Follow-Up of AEs and SAEs 69
7.7 Pregnancy 69
7.8 Investigator Notifications/Safety Notifications/Safety Reports 70
7.9 Reporting Product Quality Complaints for Niraparib 70
7.10 Data Safety and Monitoring Board 70

8. PHARMACEUTICAL INFORMATION 71
8.1 Investigational Agent 71
8.2 TSR-042 58

9. BIOMARKER, CORRELATIVE, AND SPECIAL STUDIES 73
9.1 Biomarker Studies 73
9.2 Laboratory Correlative Studies 73

10. STUDY CALENDAR 77

11. MEASUREMENT OF EFFECT 81
11.1 Antitumor Effect – Solid Tumors 81

12. DATA REPORTING / REGULATORY REQUIREMENTS 85
12.1 Data Collection and Reporting 85
12.2 Source Documents 70
12.3 Retention of Patient Records and Study Files 861
12.4 Site and Study Closure 86

13. STATISTICAL CONSIDERATIONS 883
13.1 Study Design/Endpoints 883
13.2 Sample Size/Accrual Rate 883
13.3 Analysis of Secondary and Exploratory Endpoints 883

REFERENCES 905

APPENDIX 1 PERFORMANCE STATUS CRITERIA 961

APPENDIX 2 DATA MANAGEMENT GUIDELINES 972

APPENDIX 3 WHO MDS CLASSIFICATION CRITERIA 99

1. OBJECTIVES

Targeting the homologous recombination (HRD) pathway has emerged as an attractive therapeutic strategy in endometrial cancer. Thus, the purpose of this trial is to elucidate whether the PARP inhibition approach with niraparib, or the combination of niraparib and TSR-042, provides clinical benefit in patients with recurrent endometrial cancer. This study will recruit patients with recurrent endometrial cancer previously treated with platinum based chemotherapy.

1.1 Primary Objectives

- To determine the antitumor activity of single agent niraparib and of niraparib in combination with TSR-042 in women with metastatic endometrial cancer who has received prior platinum-based chemotherapy via assessment of clinical benefit rate (complete response, partial response or stable disease ≥16 weeks), according to RECIST v 1.1.

1.2 Secondary Objectives

- To assess the safety and tolerability of single agent niraparib and of niraparib in combination with TSR-042.
- To determine overall response rate, duration of response, progression free survival and overall survival of niraparib and the combination of niraparib and TSR-042

1.3 Exploratory Objectives

- To assess if PTEN loss as assessed by immunohistochemistry (IHC) in archival tumor is predictive of response to treatment
- To assess if MSI in archival tumor is predictive of response to treatment
- To assess the correlation of HRD status and response to treatment
- To correlate HRD with PTEN and MSI status respectively
- To assess genomic molecular analysis and response to treatment
- To assess the immune infiltration by PD-L1, CD3, CD8, CD20, CD21.

2. BACKGROUND

2.1 Disease Background

Endometrial cancer is the highest incidence gynecologic malignancy and remains the fourth most common cancer diagnosis in North American women [1]. In 2012, there were approximately 5,600 new cases of endometrial cancer in Canada, from whom it is estimated that 900 women will die of their disease [2].

The main risk factor for the development of endometrial cancer continues to be prolonged exposure to unopposed estrogen [3]. Other identified risk factors (nulliparity, use of tamoxifen, hormone replacement therapy etc) are likely relevant due to the pathology-promoting imbalance of estrogen/progesterone [4]. The ever growing obesity epidemic with its consequent metabolic syndromes is yet another factor promoting estrogenic imbalances in women and is likely a major contributing factor to the burden of disease we currently see related to endometrial cancer [5].

Endometrial cancer is predominantly a disease of older women who present with aberrant post-menopausal bleeding as the first symptom of their disease [6]. The majority of women are diagnosed with early stage disease and have a relatively good prognosis, with 5-year disease-free survivals of greater than 80% [7]. Surgery continues to be the mainstay of management; however, chemotherapy and radiation have demonstrated utility at reducing the risk of local and distant disease recurrence and have now become standard practice [8]. However, a significant percentage of women with early stage disease are at a greater risk of developing disease recurrence. Factors like age > 60, depth of invasion, involvement of lower uterine segment, non-endometroid histology and the presence of lymphovascular invasion or aneuploidy, have demonstrated importance in identifying those at a particularly high risk of failing primary therapy [8]. Women with more advanced disease at initial presentation have a relatively poor prognosis with a 5-year disease free survival of about 20% [7].

There is significant heterogeneity within this group, as patients with endometroid histology tend to respond to hormonal manipulation while patients with high grade serous cancers follow a more aggressive course and benefit from a limited number of cytotoxic therapies. The best responses are to agents like doxorubicin and cisplatin, although these responses remain modest and in the range of ~ 20 to 40% and where noted are transient in duration [9, 10]. Although combination regimens have demonstrated higher response rates, the associated increased toxicities and unclear impact on survival makes their role in the palliative setting uncertain.

2.1.1 <u>Relevance of Histopathology and Molecular Characterization</u>

In the past 30 years, endometrial cancer has been broadly classified into two subtypes on the basis of histological characteristics, hormone receptor expression, and grade [11]. The majority of patients are diagnosed with endometroid adenocarcinomas known as Type I cancers, which are characteristically low-grade, on a background of atypical complex hyperplasia [11]. They express hormone-receptors and are usually associated with a good prognosis. Type II endometrial cancers are described as non-endometrioid, high grade, aneuploid, TP53-mutated, hormone-receptor negative tumors that are associated with a higher risk of metastasis and a poor prognosis [11]. This

group also includes clear cell, carcinosarcoma and stromal sarcomas albeit at much lower frequencies all with their own unique clinical and molecular profiles [11]. Comprising a small proportion of all uterine cancers, non-endometroid histology cancers account for about 50% cases of recurrent disease and therefore represent a significant management challenge [6]. Whilst this dualistic classification has begun being incorporated into clinical decision-making algorithms defining high-risk patients, its prognostic value remains limited given 20% of endometrioid (i.e. type I) endometrial cancers relapse; whereas 50% of non-endometrioid (i.e., type II) endometrial cancers do not[6]. Additionally, 15–20% of endometrioid tumors are high-grade lesions, and where they fit into the dualistic model is unclear [12, 13].

Endometrial cancer comprises a range of diseases with distinct genetic and molecular features. Analyses by The Cancer Genome Atlas (TCGA) focusing on endometrioid and serous endometrial cancer further emphasize the disease's heterogeneity [14]. Within type I endometrial cancer, the PIK3/AKT/mTOR pathway is the commonest genetic aberration, with mutations noted in more than 90% of tumors. Furthermore, molecular alterations in the negative regulator of this pathway, PTEN, are common- including PTEN mutations (77%) or loss of PTEN heterozygosity (40%). PIK3CA mutations (42-53%). KRAS mutations (25% of tumors), CTNNB1 (37%), ARID5B (35%), and FGFR2 mutations (12%) are also frequently seen [14, 15].

Type II endometrial cancers include a range of histological subtypes, each showing distinct molecular and genomic features. The serous subtype appeared to have a similar genetic background to serous ovarian and breast cancers, including hallmarks of deficiency in DNA repair and frequent TP53 (91%), PIK3CA (41.9%), FBXW7 (30.2%) and PPP2R1A (27.9%) mutations [14, 16]. Clear-cell endometrial cancer resembles its ovarian clear-cell counterpart, with inactivating mutations in the chromatin remodeling gene ARID1A in 20–40% of cases and universal expression of hepatocyte nuclear factor-1β [17-19]. Interestingly, a substantial overlap in genetic defects occurs within endometrial cancer subtypes. PIK3CA mutations, commonly presented in type I tumors, are also present in up 40% serous endometrial cancer, and the presence of concomitant molecular deficiencies have also been described, showing the heterogeneity of this disease [14].

TCGA investigations identified four distinct molecular subgroups: POLE ultramutated, microsatellite instability (MSI) hyper mutated, copy-number-low microsatellite stable, and copy-number-high serous-like, showing increasing grade, TP53, and high somatic copy number alterations, but decreased mutation rates [14]. The newly identified POLE ultramutated category is the smallest subgroup, but defines a unique subset characterized by mutations in the exonuclease domain of POLE, high mutation load, and an excellent prognosis [20]. Approximately 60% of POLE ultramutated endometrial cancers are high-grade endometrioid lesions, and 35% harbor TP53 mutations. Roughly 30–40% of endometrioid endometrial cancers show loss of DNA mismatch repair (MMR) proteins (MLH1, MSH2, MSH6, and PMS2); in sporadic cases this is secondary to MLH1 promoter hypermethylation, and in hereditary Lynch syndrome it can be caused by mutations in any of the DNA MMR genes [21, 22]. The microsatellite stable subgroup is characterized by low mutation load, a low rate of somatic copy number alterations, and intermediate prognosis [14]. The copy-number high subgroup includes most serous endometrial cancers and 25% of the high-grade endometrioid cancers that display genomic instability, with frequent somatic copy number alterations and poor prognosis [14]. High-grade endometrioid

endometrial cancers are heterogeneous—25% are copy-number-high serous endometrial cancers with poor prognosis; another quarter are ultramutated POLE cancers, which have good prognosis [14]. Given the inherent heterogeneity in this patient population with respect to both clinical course and therapeutic response, there is optimism that novel approaches aimed specifically at differential vulnerabilities within different molecular subgroups of this disease will improve outcomes for patients with endometrial cancer.

2.2 BACKGROUND Immune Surveillance and PD-1 Inhibitors

The importance of intact immune surveillance in controlling outgrowth of neoplastic transformation has been known for decades. Accumulating evidence shows a correlation between tumor-infiltrating lymphocytes in cancer tissue and prognosis in various malignancies.[23-35] In particular, the presence of cluster of differentiation (CD)8+ T cells and the ratio of CD8+ effector T cells/FoxP3+ regulatory T cells seem to correlate with improved prognosis and long-term survival in many solid tumors.[31, 36-42] The programmed death-1 (PD-1) receptor-ligand interaction is a major pathway hijacked by tumors to suppress immune control.[43] The normal function of PD-1, expressed on the cell surface of activated T cells under healthy conditions, is to down-modulate unwanted or excessive immune responses, including autoimmune reactions. PD-1 (encoded by the gene Pdcd1) is an immunoglobulin (Ig) superfamily member related to CD28 and cytotoxic T-lymphocyte-associated protein 4 (CTLA-4), which has been shown to negatively regulate antigen receptor signaling upon engagement of its ligands (programmed death-ligand 1 [PD-L1] and programmed death-ligand 2 [PD-L2]). The structures of murine PD-1 alone[44] and in complex with its ligands were the first to be resolved,[45, 46] and more recently the nuclear magnetic resonance-based structure of the human PD-1 extracellular region and analyses of its interactions with its ligands were also reported.[47] PD-1 and family members are Type I transmembrane glycoproteins containing an Ig variable-type (V-type) domain responsible for ligand binding and a cytoplasmic tail, which is responsible for the binding of signaling molecules. The cytoplasmic tail of PD-1 contains 2 tyrosine-based signaling motifs, an immunoreceptor tyrosine-based inhibition motif, and an immunoreceptor tyrosine-based switch motif (ITSM). Following T cell stimulation, PD-1 recruits the tyrosine phosphatases SHP-1 and SHP-2 to the ITSM within its cytoplasmic tail, leading to the dephosphorylation of effector molecules, such as CD3ζ, PKCθ, and ZAP70, which are involved in the CD3 T cell signaling cascade.[48] The mechanism by which PD-1 down-modulates T cell responses is similar to, but distinct from, that of CTLA-4.[49] PD-1 was shown to be expressed on activated lymphocytes, including peripheral CD4+ and CD8+ T cells, B cells, T regs, and natural killer cells.[50] Expression has also been shown during thymic development on CD4-/CD8- (double-negative) T cells,[51] as well as subsets of macrophages[52] and dendritic cells.[53] The ligands for PD-1 (PD-L1 and PD-L2) are constitutively expressed or can be induced in a variety of cell types.[54] PD-L1 is expressed at low levels on various nonhematopoietic tissues, most notably on vascular endothelium, whereas PD-L2 protein is predominantly expressed on antigen-presenting cells found in lymphoid tissue or chronic inflammatory environments.[54] Both ligands are Type I transmembrane receptors containing both IgV- and IgC-like domains in the extracellular region and short cytoplasmic regions with no known signaling motifs. Binding of either PD-1 ligand to PD-1 inhibits T cell activation triggered through the T cell receptor. PD-L2 is thought to control immune T cell activation in lymphoid organs, whereas PD-L1 serves to dampen unwarranted T cell function in

peripheral tissues. Although healthy organs express little (if any) PD-L1, a variety of cancers were demonstrated to express abundant levels of this T cell inhibitor,[55, 56] which, via its interaction with the PD-1 receptor on tumor-specific T cells, plays a critical role in immune evasion by tumors.[57] As a consequence, the PD-1/PD-L1 pathway is an attractive target for therapeutic intervention in cancer.[58]

Immune surveillance in endometrial cancer

Two subgroups of endometrial cancer, POLE-ultra-mutated and MSI-H, are characterized by higher number of neo-antigens and the elevated amount of TILs [59].

In 2015 Le et al. published a phase II trial leading to the approval of Pembrolizumab, an anti–programmed death 1 (anti-PD1) immune checkpoint inhibitor, in MSI solid tumours. Study population was divided in three cohorts, including patients with MMR-deficient colorectal cancer, MMR proficient colorectal cancer and MMR-deficient solid tumours (non-colorectal). The third cohort included two patients with endometrial carcinoma, showing immune-related objective response rate (ORR) and PFS of 71% and 67%, respectively. Moreover, there was a higher immune-related ORR and 20-week immune related progression free survival (PFS), 40% and 78%, respectively, in the MMR deficiency cohorts, versus 0% and 11% in MMR proficient colorectal patients [60]. On an update of the study, ORR was observed in 53% of patients and complete responses were achieved in 21% of patients [61]. Long responses have also been described in case series of patients with POLE mutated and MSI endometrial tumours with other anti-PD1 treatments [62].

Similarly, other early studies have been done including women with endometrial cancer without MSI or POLE molecular screening. On KEYNOTE-028 is a phase Ib study in patients with programmed death ligand 1 (PD-L1) –positive advanced solid tumors progressing to standard treatment, of whom 24 had endometrial tumours and 2 where SC. ORR was 13%, three patients obtained a partial response and other three stable disease. Six-months PFS and OS rates were 19.0% and 68.8% respectively [63].

Combination trials with anti-PD1/PDL1 have also been published. A phase I / II trial with 23 women treated with Lenvatinib and Pembrolizumab showed an ORR of 48% and a disease-control rate of 96%. The most common adverse events were hypertension, fatigue, arthralgia, diarrhea and nausea [64].

2.3 Niraparib

2.3.1 Background of PARP and PARP Inhibition

Poly(ADP-ribose) polymerases (PARP-1 and -2) are zinc-finger deoxyribonucleic acid (DNA)-binding enzymes that play a crucial role in DNA repair [65]. Upon formation of single-strand DNA breaks, PARP binds at the end of broken DNA strands, thus activating its enzymatic activity. Activated PARP catalyzes the addition of long polymers of ADP-ribose on several proteins associated with chromatin, including histones and various DNA repair proteins including PARP itself [66-68]. This results in chromatin relaxation, fast recruitment of DNA repair proteins and efficient repair of DNA breaks. In this manner, PARP plays a central role in sensing DNA damage

and converting it into intracellular signals that activate the base-excision-repair (BER) and single strand break repair pathways [66-68].

Normal cells repair up to 10,000 DNA defects daily and single strand breaks are the most common form of DNA damage. Cells unable to repair this burden of DNA damage—such as those with defects in the BER pathway, or those treated with PARP inhibitors—are at risk for accumulating multiple lesions that will ultimately trigger apoptosis as they progress to enter the S (DNA replication) phase of the cell cycle with unrepaired single strand breaks [69]. Pre-existing single strand breaks are converted to double strand breaks as the replication machinery passes and those accumulated double strand breaks present during S phase are then repaired by homologous recombination [69]. Homologous recombination is the preferred repair pathway because it is associated with a much lower error rate than other forms of repair. Cells unable to perform DNA repair via homologous recombination (e.g., due to inactivation of genes required for homologous recombination, such as BRCA-1 or BRCA-2) are at risk for accumulating multiple lesions that will ultimately trigger apoptosis [69]. Specifically, these cells accumulate stalled replication forks during S phase and are more likely to use the error-prone non-homologous end joining (NHEJ) pathway to repair double strand breaks in DNA [70]. It is the accumulation of errors in DNA by NHEJ that contributes to mutations promoting the development of cancer. Over time, the buildup of excessive DNA errors in combination with the inability to complete S phase (because of stalled replication forks) contributes to cell death [70].

A hypothesis is that treatment with PARP inhibitors represents a novel opportunity to selectively kill a subset of cancer cells with deficiencies in DNA repair pathways. For example, a tumor arising in a patient with a *BRCA1/2* mutation (*BRCA*mut) has a defective homologous recombination DNA repair pathway and would be increasingly dependent on BER (a pathway blocked by PARP inhibitors) for maintenance of genomic integrity [69]. Non-*BRCA* deficiencies in homologous recombination DNA repair genes could also enhance tumor cell sensitivity to PARP inhibitors. The rationale for anticancer activity in a subset of non-*BRCA*mut tumors is they share distinctive DNA-repair defects with g*BRCA*mut carriers, a phenomenon broadly described as “BRCAness” [71]. DNA-repair defects can be caused by germline or somatic alterations to the homologous recombination DNA repair pathway. In an analysis of ~500 high grade serous ovarian cancer, approximately 50% contained HR defects [72]. These tumors had biologically plausible molecular alterations that may make them sensitive to PARP inhibition, such as with niraparib. This concept of inducing death by use of PARP inhibitors to block one DNA repair pathway in tumors with pre-existing deficiencies in a complementary DNA repair pathways is called synthetic lethality [73], the two insults together induce cell death when neither alone would have this effect.

Clinical studies have shown that PARP inhibitors are active for recurrent ovarian cancer [74-77]. Clinical anticancer activity has been observed in patients with and without g*BRCA*mut and in patients who are platinum sensitive and platinum-resistant; however, PARP inhibition appears to be most active in patients with *BRCA* mutated disease [74, 75, 77, 78]. This clinical experience to date is largely in patients who have received multiple prior treatments (median 3, range 1-10) in multiple early phase clinical studies. In a phase 2 study of maintenance therapy in 265 patients with relapsed, platinum-sensitive ovarian cancer, daily olaparib therapy compared to placebo treatment was associated with a progression free survival (PFS) benefit (hazard ratio [HR]: 0.35) and prolongation of median PFS from 4.8 months to 8.4 months [77]. The subset of patients with

known *BRCA*mut had a PFS hazard ratio of 0.1. Patient reported outcomes (PROs) in this study, which included the Functional Assessment of Cancer Therapy – Ovarian Symptoms Index (FOSI), were measured and were not found to show a significant difference between the placebo and treatment groups; thus, suggesting maintenance treatment did not decrease functioning or quality of life in these patients [77].

2.3.2 Characterization of Niraparib

The chemical name for niraparib is ([3S]-3-[4-{7-(aminocarbonyl)-2H-indazol-2-yl} phenyl] piperidine [tosylate monohydrate salt]). The current clinical formulation of niraparib is a dry-filled capsule available in 100 mg.

Niraparib is an orally selective PARP1/2 inhibitor that selectively kills *in vitro* tumor cells with inactivation of *BRCA1*, *BRCA2* or *ATM* genes. In the nonclinical setting, anti-tumor activity was demonstrated in studies with *BRCA1*-mutant and *BRCA2*-mutant mouse xenograft models. In *BRCA1* mutant xenograft studies, niraparib dosed orally caused tumor regression, which was mirrored by >90% reduction in tumor weight compared with the control. In *BRCA2*-mutant xenograft studies, niraparib-dosed mice showed 55% - 60% growth inhibition (by both tumor volume and weight). Nonclinical efficacy has also been demonstrated in breast and ovarian patient-derived xenograft (PDX) models that show evidence of HRD.

Results from clinical testing in Phase 1 indicated that niraparib is generally safe and well tolerated in patients with advanced solid tumors. Preliminary safety data from two Phase 3 studies (including one blinded, placebo-controlled study) also suggest that treatment with niraparib is generally well tolerated. Preclinical data on niraparib in ovarian cancer are described in the Investigator's Brochure. Niraparib clinical safety data, including Phase 1 results, dose limiting toxicities and toxicity profile are provided in detail in the Investigator's Brochure.

2.3.2.1 Chemical Structure

The chemical structure of niraparib is shown in Figure 1.

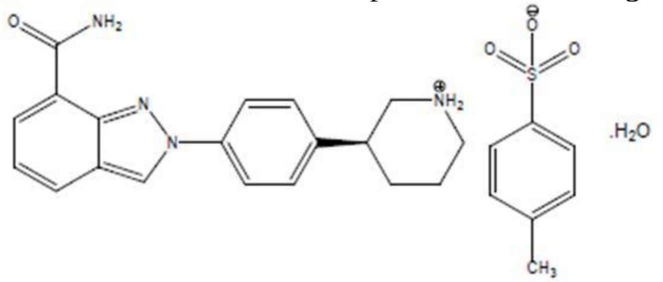

Figure 1: Chemical Structure of Niraparib

2.3.2.2 Non-clinical Development

Niraparib is an orally active PARP1/2 inhibitor with nanomolar potency being developed as a monotherapy agent for tumors with defects in the homologous recombination DNA repair pathway or that are driven by PARP-mediated transcription factors. In preclinical models, niraparib has

been observed to inhibit normal DNA repair mechanisms and induce synthetic lethality when administered to cells with homologous recombination defects. In a *BRCA1* mutant xenograft study, niraparib dosed orally caused tumor regression which was mirrored by >90% reduction in tumor weight compared to control; in a *BRCA2* mutant xenograft study, niraparib dosed mice showed 55-60% growth inhibition, both by tumor volume and weight. Preclinical data on niraparib in ovarian cancer are provided in detail in the Investigator's Brochure (IB).

2.3.2.3 Clinical Development

Overview

Niraparib has been evaluated in 144 humans in Phase 1 clinical studies. Over 500 patients (427 patients of a data cutoff date of 02 February 2015) have received niraparib or placebo in the Phase 3 clinical study program. An overview of clinical studies conducted for niraparib is presented in Table 1. Detailed information for each of these studies, including pharmacokinetic data, can be found in the Investigator's Brochure.

Table 1: Overview of Clinical Studies of Niraparib

Protocol Number	Study Title	Status
**Phase 1**		
PN001	A Phase 1 Study Of Niraparib In Patients With Advanced Solid Tumors Or Hematologic Malignancies	Completed
PN005	A Phase 1 Study Of Niraparib In Patients With Solid Tumors	Terminated
PN008	A Phase 1b Dose Escalation Study Of Niraparib In Combination With Carboplatin, Carboplatin/Paclitaxel And Carboplatin/Liposomal Doxorubicin In Patients With Advanced Solid Tumors	Terminated
PN011	A Phase 1b Dose Escalation Study Of Niraparib In Combination With Pegylated Liposomal Doxorubicin (Doxil™ Or Caelyx™) In Patients With Advanced Solid Tumors With A Cohort Expansion In Patients With Platinum-Resistant/Refractory High Grade Serous Ovarian Cancer.	Terminated
PN014	A Phase 1 Study Of Niraparib In Combination With Temozolomide (TMZ) In Patients With Advanced Cancer	Completed
PR-30-5015-C	Absorption, Metabolism, Excretion, and the Determination of Absolute Bioavailability of Niraparib in Subjects with Cancer	Ongoing
**Phase 3**		
PR-30-5010-C	A Phase III, Randomized, Open Label, Multicenter, Controlled Trial of Niraparib Versus Physician's Choice in Previously-Treated, HER2 Negative, Germline BRCA Mutation-Positive Breast Cancer Patients	Ongoing
PR-30-5011-C	A Phase 3 Randomized Double-Blind Trial of Maintenance With Niraparib Versus Placebo in Patients With Platinum Sensitive Ovarian Cancer	Ongoing

Data on file, TESARO

Phase 1 Clinical Studies

Five Phase 1 clinical studies (PN001, PN005, PN008, PN011, PN014) were conducted in 144 patients. In addition, PR-30-5015-C is enrolling patients (anticipated 12 patients), as of the time of the latest Investigational Brochure (IB). A decision was made to suspend new enrollment in Protocols PN001, PN005, PN008, and PN011. This decision was not related to any concerns regarding the safety of the product.

Niraparib Monotherapy Studies

Study PN001

The primary objective of the open-label study PN001 was to establish the safety, tolerability, PK, pharmacodynamics, and recommended Phase 2 Dose (RP2D) of niraparib in patients with advanced solid tumors. A total of 60 patients were treated in Part A QD at 10 dose levels ranging from 30 mg to 400 mg. The maximum tolerated dose (MTD) determined in Part A was 300 mg QD. The second part of the study (Part B) was a cohort expansion in patients with platinum-resistant, recurrent, high-grade serous ovarian cancer or prostate cancer at the MTD established in Part A. A total of 40 patients were treated in Part B at the MTD of 300 mg QD as determined in Part A. The third part of the study (Part D) included patients with colorectal cancer, endometrial cancer, partially platinum-sensitive high grade serous ovarian cancer, or breast cancer. A total of 4 patients were treated in Part D at the RP2D of 300 mg QD. Among the 104 patients enrolled in this study, 14 patients achieved confirmed or unconfirmed partial response by Response Evaluation Criteria In Solid Tumors (RECIST) v.1.1 and/or by CA-125 criteria. The overall response rate in this study was 14 of 104 (13%) (95% confidence interval [CI], 8%, 22%). There were 49 ovarian cancer patients in this study, including 1 patient with high-grade peritoneal carcinoma. Twenty were BRCA-mutant patients. The response rate was 7 of 20 (35%) (95% CI, 15%, 59%). Of the 10 patients with BRCA mutation and platinum-sensitive ovarian cancer, 6

(60%) responded. Among the 22 ovarian cancer patients in Part B who were not *BRCA*-mutant or who had unknown *BRCA* mutation status, including 1 patient with high-grade peritoneal carcinoma, 1 patient achieved confirmed partial response by RECIST v.1.1, and another 2 patients achieved unconfirmed CA-125 partial response. The response rate was 3 of 22 (14%) (95% CI, 3%, 35%).

Study PR-30-5015-C

PR-30-5015-C is an open-label study with 2 parts, plus an extension study following completion of Parts 1 or 2, that is being conducted in approximately 12 patients (6 patients in Part 1; 6 patients in Part 2) with cancer to examine the absorption, metabolism, excretion, and absolute bioavailability of niraparib. This study is the first-in-human administration of the IV formulation of niraparib. The study is ongoing and was enrolling patients at the time of the database cut-off date for the current IB (29 September 2015).

Niraparib Combination Studies

Study PN014

The primary objective of open-label study PN014 was to define the dose limiting toxicity (DLT) and MTD of niraparib + temozolomide (TMZ) administered to adult patients with advanced solid tumors. The secondary objective was to explore the anti-tumor activity of niraparib + TMZ in enriched subpopulations of recurrent glioblastoma multiforme (GBM) and melanoma patients. Hematological toxicity (thrombocytopenia) was observed in heavily pretreated patients enrolled at the 40 mg niraparib and 150 mg/m2 TMZ dose level. The DLT of Grade 4 thrombocytopenia occurred in 2/10 patients at the 40 mg dose level for niraparib and was not unexpected as it is considered to be an overlapping toxicity of the 2 individual agents. The DLT of Grade 4 neutropenia occurred in 1/3 patients at the 70 mg dose level for niraparib. In addition, all 3 patients at 70 mg experienced Grade 4 thrombocytopenia with only one patient meeting protocol-specified DLT criteria. Due to this observation, it was concluded that, in combination with TMZ, a dose of 40 mg QD of niraparib was the MTD and RP2D. This study was closed after defining an MTD in Part A; Part B was not conducted. Nineteen patients were enrolled (n = 6 niraparib 30 mg + TMZ, n = 10 niraparib 40 mg + TMZ, and n = 3 niraparib 70 mg + TMZ). Partial response was reported in 1 patient with GBM after 6 treatment cycles at 40 mg niraparib. Stable disease (SD) was reported in 2 patients: 1 patient with malignant melanoma, and 1 patient with serous ovarian cancer. Progressive disease was reported in the remaining 13 patients.

Phase 3 Clinical Studies

Study PR-30-5011-C

The main study of PR-30-5011-C is a double-blind, 2:1 randomized, placebo controlled study in platinum-sensitive ovarian cancer patients who have either *BRCA*mut or a tumor with high grade serous histology. The patients must have received at least 2 platinum-based regimens, had a response to their last regimen, and have no measurable disease >2cm and normal CA-125 (or >90% decrease) following their last treatment. The study assessed whether maintenance with niraparib will extend progression free survival (PFS) in this population. There are 2 independent patient cohorts in this study, one cohort of patients with deleterious *BRCA* mutations and the other composed of patients with high-grade serous histology but without such *BRCA* mutations (non-*BRCA*mut) based on the hypothesis that patients with *BRCA* mutations will be enriched for responsiveness to niraparib. The results of this study has now been announced in a press release

(29 June 2016) positive results. In the *BRCA*mut subgroup there was statistically significant PFS benefit from median PFS of 21 months in patients treated with niraparib, versus 5.5 months in control, hazard ratio 0.27 (p< 0.0001). There was also significant PFS improvement in the non-*BRCA*mut who are HRD positive as determined on the Myriad myChoice HRD test. The niraparib arm had a median PFS of 12.9 months, compared to 3.8 months in control; HR 0.38 (p<0.0001). Finally, in the overall non-*BRCA*mut population (with both HRD-positive and HRD-negative tumors) niraparib treatment provided statistically significant PFS benefit from median 3.9 months in control to 9.3 months in niraparib group, HR 0.45 (p<0.0001). PR-30-5011-C also contains a 14-day, open-label, 2-treatment, crossover sub-study to evaluate the effect of a high fat meal on niraparib (single dose) exposure.

2.3.2.4 Safety

Safety in the Phase 1 Patient Population (Overall)

Several of the Phase 1 clinical studies were terminated for reasons that did not have to do with safety. A total of 144 patients have received treatment with niraparib as monotherapy or in combination with chemotherapy in the Phase 1 study program (Protocols PN001, PN005, PN008, PN011, and PN014). Detailed information for each of these studies, including safety data, can be found in the IB.

Safety in Phase 3 Studies

Study PR-30-5011-C

The main phase III study (PR-30-5011-C) has been completed but awaiting full presentation and publication of results. As report in the press release of 29 June 2016, most common (occurred in at least 10% of patients) grade 3/4 adverse events in niraparib treated patients were thrombocytopenia (28.3%), anemia (24.8%) and neutropenia (11.2%). The treatment discontinuation rate was 14.7% for niraparib treated patients and 2.2% for control. The rates of myelodysplastic syndrome (MDS) and acute myeloid leukemia (AML) were similar in the niraparib (1.3%) and control (1.2%) arms. There were no treatment-related deaths. CTCAE version 4.0 was used when reporting AEs.

Baseline Platelet Count and Weight as Predictors of Thrombocytopenia.

A retrospective analysis of ENGOT-OV16/NOVA and PN001 study data identified baseline body weight of <77kg (170 lbs) or baseline platelet count <150,000/µL as significant predictors for early dose modification. Lower baseline platelets ($<180x10^9$/L) were associated with an increased frequency of thrombocytopenia Grade ≥1 (76%) or Grade ≥ 3 (45%) compared to patients with higher baseline platelet counts. In addition, an exploratory analysis in the ENGOT-OV16/NOVA study showed Grade ≥3 SAEs, treatment-related adverse events, and treatment-related adverse events leading to dose modification or treatment discontinuation occurred more commonly in the weight <58 kg cohort than in the ≥77 kg cohort. In the cohort of patients with a body weight <58 kg, approximately 80% of patients had a dose reduction compared to 59% of patients with a weight greater than or equal to 77 kg. Finally, a classification tree approach was used to refine the best cut-off points for predicting the likelihood of a patient developing ≥Grade 3 thrombocytopenia within 30 days after the first dose of niraparib. The results of the model show that the subgroup of patients with a baseline body weight <77 kg or baseline platelet count <150,000 µL had a grade 3/4 thrombocytopenia rate in the first 30 days of 35.4% compared to 11.5% in the group of patients with a body weight >77 kg and a platelet count >150,000 µL.

2.4 Background TSR-042

TSR-042 is an IgG4 humanized monoclonal antibody that binds with high affinity to PD-1, resulting in inhibition of binding to PD-L1 and PD-L2. This antibody was generated based on a proprietary platform that utilizes affinity maturation to select highly-specific antibodies with desired functional characteristics. The functional antagonist activity of TSR-042 was confirmed in a mixed lymphocyte reaction assay, demonstrating enhanced interleukin-2 (IL-2) production upon addition of TSR-042. Furthermore, TSR-042 has an acceptable safety profile based on toxicology studies in cynomolgus monkeys. Additional information on the nonclinical and clinical experience with TSR-042 can be found in the TSR-042 IB.

2.4.1 Nonclinical Experience

TSR-042 binds with high affinity to human and cynomolgus monkey PD-1. TSR-042 blocks binding of soluble ligands to human PD-1 expressed on the surface of Chinese hamster ovary cells, with a 50% maximum inhibitory concentration (IC_{50}) of approximately 1 nM. TSR-042 enhances T cell activation, as measured by the production of IL-2from activated CD4+ T cells, with a 50% maximum effective concentration (EC_{50}) of approximately 1 nM. Full PD-1 receptor occupancy achieved by TSR-042 in human and cynomolgus monkey T cells from peripheral blood mononuclear cells was determined to occur at concentrations of approximately 1 μg/mL.

Linear pharmacokinetic (PK) was observed for TSR-042 over the dose range tested of 10 to 100 mg/kg. Sex had no effect on exposure. The volume of distribution at steady state was low and suggested minimal tissue penetration, which is commonly observed for therapeutic monoclonal antibodies. Weekly administration resulted in approximately 2- to 3-fold increase in TSR-042 exposure.

Administration of TSR-042 by a weekly IV dose (5 total doses) to cynomolgus monkeys at doses of 0, 10, 30, or 100 mg/kg was well tolerated and did not result in any TEAEs on clinical signs, body weight, food consumption, ECG, ophthalmology, safety pharmacology parameters, clinical pathology, gross pathology, organ weight, or histopathology. The no-observed-adverse-effects level was ≥100 mg/kg in this study.

2.4.2 Clinical Experience

TSR-042 has been evaluated in one Phase 1 study to date. Study 4010-01-001 is an ongoing first-in-human Phase 1 study of TSR-042 to evaluate the safety and tolerability, PK, pharmacodynamics, and clinical activity of TSR-042 in patients with advanced solid tumors. The study is being conducted in 2 parts:

- Part 1 (dose escalation) of the study used a modified 3 + 3 design to evaluate 3 ascending weight-based doses of TSR-042 as follows: 1, 3, and 10 mg/kg administered every 2 weeks (Q2W) via IV infusion.
- Part 2 of the study is being conducted in 2 subparts (Part 2A and Part 2B) to explore the safety and clinical activity of TSR-042 administered as a fixed dose (ie, not weight based).

- In Part 2A, following the completion of Part 1, the safety and tolerability of TSR-042 were evaluated at fixed doses of 500 mg every 3 weeks (Q3W) and 1,000 mg every 6 weeks (Q6W) using a modified 6 + 6 design with up to 24 patients (6 patients/cohort).
- In Part 2B, the clinical activity, tolerability, and safety of TSR-042 at the RP2D will be evaluated in patients with specific tumor types. Up to 5 tumor types may be investigated in 6 expansion cohorts with approximately 65 patients enrolled in each cohort.

As of 21 January 2017, safety data are available for 31 patients receiving TSR-042. In Part 1, 21 patients were treated with a weight-based dose of TSR-042 and had at least 4 weeks of safety monitoring: 6 patients received a dose of 1 mg/kg, 3 patients received a dose of 3 mg/kg, and 12 patients received a dose of 10 mg/kg, all administered Q2W. Dose escalation continued to a maximally administered dose of 10 mg/kg Q2W, and a maximum tolerated dose was not identified. No dose-limiting toxicities (DLTs) were observed.

In Part 2A, 6 patients received a fixed dose of 1,000 mg Q6W and 6 patients received a fixed dose of 500 mg Q3W; these patients completed at least 6 weeks or 3 weeks of safety monitoring, respectively. No DLTs were observed, and both doses were declared to be safe. Based on the PK/pharmacodynamic profile and safety and tolerability data from the dose regimens evaluated in Part 1 and Part 2A, the recommended dose regimen to be used in Part 2B was determined to be 500 mg Q3W for 4 doses followed by 1,000 mg Q6W thereafter.

As of 21 January 2017, the most commonly observed TEAEs (any grade) were fatigue (29.0%); nausea (25.8%); dehydration, hypokalemia, and hypomagnesemia (19.4% each); decreased appetite, constipation, and vomiting (16.1% each); abdominal pain, arthralgia, back pain, and pruritus (12.9% each); and increased blood creatinine, diarrhea, dyspnea, muscle spasms, pleural effusion, and rash (9.7% each).

2.5 TSR-042 and Niraparib Combination Treatment

2.5.1 Nonclinical Experience

The efficacy and tolerability of niraparib in combination with anti-PD-1 therapy was evaluated in several nonclinical models. The combination was well tolerated in all of these studies. The combination was first tested in a homologous recombination-deficient ovarian cancer mouse model derived from *BRCA* null genetic background,[79] as PARP inhibition was previously shown to increase immune cell infiltration in *BRCA*-deficient models.[80] In a study of a ovarian carcinoma mouse model,[81] niraparib (50 mg/kg orally [PO] QD) and TSR-042 (5 mg/kg intraperitoneally [IP] twice weekly [BIW]) were administered to mice either alone or in combination for 16 days. The combination was tolerated with no treatment-related death. Almost all the tumors achieved complete regression upon treatment with niraparib, TSR-042, and the combination. Complete regression was first observed on treatment Day 16 in 2 of 6, 1 of 6, and 4 of 6 mice from the niraparib, TSR-042, and combination groups, respectively. These results suggest that the therapeutic approach of combining niraparib with TSR-042 may provide additional benefit for patients with homologous recombination-deficient tumors.

Niraparib and anti-PD-1 combination treatment has also been evaluated in several syngeneic models representing breast cancer 1 and breast cancer 2 (*BRCA1/2*) wild-type tumors, one of which was the breast cancer mouse model LPA1-T22. In study of a syngeneic transplant breast cancer model, niraparib (50 mg/kg PO QD) and anti-PD-1 antibody (10 mg/kg IV BIW) were administered to mice either alone or in combination for 15 days. While these tumors were moderately responsive to niraparib or anti-PD-1 antibody alone, with average tumor growth inhibition of approximately 50% for niraparib and 30% for PD-1 antibody, synergistic anti-tumor activity with near-complete tumor growth inhibition (>95%) was achieved with the combination.[82] In a similar study using the lung squamous syngeneic model KLN205, stronger tumor growth inhibition was observed for the combination (52.3%) than for niraparib alone (36.7%) or anti-PD-1 alone (30.5%).[83]

Moreover, a window opportunity trial assessing PARP inhibitor Olaparib as neoadjuvant treatment in type I endometrial cancer, has shown a significant inhibition for cyclin D1 [84]. Additionally, ARD1A status was related with PARP1 and cyclin D1 expression [84].
ARID1A deficiency has been shown to promote mutagenesis, and is correlated with an increased mutational load, elevated numbers of tumor-infiltrating lymphocytes, and PD-L1 expression. Interestingly, treatment with anti-PD-L1 antibody reduced tumor burden and prolonged survival of mice bearing ARID1A-deficient but not ARID1A-wild-type ovarian tumors. [85]. Together, these data support the therapeutic approach of combining niraparib with anti PD-1 agent in endometrial cancer.

2.6 Rationale

Standard treatment options for recurrent endometrial cancer are limited, and consist of chemotherapy, hormonal therapies and palliative care support. However, treatment responses are unpredictable, modest and often short-lived. In addition, no approved targeted therapies or reliable molecular biomarkers that predict the clinical benefit of targeted therapies is available for recurrent endometrial cancer; highlighting the need for new treatment options.

PTEN inactivation is the most frequent genetic aberration in endometrial cancer, predominantly in type I endometrial histology. In addition to type I endometrial cancer tumors, serous endometrial cancers (type II) appear to have a similar genetic background to serous ovarian carcinoma, including hallmarks of deficiency in DNA repair. PTEN has been implicated in the DNA damage response and homologous recombination process; and preclinical data have demonstrated increased sensitivity to PARP inhibition in PTEN-deficient endometrial cancer cell lines compared to wild-type PTEN cell lines [86, 87].

Preclinical data has demonstrated that in addition to direct cytotoxic effects on cancer cells, DNA damaging agents such as PARP inhibitor may promote immunogenic cell death, alter the inflammatory tumor microenvironment and stimulate neoantigen production, thereby activating an antitumour immune response [88]. Moreover, type I endometrial cancer treated with PARP inhibitors have been linked to a cyclin D1 and ARID1 deficiency, which has shown to be a predictive of response to checkpoint inhibitor treatment in xenografts [84]

2.6.1 Rationale for Targeting Homologous Recombination Deficiency (HRD) in Endometrial Cancer

In the past decade, advances in our understanding of the mechanisms involved in the DNA repair pathway and its regulation has led to the recognition of their importance as a potential cancer therapeutic strategy. The recent landmark genomic characterization by the TCGA has helped delineate the clearest picture of endometrial carcinogenesis. Whilst no specific conclusions were reached regarding the proportion of endometrial tumors harboring homologous recombination deficiencies, an interpretative analysis of the report suggests the role of DNA repair deficiencies in endometrial cancer [14]. Hotspot mutations in POLE, a catalytic subunit of DNA polymerase epsilon involved in nuclear DNA replication and repair, defined the POLE ultramutated subset. Furthermore, the copy-number low subgroup harbors high RAD50 expression, which is associated with the DNA repair process [14]. Furthermore, ATR mutations were found in 7% cases, and a significant proportion of ATR mutations (15%) were described in MSI positive subgroup [14].

Recently, the role of PTEN regulating the maintenance of genomic stability has been reported, suggesting that PTEN may be considered as a critical factor involved in the "BRCAness" phenotype in endometrial cancer [89]. The combination of PTEN deficient cells and PARP inhibition may have synergistic effects. PTEN encodes a phosphatase that negatively regulates the PI3K/AKT/mTOR pathway; PTEN also contributes to maintaining genomic stability by regulating the expression of RAD51 and CHK1 function, key proteins in homologous recombination DNA repair pathway [86, 90]. *In vitro* experiments have demonstrated that PTEN-deficient endometrial cell lines fail to elicit RAD51 to DNA damage sites and may be sensitive to PARP inhibition through synthetic lethality process [86]. The role PTEN plays in determining response to PARP inhibitors has been also correlated with a nuclear activity of the protein, suggesting that the absence of nuclear PTEN expression by IHC could predict patient response [87]. However, a recent attempt failed to demonstrate correlation between PTEN deficiencies and response to PARP inhibitor (Olaparib- AZD2281) in endometrial cancer cell lines [91].

The loss of DNA MMR protein may be relevant to determine the importance of HRD in endometrial tumors. The MSI phenotype reported up to 20-30% of sporadic endometrial cancers, is a marker of an underlying defect in one of the MMR genes (principally *MLH1* or *MSH2*) or aberrant methylation of the *MLH1* promoter [92]. The TCGA report identified that MSI positive endometrioid tumors had a mutation rate approximately 10-fold greater than microsatellite-stable (MSS) endometrioid tumors. Among the most common genetic alterations related with DNA repair found due to the hypermutated genomic status of MSI positive tumors are Rad50 and MRE11 [14]. MRE11 protein forms part of the MRE11-Rad50-NBS1 complex, the primary sensor involved in DSBs repair, and recently, somatic MRE11 gene mutation have been shown to increase PARP inhibition sensitivity in endometrial cancer *in vitro* [93].

Understanding more about the molecular abnormalities involved in the sensitivity to PARP inhibition and defining potential predictive biomarkers is critical to rapidly advancing the field of PARP inhibition therapy in "BRCAness" tumors, such as endometrial cancer, and improves clinical outcomes of patients with recurrent endometrial cancer. Given the heightened prevalence

of PTEN deficiency in endometrial cancer and the preclinical evidence, these tumors may prove to be another rational target for PARP inhibition.

2.7 Correlative Studies Background

2.7.1 PTEN Immunohistochemistry Analysis

The tumor suppressor gene PTEN negatively regulates the PI3K/mTOR pathway that is implicated in cell growth and proliferation. PTEN inactivation is the most frequent genetic aberration in endometrial cancer, predominantly in type I endometrial tumors. Several mTOR inhibitors have demonstrated activity and generally favorable toxicity profiles as single agents in endometrial cancer. However, no predictive biomarker or combinations of biomarkers for mTOR inhibitor activity have been identified [94]. In addition, PTEN has been implicated in the DNA damage response and homologous recombination process; and preclinical data have demonstrated increased sensitivity to PARP inhibition in PTEN-deficient endometrial cancer cell lines compared to wild-type PTEN cell lines [87, 91].

PTEN mutation is one, though not the only, mechanism for functional PTEN loss. Given PTEN mutation is seen in up to 80% of the endometrioid subtype [95], the most common histology subtype in endometrial cancers [6], these tumors may prove to be another rational target for PARP inhibition. The overall incidence of PTEN mutation in endometrial cancers of all histological subtypes has been found to be 34-43% [96-98], though the incidence of PTEN protein loss on IHC is higher at 64% [96], suggesting there is a broader group of patients who may potentially benefit from a PARP inhibition treatment strategy. The current cohort-study design will enroll patients regardless the PTEN status across different endometrial cancer histologies and grades of disease, allowing a complete assessment of the activity of niraparib in endometrial cancer. Therefore, patients with relapsed endometrial cancer who have failed to platinum-based chemotherapy will be evaluated utilizing a design that allows a potential patient enrichment strategy.
Loss of PTEN expression on IHC has been shown to correlate with PTEN inactivation [96]. In fact, examining for PTEN loss on IHC has been shown to be more sensitive than gene sequencing for PTEN mutation, for detecting underlying loss of functional of the protein [96]. The inactivation of PTEN protein was demonstrated through elevated phosphorylated S6 (pS6) level, which is a product of PI3K-AKT activation, a pathway that is usually suppressed by PTEN. PTEN negative tumors, as determined on IHC, had significantly higher pS6 scores than PTEN positive tumors [96]. As expected, majority of PTEN mutation positive tumors were negative on IHC (89%). However, amongst PTEN wildtype tumors, 44% had PTEN IHC loss, supporting the notion that functional PTEN loss could be due to other mechanisms such as epigenetic changes, post transcriptional and post translational modifications [96]. Djordjevic et. all have derived a method of standardizing the scoring of PTEN IHC using the antibody 6H2 that has been significantly correlated with activated AKT (pAKT) [99]. PTEN IHC was scored as positive for tumors showing diffuse positive cytoplasmic and nuclear staining in >90% of cells, while negative if tumors with <1% of cells staining for the PTEN antibody [96]. The intermediate group is described as having a heterogeneous staining pattern. For the purpose of classification, PTEN loss includes tumors that are scored negative and heterogeneous. It is thought that even with a proportion of the tumor staining negative for PTEN in the heterogeneous staining tumors, this represents underlying activation of the PI3K-AKT pathway at least in a subcloan of the tumor [96]. This scoring has

been shown reproducible [96, 100].

2.7.2 MSI analysis

Approximately 30–40% of endometrioid endometrial cancers show loss of DNA mismatch repair (MMR) proteins (MLH1, MSH2, MSH6, and PMS2); in sporadic cases this is secondary to MLH1 promoter hypermethylation, and in hereditary Lynch syndrome it can be caused by mutations in any of the DNA MMR genes. There is preclinical evidence that cells with MMR deficiency have underlying defects in the homologous recombination repair pathway [101]. This may serve as a common mechanism for sensitivity to PARP inhibition. Furthermore, PTEN mutation has been found to be more prevalent in the MSI positive than MSS endometrioid EC (PTEN mutational rate of 86% and 33% respectively) [97, 98]. This study will encompass a MSI analysis to better understand the role of loss of DNA mismatch repair proteins as a predictive biomarker for antitumoral activity of niraparib.

2.7.3 HRD status

Presence of HRD in the tumor has been shown to predict for sensitivity to PARP inhibition in high grade serous ovarian cancer, as demonstrated through the maintenance studies involving rucaparib and niraparib in the ARIEL 2 and NOVA clinical trials respectively. It is therefore also highly of interest to assess HRD in endometrial cancer as a predictive biomarker to niraparib response. As mentioned, PTEN inactivation in endometrial cancer may contribute to the tumor's degree of underlying HRD. In the proposed study, endometrial cancers will be tested for underlying HRD. This may be performed using Myriad HRD test, a commercially developed companion diagnostic used in the NOVA phase III trial, and was able to identify a subgroup of patients with enhanced response to niraparib. Alternatively, a screening panel will be developed to include genes involved in cell regulation, DNA replication, DNA recombination and repair pathways. The analyses will be performed with next generation sequencing (NGS) for mutations in HRD genes and/or IHC for expression levels.

2.7.4 Genomic analysis

Next Generation Sequencing technology will be used to profile tumor DNA to correlate genomic alterations with response. We propose targeted panel whole-exonic sequencing (Advanced Molecular Diagnostics Lab - AMDL, CAP/CLIA; S. Kamel-Reid) or whole exome (or genome) sequencing (Genomics Program/Dr. Trevor Pugh), after examination of all slides available, the most suitable, representative blocks will be chosen.

2.7.5 Immune markers

Tumor-infiltrating lymphocytes (TILs) express PD-1 receptor (programmed cell death 1) and are activated by the ligand PD-L1 or PD-L2 expressed on tumor cells, macrophages and dendritic cells. PD-1 acts as checkpoint, downregulating immune response mediated by T-cell. Inhibition of PD-1 or PDL-1 results in the activation of an immune response against tumour cells.

3. PATIENT SELECTION

3.1 Inclusion Criteria

To be considered eligible to participate in this study, patients must meet all of the following requirements:

3.1.1 Histologically confirmed epithelial endometrial cancer. All histologic subtypes are allowed except for endometrial sarcoma, carcinosarcoma, clear cell, mixed and adenosquamous tumors.

3.1.2 Patients must have radiographic evidence of disease progression following the most recent line of treatment.

3.1.3 Patients must have previously received at least one line of platinum-based chemotherapy. Prior hormonal and immunotherapy are allowed. There is no restriction on the total number of lines of prior therapy.

3.1.4 Patients must have measurable disease, defined as at least one lesion that can be accurately measured in at least one dimension (longest diameter to be recorded for non-nodal lesions and short axis for nodal lesions) as ≥10 mm with CT scan, MRI, or calipers by clinical exam, and ≥15mm for nodal lesions. See Section 11 for the evaluation of measurable disease. Areas of previous radiation may not serve as measurable disease unless there is evidence of progression post radiation. A biopsied lesion may be used as the target lesion if it is the only site of measurable disease.

3.1.5 Patients must have archival tumor sample available for PTEN analysis. If archival tissue is not available, the patient will have the option to undergo biopsy where feasible.

3.1.6 Age ≥18 years old on day of consent. As no dosing or adverse event data are currently available on the use of niraparib in patients <18 years of age, children are excluded from this study, but could be eligible for future pediatric trials.

3.1.7 Eastern Cooperative Group (ECOG) performance status ≤ 2 (see Appendix A).

3.1.8 Life expectancy of greater than 12 weeks.

3.1.9 Adequate organ and marrow function as defined be-low:

- Hemoglobin ≥ 90 g/L
- Leukocytes (WBC) ≥ 3.0×10^9/L
- Absolute neutrophil count ≥ 1.5×10^9/L
- Platelets ≥ 100×10^9/L
- Total bilirubin ≤ 1.5 × institutional upper limit of normal (ULN)
- AST(SGOT)/ALT(SGPT) ≤ 3.0 × institutional ULN unless liver metastases are present, in which case they must be ≤ 5 x ULN
- Creatinine ≤ 1.5 × institutional ULN OR calculated creatinine clearance ≥ 60 mL/min using Cockcroft-Gault equation

– Serum albumin ≥ 28g/L

3.1.10 Ability to understand and willing to sign a written informed consent document.

3.1.11 Women must agree to not donate blood during the study or for 90 days after the last dose of study treatment

3.1.12 Women of child-producing potential must agree to use two highly effective contraceptive methods prior to study entry, during study participation, and for at least 150 days after the last administration of study medication. A serum pregnancy test within 72 hours prior to the initiation of therapy will be required for women of childbearing potential.

Highly effective contraception methods include:
• Total abstinence when this is in line with the preferred and usual lifestyle of the subject. Periodic abstinence (e.g., calendar, ovulation, symptothermal, post-ovulation methods) and withdrawal are not acceptable methods of contraception
• Male or Female sterilization (have had surgical bilateral oophorectomy with or without hysterectomy) or tubal ligation at least six weeks before taking study treatment. In case of oophorectomy alone, only when the reproductive status of the woman has been confirmed by follow up hormone level assessment
• Male sterilization (at least 6 months prior to screening). For female subjects on the study the vasectomized male partner should be the sole partner for that subject.
• Combination of any two of the following (a+b or a+c, or b+c):
a. Use of oral, injected or implanted hormonal methods of contraception or other forms of hormonal contraception that have comparable efficacy (failure rate <1%), for example hormone vaginal ring or transdermal hormone contraception
b. Placement of an intrauterine device (IUD) or intrauterine system (IUS)
c. Barrier methods of contraception: condom or occlusive cap (diaphragm or cervical/vault caps) with spermicidal foam/gel/film/cream/vaginal suppository

In case of use of oral contraception women should have been stable on the same pill for a minimum of 3 months before taking study treatment.
Note: Female patients of childbearing age are defined as follows:
• Patients with regular menses
• Patients, after menarche with amenorrhea, irregular cycles, or using a contraceptive method that precludes withdrawal bleeding
• Women who have had tubal ligation

Female patients may be considered to NOT be of childbearing potential for the following reasons:
• The patient has undergone total abdominal hysterectomy with bilateral salpingo-oophorectomy or bilateral oophorectomy
• The patient is medically confirmed to be menopausal (no menstrual period) for 24 consecutive months

Additional Cohort II inclusion Only:

3.1.13 International normalized ratio (INR) or prothrombin time (PT) ≤ 1.5 x ULN unless patient is receiving anticoagulant therapy as long as PT or partial thromboplastin (PTT) is within therapeutic range of intended use of anticoagulants. Activated partial thromboplastin time (aPTT) ≤ 1.5 x ULN unless patient is receiving anticoagulant therapy as long as PT or PTT is within therapeutic range of intended use of anticoagulants.

3.1.14 Participant receiving corticosteroids may continue as long as their dose is stable for at least 4 weeks prior to initiating protocol therapy.

3.1.15 Participant must agree not to breastfeed during the study or for 150 days after the last dose of study treatment.

3.2 Exclusion Criteria

Patients will not be eligible for study entry if any of the following criteria are met:

3.2.1 Chemotherapy or biologic agents (e.g. cytokines or antibodies) received within 4 weeks of starting study treatment.

3.2.2 Hormonal therapy within 2 weeks of starting study treatment.

3.2.3 Pelvic radiotherapy (as treatment of primary disease) within 4 weeks, or palliative radiotherapy encompassing > 20% of the bone marrow within 1 week of starting study treatment.

3.2.4 Previous treatment with a PARP inhibitor, or any other targeted therapy directed against the homologous recombination pathway.

3.2.5 Patients who are receiving any other investigational agents, or have received investigational agents within ≤ 4 weeks, or within a time interval less than at least 5 half-lives of the investigational agent, whichever is shorter, prior to initiating protocol therapy.

3.2.6 Ongoing ≥ Grade 2 toxicities related to prior cancer therapy, with the exceptions of alopecia, neuropathy, lymphopenia and skin depigmentation.

3.2.7 Received transfusion (platelets or red blood cells) ≤4 weeks of the first dose of study treatment.

3.2.8 Major surgery within 4 weeks of registration or ongoing clinically significant post-surgical complications. Study biopsy is not considered major surgery.

3.2.9 Known brain or leptomeningeal metastases, except if stable for greater than 28 days following definitive treatment. Definitive treatment is defined as whole brain radiation, gamma-knife surgery, surgery, or any combination of the above. The patient must have no new or progressive signs or symptoms related to the CNS disease and must be either off or taking a stable dose of corticosteroids. A scan to confirm the absence of brain metastases is not required. Patients with spinal cord compression may be considered if they have received treatment, either surgery or radiation for this and evidence of clinically stable disease for 28 days.

3.2.10 Known history of myelodysplastic syndrome (MDS)/acute myeloid leukemia (AML).

3.2.11 Baseline corrected QT (QTc) interval prolongation > 470 milliseconds.

3.2.12 History of bowel obstruction within 3 months, or other reason preventing effective oral administration of medication.

3.2.13 Known allergic reactions attributed to niraparib or its components.

3.2.14 Uncontrolled inter-current illness including, but not limited to, ongoing or active infection, symptomatic congestive heart failure, unstable angina pectoris, cardiac arrhythmia, or psychiatric illness/social situations that would limit compliance with study requirements.

3.2.15 Patients with a history of other malignancy ≤ 3 years prior to registration, with the exceptions of a) cone-biopsied in situ carcinoma of the cervix uteri; b) basal or squamous cell carcinoma of the skin. All second malignancies in this context should be discussed with the PI.

3.2.16 Any other condition that would, in the Investigator’s judgment, contraindicate the patient’s participation in the clinical study due to safety concerns or compliance with clinical study procedures, e.g., infection/inflammation, social/ psychological issues.

Additional exclusion for Cohort II Only:

3.2.17 Previous treatment with anti PD-1, anti PD-L1, anti PD-L2, anti CTLA4 agents.

3.2.18 History of fistula, or high-risk of developing a fistula.

3.2.19 Diagnosis of immunodeficiency or systemic steroid therapy or other form of immunosuppressive therapy within 7 days prior to initiating the protocol therapy.

3.2.20 Known history of human immunodeficiency virus (type 1 or 2 antibodies).

3.2.21 Known active hepatitis (e.g., hepatitis B surface antigen [HBsAg] reactive) or hepatitis C (e.g., hepatitis C virus [HCV] ribonucleic acid [qualitative] is detected).

3.2.22 Active autoimmune disease that has required systemic treatment in the past 2 years (ie, with use of disease-modifying agents, corticosteroids, or immunosuppressive drugs). Replacement therapy (eg, thyroxine, insulin, or physiologic corticosteroid replacement therapy for adrenal or pituitary insufficiency, etc.) is not considered a form of systemic treatment.

3.2.23 History of interstitial lung disease.

3.2.24 Received a live vaccine within 14 days of initiating protocol therapy.

3.2.25 History of ≥ Grade 3 immune-related AE with prior immunotherapy, with the exception of non-clinically significant lab abnormalities.

3.3 Inclusion of Women and Minorities

Women of all races and ethnic groups are eligible for this trial. This study is designed to include minorities as appropriate. However, the trial is not designed to measure differences in intervention effects. The population of Southern Ontario is ethnically diverse and the proportion of different ethnic groups in the community is provided in the table below. Universal access to health care will ensure that there is no discrimination on the basis of race or gender (Guide to Canadian Human Rights Act: www.chrc-ccdp.ca/public/guidechra.pdf). Individual hospital registries and databases do not routinely collect racial data, under the direction of the Canadian Human Rights Code.

The population demographics and distribution of minorities in Canada is included in the following table:

Table: Visible minority population by Province (2001 Census)

	British Columbia		Alberta		Ontario		Nova Scotia		Total	
**Total population of province**	3,868,870		2,941,150		11,285,550		897,570		**18,993,140**	
**Visible Minorities**	**Population**	**%**	**Population**	**%**	**Population**	**%**	**Population**	**%**	**Population**	**%**
**Black**	25,465	1%	31,390	1%	411,095	4%	19,670	2%	487,620	**3%**
**Asian**	768,435	20%	268,660	9%	1,513,825	13%	12,630	1%	2,563,550	**13%**
**Latin American (Hispanic)**	23,880	1%	18,745	1%	106,835	1%	520	0%	149,980	**1%**
**Visible minority, not included elsewhere**	4,195	0%	4,220	0%	78,915	1%	1,170	0%	88,500	**0%**
**Multiple visible minority**	14,465	0%	6,910	0%	42,375	0%	535	0%	64,285	**0%**
**Total Visible minority population**	836,440	22%	329,925	11%	2,153,045	19%	34,525	4%	3,353,936	**18%**

Source: Statistics Canada, Census of Population.

Data from our consortium has been compiled regarding the representation of minorities on previous clinical trials, and the distribution is as follows:

Population Percentage of Minority and Gender of entering PMHC Trials			
	2010	2011	2012
Visible Minorities			
Black	0.9	2.3	1.2
Asian	10.1	10.9	11.6
Hispanic	10.1	2.3	3.5
**Total**	**21.1**	**15.5**	**16.3**
**Women**	**59.6**	**56.6**	**44.2**

4. REGISTRATION PROCEDURES

4.1 General Guidelines

The Central Office Coordinator at the Drug Development Program Central Office will enter eligible patients on study centrally. The required forms (Registration Checklist) will be provided upon site activation.

Following registration, patients should begin protocol treatment within 7 days. Issues that would cause treatment delays should be discussed with the Principal Investigator (cc the central office study coordinator). If a patient does not receive protocol therapy following registration, the patient's registration on the study may be cancelled. The Central Office Coordinator should be notified of cancellations as soon as possible.

4.2 Registration Process

Prior to registering a patient, each institution must have submitted all necessary regulatory documentation to the Central Office. The registration checklist will only be sent once this has been received.

No patient can receive protocol treatment until registration with the Central Office has taken place. All eligibility criteria must be met at the time of registration. There will be no exceptions. Any questions should be addressed with the Central Office prior to registration.

To register a patient, the following documents are to be completed by the research nurse or data manager and sent / faxed to the Central Office Coordinator:

- Signed patient consent form
- Registration Checklist CRF signed by the investigator

To complete the registration process, the Central Office will review the checklist and once eligibility has been confirmed:

- Assign a patient study number
- Confirm the starting dose
- Register the patient on the study

- Fax or e-mail the confirmation worksheet with the patient study number and dose to the participating site

To ensure immediate attention is given to the faxed checklist, each site is advised to also call the Central Office Coordinator listed on the front sheet. Patient registration will be accepted between the hours of 9am to 5pm ET Monday to Friday, excluding Canadian statutory holidays when the Central Office will be closed.

5. TREATMENT PLAN

5.1 Agent Administration

Cohort I

Niraparib 100 mg capsules will be administered orally continuously as a flat-fixed daily (QD) dose (100 mg, 200 mg or 300 mg daily). Capsules should be swallowed whole without chewing. Fasting is not required. Patients should take doses at approximately the same times each day. The patient will be requested to maintain a medication diary of each dose of study medication. However, the actual number of capsules taken by the patient must be calculated from the number of capsules dispensed and returned. The medication diary will be returned to clinic staff at the end of each cycle and reviewed by the study staff.

Patients must be instructed to return unused study drugs to the site at discontinuation or completion of treatment. The site personnel must ensure that the appropriate dose of the study drug is administered and that the drug accountability is performed.

Treatment will be administered on an outpatient basis. Reported adverse events and potential risks are described in Section 7. Appropriate dose modifications are described in Section 6. No investigational or commercial agents or therapies other than those described below may be administered with the intent to treat the patient's malignancy.

**Regimen Description**				
**Agent**	**Dose**	**Route**	**Schedule**	**Cycle Length**
Niraparib – starting dose	300/200mg**	Oral	Daily at the same time each day	28 days
**Doses as appropriate for assigned dose level; refer to Table 5.1-1 and section 6.1				

Initial dose of niraparib will be determined by Cycle 1 Day 1 weight and platelet count.

Table 5.1-1: Niraparib Initial Dose

**Cycle 1 Day 1 Criteria**	**Niraparib Dose**
Weight ≥77 kg **and** Platelet count ≥150x10^9 L	300 mg
Weight <77 kg **or** Platelet count <150x10^9 L	200 mg

Cohort II

Niraparib 100 mg capsules will be administered orally continuously as a flat-fixed daily (QD) dose (100 mg, 200 mg or 300 mg daily). Capsules should be swallowed whole without chewing. Fasting is not required. Patients should take doses at approximately the same times each day. The patient will be requested to maintain a medication diary of each dose of study medication. However, the actual number of capsules taken by the patient must be calculated from the number of capsules dispensed and returned. The medication diary will be returned to clinic staff at the end of each cycle and reviewed by the study staff.

Patients must be instructed to return unused study drugs to the site at discontinuation or completion of treatment. The site personnel must ensure that the appropriate dose of the study drug is administered and that the drug accountability is performed.

Treatment will be administered on an outpatient basis. Reported adverse events and potential risks are described in Section 7. Appropriate dose modifications are described in Section 6. No investigational or commercial agents or therapies other than those described below may be administered with the intent to treat the patient's malignancy.\

TSR-042 will be administered via a 30-minute (-5-minute/+15-minute infusion window allowed) IV infusion on Day 1 of every 21-day cycle (every 3 weeks) at 500 mg on cycles 1 to 4, followed by 1,000 mg on Day 1 of every 42-days (every 6 weeks) thereafter until the patient discontinues study treatment. TSR-042 can be continued for a maximum of 2 years.

<u>Vital signs monitoring during the (pre and post) infusion of TSR-042:</u>
Vital signs (blood pressure, heart rate, temperature, respiratory rate) will be done with the following frequency (schedule):

- First dose: Pre-infusion, End of infusion, and every 30 minutes during observation period (2-hour observation)
- Second dose: If previous infusion was well-tolerated –Vitals at beginning of infusion, end of infusion, and every 30 minutes during observation period (1-hour observation)
- Subsequent doses: If previous infusion were well-tolerated, then Only pre-infusion and post-infusion is required

There is no expected drug interaction.

**Regimen Description**				
**Agent**	**Dose**	**Route**	**Schedule**	**Cycle Length**
Niraparib – starting dose	300/200 mg**	Oral	Daily at the same time each day	21 days
TSR-042	500mg<br><br>1000mg	IV<br><br>IV	Cycle 1 to 4: On day 1<br><br>C5 – onwards: On day 1 every 6 weeks	21 days<br><br>42 days – C5 onwards
**Doses as appropriate for assigned dose level; refer to Table 5.1-1 and section 6.1				

5.2 General Concomitant Medication and Supportive Care Guidelines

All concomitant medications administered during study treatment will be recorded in source and eCRFs.

5.2.1 Hematopoietic Growth Factors

Prophylactic cytokine (Granulocyte Colony-Stimulating Factor [GCSF]) administration should not be given in the first cycle of the study, but may be administered in subsequent cycles according to local guidelines and Section 6.

5.2.2. Anticoagulants and Antiplatelets

The niraparib safety profile includes risk for thrombocytopenia; therefore, patients should be advised practice caution with anticoagulation and antiplatelet drugs. Patients who require such an agent during participation in the study are allowed to continue with close clinical monitoring.

5.2.3 Anticancer Therapy

No other anticancer therapy is permitted during the course of the study treatment for any patient. If the patient discontinues study treatment, this restriction no longer applies, however the patient will remain enrolled in the study for the purpose of collecting subsequent outcomes. Palliative radiotherapy (excluding the pelvic region and/or palliative radiotherapy encompassing >20% of the bone marrow within 1 week of the first dose of study treatment) is allowed for pre-existing small areas of painful metastases that cannot be managed with local or systemic analgesics as long as no evidence of disease progression is present.

5.2.4 Other Concomitant Medications

Niraparib has potential to weakly induce cytochrome P450 (CYP)1A2 in vitro; therefore, patients should be advised to use caution with drugs that are the sensitive substrates for CYP1A2.

Live vaccines within 14 days prior to the first dose of study treatment are not allowed. Seasonal flu vaccines that do not contain live viruses are allowed. Examples of live vaccines include, but are not limited to, the following: measles, mumps, rubella, chicken pox, yellow fever, rabies, bacille Calmette-Guerin, and typhoid (oral) vaccine. Seasonal influenza vaccines for injection are generally killed virus vaccines and are allowed. Intranasal influenza vaccines (e.g., Flu-Mist®) are live attenuated vaccines and are not allowed.

TSR-042 only: Systemic glucocorticoids for any purpose other than to manage symptoms of suspected irAEIs. (Note: Use of inhaled steroids, local injection of steroids, topical steroids, and steroid eye drops are allowed). If medically deemed necessary (e.g., acute asthma or chronic obstructive pulmonary disease exacerbation), Investigators are allowed to use their judgment to treat patients with systemic steroids. In such cases, systemic steroids should be stopped at least 24 hours prior to the next dose of TSR-042

Any other medication which is considered necessary for the patient's welfare, and which is not expected to interfere with the evaluation of the study drug, may be given at the discretion of the Investigator. No other investigational agents are permitted during the entire duration of treatment with study drug.

5.2.5 Miscellaneous

Patients must not donate blood during the study or for 90 days after the last dose of study treatment, in cohort I and II.

A whole blood sample will also be collected for cytogenetic analysis (mutations of select myeloid-associated genes). Testing completed as part of standard of care is sufficient as long as the methods are acceptable to the Sponsor's Scientific Director. The study site must receive a copy of the hematologist's report of aspirate/biopsy findings (which must include a classification according to World Health Organization criteria) and other sample testing reports related to MDS/AML. Report data will be entered into EDC on the appropriate eCRF pages, and the site must keep a copy of the report with the patient's study file.

5.3 Duration of Therapy

In the absence of treatment delays due to adverse event(s), treatment may continue until one of the following criteria applies:

- Any treatment-related Common Terminology Criteria for Adverse Events (CTCAE) Grade 3 or 4 events that have not reverted to baseline or CTCAE Grade 1 within 28 days. At the Investigator's discretion, following dose interruption ≤28 days, patients may be considered for dose reductions provided they have not already undergone the maximum allowed dose reductions to 100mg/day. If upon re-challenging with study treatment at the lowest allowable dose, any CTCAE Grade 3 or 4 adverse events recur, the patient should stop treatment permanently
- <u>If platelet count has not reverted to ≥100x10^9/L within 28 days</u>
- Disease progression as defined by section 11
- Clinical progression
- Diagnosis of MDS or AML (as confirmed by a hematologist)
- Women who become pregnant or are breastfeeding
- Sexually active subjects who refuse to use medically accepted forms of barrier contraception (e.g. male condom, female condom) during the study and for 90 days following discontinuation of study treatment
- Termination of the protocol by a regulatory agency or Sponsor, or study medication can no longer be provided
- Significant non-compliance with the protocol schedule in the opinion of the investigator
- Patients requiring a delay in treatment beyond the allowed timeframe.
- Patients requiring more than 2 dose reductions of niraparib
- Delays in TSR-042 treatment will be allowed for 4 weeks, if there is benefit restarting the treatment, it will be discussed with the PI. TSR-042 can be continued for a maximum of 2

years.

- Intercurrent illness that prevents further administration of treatment
- Patient decides to withdraw from the study, or
- General or specific changes in the patient's condition render the patient unacceptable for further treatment in the judgment of the investigator.
- Necessity for treatment with another anti-cancer treatment prohibited by this protocol.

5.4 Duration of Follow Up

Reason for patient removal from study		Follow-Up Period: 30 (+/-3) days from the End of Study Drug Administration	Follow-Up Period: Every 12 weeks (+/-7 days) from date of last scan until objective disease progression or start of new Therapy[A]	Follow-Up Period: Monthly (+/-3 days) monitoring for adverse event(s)[C], AML/MDS development[D]	Follow-Up Period: Survival (Every 6 Months)[F]
	Objective Disease Progression	X		X	X[E]
	Clinical Progression/ Symptomatic Deterioration	X	X[B]	X	X[E]
	Adverse Events or clinically significant lab value	X	X[B]	X (weekly FU for 4-weeks then monthly)	X[E]
	All other patients	X	X[B]	X	X[E]

NOTES

A. Objective disease progression is measured per RECIST 1.1 (see Section 11.1).
B. Radiological assessments with CT scans need to be performed, with window of +/-7 days from the date CT is due
C. Until adverse events resolves to < grade 2 (or baseline values), becomes stabilized or is no longer related to study treatment. In the case patient discontinuation is due to adverse event or clinically significant lab value, the patient needs to be followed weekly for 4 weeks after last dose (see Section 7.6), and then monthly until resolution of adverse event. All visits are to occur with a window of +/- 3 days from the date of scheduled review.
D. AML/MDS development will be followed every 3 months +/- 2 weeks for a maximum of 2 years
E. Patients who withdraw consent will no longer be followed
F. Patient will be followed up for 2 years

.

5.5 Criteria for Removal from Study

Patients will be removed from study when any one of the criteria below applies:

- Withdrawal of consent by the patient
- Death from any cause
- Patient lost to follow up
- Regulatory closure of the trial

The reason for study removal and the date the patient was removed must be documented in the Case Report Form.

6. DOSING DELAYS/DOSE MODIFICATIONS

6.1 General Guidelines: Niraparib

Dose modifications are listed in Table 6.1-1. Once the dose of study treatment has been reduced, dose re-escalation is not permitted.

If the toxicity requiring dose interruption has not resolved to ≤ CTCAE Grade 1 or baseline during the maximum 4 weeks (28 days) dose interruption period, and/or the patient has already undergone the maximum number of dose reductions or reached the minimum dose of 100 mg QD, the patient must permanently discontinue treatment with niraparib.

For major surgery while on treatment, up to 28 days of drug interruption is allowed.

All dose interruptions and reductions (including any missed doses), and the reasons for the reductions/interruptions, are to be recorded in the electronic case report from (eCRF).

Table 6.1-1: Niraparib Dose Modifications

Dose Level	Initial Dose: 3 capsules per day	Initial Dose: 2 capsules per day
Initial dose	300 mg	200 mg
First dose reduction	200 mg	100 mg
Second dose reduction	100 mg	NA

6.1.1 Dose Modifications for Hematological Toxicities

If dose interruption or modification is required at any point on study because of hematologic toxicity, to ensure safety of the new dose, weekly blood draws for CBC will be required for an additional 4 weeks after the AE has been resolved to the specified levels, after which monitoring every 4 weeks may resume. Weekly blood draws for CBC can be collected either at study site or local laboratories. If the hematologic toxicity has not recovered to the specified levels within 4 weeks (28 days) of the dose interruption period, and/or the patient has already undergone the maximum dose reductions (to a minimum dose of 100 mg QD) then the patient must permanently discontinue treatment with niraparib.

Any patient requiring transfusion of platelets, red blood cells transfusion (except on the first occurrence), or hematopoietic growth factor support must undergo a niraparib dose reduction upon recovery if study treatment is resumed. If no further reductions can be made, niraparib must be permanently discontinued.

The treating physician may consider referral to a hematologist for further evaluation if the treatment-related hematologic toxicities have not recovered to ≤ Grade 1 or baseline within 4 weeks. If a diagnosis of MDS/AML is confirmed by a hematologist, the patient must permanently discontinue study treatment.

Table 6.1.1-1: Dose Modification for Thrombocytopenia

Platelet count	Management/Next Dose for Niraparib
<LLN -100x10$^{9/L}$	No change in dose
<100x10^9/L – 75x10^9/L	First occurrence:<br>Hold until platelet counts are ≥100x10^9/L, with weekly CBC until recovery. Weekly CBCs will continue to be

Platelet count	Management/Next Dose for Niraparib
	monitored for an additional 4 weeks after recovery to ≥100x10^9/L. Resume at same dose level, or reduced dose based on clinical judgement.<br><br>Second occurrence:<br>Hold until platelet counts are ≥100x10^9/L, with weekly CBC until recovery. Weekly CBCs will continue to be monitored for an additional 4 weeks after recovery to ≥100x10^9/L. Resume at a reduced dose.
<75x10^9/L	Hold until platelet counts are ≥100x10^9/L, with weekly CBC until recovery. Weekly CBCs will continue to be monitored for an additional 4 weeks after recovery to ≥100x10^9/L. Resume drug with one dose-level reduction.
Hematologic adverse reaction requiring transfusion	For patients with platelet count ≤10,000/μL, platelet transfusion should be considered. If there are other risk factors such as co-administration of anticoagulation or antiplatelet drugs, consider interrupting these drugs and/or transfusion at a higher platelet count.<br>Resume niraparib at a reduced dose.
▪ If platelet count has not reverted within 28 days of interruption to ≥100x10^9/L, then the patient should be discontinued.<br>▪ For patients with platelet count ≤ 10x10^9/L , prophylactic platelet transfusion per guidelines is to be considered [102].<br>▪ For patients taking anticoagulation or antiplatelet drugs, consider the risk/benefit of interrupting these drugs and/or prophylactic transfusion at an alternate threshold.<br>▪ Patients requiring more than two dose reductions or reduction below 100mg will be discontinued.	

Table 6.1.1-2: Dose Modification for Neutropenia

Neutrophil Count	Management/Next Dose for Niraparib
Grade 1:<br><LLN – 1.5x10^9/L	No change in dose
Grade 2:<br><1.5 – 1.0 x10^9/L	No change in dose
Grade 3:<br><1.0 – 0.5 x10^9/L	Hold until neutrophil count is ≥1.5x10^9/L, with weekly CBC until recovery and continue to monitor weekly CBC for an additional 4 weeks. Resume at a reduced dose.
Grade 4:<br><0.5 x10^9/L	Hold until neutrophil count is ≥1.5x10^9/L, with weekly CBC until recovery and continue to monitor weekly CBC for an additional 4 weeks. Resume at a reduced dose.
▪ If neutrophil count has not reverted within 28 days of interruption to ≥1.5x10^9/L, then the patient should be discontinued.<br>▪ Patients requiring more than two dose reductions or reduction below 100mg will be discontinued.	

Table 6.1.1-3: Dose Modification for Anemia

Anemia	Management/Next Dose for Niraparib
Grade 1:<br>Hemoglobin (Hgb)<br><LLN – 100g/L	No change in dose
Grade 2:<br>Hgb <100 – 80g/L	No change in dose
Grade 3:<br>Hgb <80 g/L; transfusion >1 unit red blood cells indicated	Hold until hemoglobin is ≥90 g/L, with weekly CBC until recovery and continue to monitor weekly CBC for an additional 4 weeks. Resume at reduced dose.
Grade 4:<br>Life-threatening consequences; urgent intervention indicated	Hold until hemoglobin is ≥90 g/L, with weekly CBC until recovery and continue to monitor weekly CBC for an additional 4 weeks. Resume at a reduced dose.
▪ If hemoglobin level has not reverted within 28 days of interruption to ≥$90x10^9$/L, then the patient should be discontinued.<br>▪ Patients requiring more than two dose reductions or reduction below 100 mg will be discontinued.	

There is no recommendation for lymphopenia or total white blood count.

6.1.2 Dose Modifications for Non-Hematological Toxicities

Table 6.1.2-1: Dose Modification for Non-Hematological Toxicities

Event	Management/Next Dose for Niraparib
Grade 1	No change in dose
Grade 2	No change in dose
Grade 3	Hold until AE resolves to baseline or ≤Grade 1 (controlled). Resume with one dose level reduction.
Grade 4	Hold until AE resolves to baseline or ≤Grade 1 (controlled). Resume with one dose level reduction.
▪ Patients with toxicities that do not resolve within 28 days will be discontinued.<br>▪ Patients requiring more than two dose reductions or reduction below 100 mg will be discontinued.<br>▪ Dose reductions for any Grade 2 events that are bothersome to the patient will be permitted per Investigator judgement.	
This table only applies to non-hematological adverse events considered related to study medication and considered clinically significant as per investigator judgement. Clinically non-significant, treatable or reversible lab abnormalities including, but not limited to alkaline phosphatase or gamma-glutamyl transferase, uric acid, or electrolyte abnormalities do not require dose modifications.	

6.2 GENERAL GUIDELINES: TSR-042

AEs (both non-serious and serious) associated with TSR-042 exposure may represent an immunologic etiology. These AEs may occur shortly after the first dose or several months after the last dose of treatment.

In general, TSR-042 must be withheld for drug-related Grade 3 toxicities, as well as for certain immune-related adverse events of interest (irAEIs), but may be resumed upon recovery to Grade ≤1; TSR-042 will be permanently discontinued for any drug-related Grade 4 AE. TSR-042 must be permanently discontinued for certain irAEIs as described in Table 6.2-1.

The specific immune-related AEs typically observed with anti-PD-1 antibodies will be managed according to the American Society of Clinical Oncology Clinical Practice guidelines summarized below [103].

Immune-related Adverse Events of Interest and Guidelines for Management

Given the mechanism of action of TSR-042, it is anticipated that activation of cellular immune system can be manifested as immune-related AEs. Based on available safety data from checkpoint inhibitors, treatment emergent adverse events (TEAEs) with the specific grades listed below were selected as immune-related adverse events of interest (irAEIs). The list of irAEIs may be updated upon emerging data.

Refer to Table 6.2-1 for details on the management of TSR-042 dose delays and discontinuation for specific irAEIs. Detailed guidance for the administration of rescue medications and supportive care are available below. For all irAEIs listed in Table 6.2-1, TSR-042 should be withheld until the patient is clinically and metabolically stable and AEs have resolved to Grade ≤1. If systemic steroids are used as a part of irAEI management, the total dose of daily steroids should be equal to or less than 10mg prednisone at the time of resuming TSR-042.

All treatment delays (including any missed doses) and discontinuations, and the reason for delays or discontinuation of TSR-042, should be documented.

Table 6.2-1: Guidelines for Treatment of Immune-related Adverse Events of Interest

**Toxicity**	**Withhold Treatment for AE Grade**	**Restarting Treatment/Discontinuation**
Diarrhea/colitis	2 to 3	Restart dosing when toxicity resolves to baseline or grade 1.
	4	Permanently discontinue.
AST, ALT, or increased bilirubin	2<br>(AST or ALT > 3 and ≤ 5 × ULN or total bilirubin > 1.5 and ≤ 3 × ULN)	Restart dosing when toxicity resolves to Grade 0 to 1.
	3 or 4<br>(AST or ALT > 5 × ULN or total bilirubin > 3 × ULN)	Permanently discontinue (see exception below).([a])

Toxicity	Withhold Treatment for AE Grade	Restarting Treatment/Discontinuation
T1DM or hyperglycemia	3 or 4 hyperglycemia or T1DM (associated with metabolic acidosis or ketonuria)	Restart dosing in appropriately man aged, clinically and metabolically stable patients, insulin replacement therapy is required.
Immune-related encephalitis	Any grade	Permanently discontinue.
Hypophysitis	2 or 3	For Grade 2 to 3 AEs, hold until hormonal therapy results in return to adequate levels by laboratory values. Treatment may be restarted if patient is stabilized on replacement hormones. For recurrence or worsening of Grade ≥2 hypophysitis after corticosteroid taper has been completed and patient is on adequate hormone replacement therapy, permanently discontinue.
	4	For Grade 4 AEs, permanently discontinue.
Hyperthyroidism	3	Restart dosing when toxicity resolves to Grade 0 to 1.
	4	Permanently discontinue.
Infusion-related reaction	2([b])	Restart dosing when toxicity resolves to Grade 0 to 1.
	3 or 4	Permanently discontinue.
Pneumonitis	2	Restart dosing when toxicity resolves to Grade 0 to 1. If Grade 2 recurs, permanently discontinue.
	3 or 4	Permanently discontinue.
Rash	3	Restart dosing when toxicity resolves to Grade 0 to 1.
	4	Permanently discontinue.
Renal failure or nephritis	2	Restart dosing when toxicity resolves to Grade 0 to 1.
	3 or 4	Permanently discontinue.
Recurrence of AEs after resolution to Grade ≤1	3 or 4	Permanently discontinue.

Abbreviations: AE = adverse event; ALT = alanine aminotransferase; AST = aspartate aminotransferase; T1DM = type 1 diabetes mellitus; ULN = upper limit of normal.

[a] For patients with liver metastasis who begin treatment with Grade 2 AST or ALT, if AST or ALT increases by ≥50% relative to baseline and lasts for at least 1 week, then study treatment should be discontinued.

[b] Upon resolution within 1 hour of stopping drug infusion, the infusion may be restarted at 50% of the original infusion rate (e.g., from 100 to 50 mL/h). Otherwise, study treatment will be withheld until symptoms resolve, and the patient should be pre-medicated for the next scheduled dose.

6.2.1 Rescue Medications and Supportive Care Guidelines

During treatment with TSR-042, patients should receive appropriate supportive care measures for AEs as deemed necessary by the treating Investigator, including but not limited to the items outlined below. Prophylactic cytokines (eg, GCSF) should be administered according to current

ASCO guidelines [104]. Note: It may be necessary to perform additional procedures such as bronchoscopy, endoscopy, or skin photography as part of the evaluation of the AE. The following sections detail specific guidance by type of AE.

Pneumonitis

- Treat with systemic corticosteroids, oral for Grade 2 (e.g., 0.5 to 1 mg/kg/day of prednisone or equivalent) and IV for Grade 3 or 4 (e.g., 1 to 2 mg/kg/day of prednisone or equivalent).
- Administer additional anti-inflammatory measures, as needed.
- Taper corticosteroids when symptoms improve to Grade 1 or less over no less than 4 weeks.
- If Grade 2 and no improvement or worsening over 2 weeks, treat as Grade 3 or 4.
- Consider prophylactic antibiotics for opportunistic infections in the case of prolonged steroid administration.

Diarrhea/Colitis

- Monitor carefully for signs and symptoms of enterocolitis (such as diarrhea, abdominal pain, blood or mucus in stool, with or without fever) and of bowel perforation (such as peritoneal signs and ileus).
- All patients who experience diarrhea/colitis should be advised to drink liberal quantities of clear fluids. If sufficient oral fluid intake is not feasible, fluid and electrolytes should be substituted via IV infusion.
- For Grade 2 diarrhea/colitis that persists >3 days, administer oral corticosteroids (eg, 0.5 to 1.0 mg/kg/day of prednisone or equivalent). If symptoms persist or worsen with steroids, treat as Grade 3 or 4.
- For Grade 3 or 4 diarrhea/colitis that persists >3 days, treat with IV steroids (eg, 1 to 2 mg/kg/day of prednisone or equivalent) followed by high-dose oral steroids.
- Taper corticosteroids when symptoms improve to Grade 1 or less over no less than 4 weeks.

Type 1 Diabetes Mellitus or Grade 3 or 4 Hyperglycemia

For type 1 diabetes mellitus and for Grade 3 or 4 hyperglycemia associated with metabolic acidosis or ketonuria, insulin replacement therapy is required.

Hypophysitis

- Treat with systemic corticosteroids, oral for Grade 2 (eg, 0.5 to 1 mg/kg/day of prednisone or equivalent) and IV for Grade 3 or 4 (eg, 1 to 2 mg/kg/day of prednisone or equivalent).

- Taper corticosteroids when symptoms improve to Grade 1 or less over no less than 4 weeks.
- Replacement of appropriate hormones may be required as the steroid dose is tapered.

Hyperthyroidism or Hypothyroidism

Thyroid disorders have been reported with other PD-1 inhibitors occurring at any time during treatment. Monitor patients for changes in thyroid function (at the start of treatment, periodically during treatment, and as indicated based on clinical evaluation) and for clinical signs and symptoms of thyroid disorders.

- Grade 2 HYPERthyroidism: Consider non-selective beta-blockers (eg, propranolol) as initial therapy.
- Grade 3 or 4 HYPERthyroidism: Treat with an initial dose of IV corticosteroids followed by oral corticosteroids (eg, 0.5 to 1 mg/kg/day of prednisone or equivalent). Taper corticosteroids when symptoms improve to Grade 1 or less over no less than 4 weeks. Replacement of appropriate hormones may be required as the steroid dose is tapered.
- Grade 2 to 4 HYPOthyroidism: Thyroid hormone replacement therapy, with levothyroxine or liothyronine, is indicated per standard of care.

Hepatitis

- Treat with systemic corticosteroids, oral for Grade 2 (initial dose of 1 to 2 mg/kg/day of prednisone or equivalent) and IV for Grade 3 or 4 (1 to 2 mg/kg/day of prednisone or equivalent).
- Taper corticosteroids when symptoms improve to Grade 1 or less over no less than 4 weeks.

Renal Failure or Nephritis

- Treat with systemic corticosteroids, oral for Grade 2 (initial dose of 0.5 to 1 mg/kg/day of prednisone or equivalent) and IV for Grade 3 or 4 (1 to 2 mg/kg/day of prednisone or equivalent).
- Taper corticosteroids when symptoms improve to Grade 1 or less over no less than 4 weeks.

Management of Infusion-Related Reactions

Signs and symptoms usually develop during or shortly after drug infusion and generally resolve completely within 24 hours of completion of infusion. Table 6.2-2. shows treatment guidelines for patients who experience an infusion-related reaction associated with administration of TSR-042.

Table 6.2-2: TSR-042 Infusion Reaction Treatment Guidelines

CTCAE Grade	Treatment	Premedication at Subsequent Dosing
**Grade 1**<br>Mild reaction; infusion interruption not indicated; intervention not indicated	Increase monitoring of vital signs as medically indicated until the patient is deemed medically stable in the opinion of the Investigator.	None.
**Grade 2**<br>Requires infusion interruption but responds promptly to symptomatic treatment (e.g., antihistamines, NSAIDs, narcotics, or IV fluids); prophylactic medications indicated for ≤24 h	Stop infusion and monitor symptoms.<br>Additional appropriate medical therapy may include but is not limited to:<br>• IV fluids<br>• Antihistamines<br>• NSAIDs<br>• Acetaminophen<br>• Narcotics<br>Increase monitoring of vital signs as medically indicated until the patient is deemed medically stable in the opinion of the Investigator.<br>If symptoms resolve within 1 hour of stopping drug infusion, the infusion may be restarted at 50% of the original infusion rate (e.g., from 100 mL/h to 50 mL/h). Otherwise, dosing will be withheld until symptoms resolve, and the patient should be pre-medicated for the next scheduled dose.<br>Patients who develop Grade 2 toxicity despite adequate premedication should be permanently discontinued from further study treatment administration.	Patient may be pre-medicated 1.5 h (±30 min) prior to infusion of TSR-042 with:<br>• Diphenhydramine 50 mg PO (or equivalent dose of antihistamine)<br>• Acetaminophen 500 to 1000 mg PO (or equivalent dose of antipyretic)

CTCAE Grade	Treatment	Premedication at Subsequent Dosing
**Grade 3:**<br>Prolonged (ie, not rapidly responsive to symptomatic medication and/or brief interruption of infusion); recurrence of symptoms following initial improvement; hospitalization indicated for other clinical sequelae (e.g., renal impairment, pulmonary infiltrates)<br>**Grade 4:**<br>Life-threatening; pressor or ventilatory support indicated	**Stop Infusion.**<br>Additional appropriate medical therapy may include but is not limited to:<br>• IV fluids<br>• Antihistamines<br>• NSAIDs<br>• Acetaminophen<br>• Narcotics<br>• Oxygen<br>• Pressors<br>• Corticosteroids<br>• Epinephrine<br>Increase monitoring of vital signs as medically indicated until the patient is deemed medically stable in the opinion of the Investigator.<br>Hospitalization may be indicated.<br>**Patient is permanently discontinued from further study treatment administration.**	No subsequent dosing.

Abbreviations: CTCAE = Common Terminology Criteria for Adverse Events; IV = intravenous; NSAID = nonsteroidal anti-inflammatory drug; PO = oral.

Note: Appropriate resuscitation equipment should be available in the room and a physician readily available during the period of study treatment administration.

7. ADVERSE EVENTS: LIST AND REPORTING REQUIREMENTS

7.1 List of Adverse Events and Reporting Requirements

This study will utilize the Common Terminology Criteria for Adverse Events version 4.0 for toxicity and Adverse Event reporting. A copy of the CTCAE v4.0 can be downloaded from the CTEP home page (http://ctep.cancer.gov). All appropriate treatment areas should have access to a copy of the CTCAE v4.0.

7.1.1 Expected Adverse Events and Protocol-Specific Expedited Adverse Event Reporting for Investigational Agent(s)

Adverse event (AE) monitoring and reporting is a routine part of every clinical trial. The following list of AEs (Section 7.1.1.1) and the characteristics of an observed AE (Section 7.2) will determine whether the event requires expedited reporting as an SAE in addition to routine reporting.

In addition, hospitalizations for routine procedures, protocol treatment, blood sampling, investigations and tissue biopsies are NOT considered SAE in this protocol.

7.1.1.1 Expected Adverse Events for Niraparib

The common expected drug related adverse events seen in Phase I studies of niraparib involving 144 patients include fatigue, nausea, anemia, thrombocytopenia, decreased appetite, neutropenia, vomiting, constipation, leukopenia, diarrhea, insomnia, dyspnea, electrocardiogram (ECG) QT prolongation, headache, stomatitis, hyponatraemia and alopecia. The majority of these events were managed with dose interruption and/or reduction. There were no new safety concerns from the recently completed phase III niraparib maintenance study in ovarian cancer (PR-30-5011-C or NOVA trial). The treatment discontinuation rate in that study was 14.7% for the niraparib arm and 2.2% for control.

Myelodysplastic syndrome (MDS) and AML have been observed in patients receiving treatment with olaparib, a PARP inhibitor, and represent a potential risk for patients receiving niraparib [78]. In the phase III NOVA trial there was no increase incidence of MDS/AML seen in the niraparib treated patients (1.3%) compared with patients who received placebo (1.2%). However, there exists the potential for an increased risk for the development of MDS/AML due to numerous insults to the DNA damage repair pathway.

For complete list of adverse event for Niraparib, please refer to the current Investigator's Brochure of Niraparib.

Adverse Events of Special Interest (AESI)

An Adverse Event of Special Interest is defined as any AE (serious or non-serious) that is of scientific and medical concern specific to the study treatment, for which ongoing monitoring and rapid communication to the Sponsor Institution and to TESARO/GSK is required.

Adverse Events of Special Interest (AESI) for niraparib include the following:

- Myelodysplastic Syndromes (MDS) and Acute Myeloid Leukemia (AML)
- Secondary cancers (new malignancies [other than MDS or AML])
- Pneumonitis
- Embyro-fetal toxicity

AESIs should be reported on SAE Report Forms whether serious or not, as follows:

- MDS and AML along with other secondary cancers should be reported to the Sponsor Institution and to TESARO/GSK upon awareness for any patient who has received niraparib (regardless of the timeframe since the last dose).
- Pneumonitis should be reported to the Sponsor Institution and to TESARO/GSK through 90 days after the last dose of niraparib.

- Embryo-fetal toxicity should be reported as outlined in the Pregnancy reporting section.

Expected Adverse Events for TSR-042

TSR-042 has been evaluated in one Phase 1 study to date. As of 21 January 2017, the most commonly observed AEs (any grade) were fatigue (29.0%); nausea (25.8%); dehydration, hypokalemia, and hypomagnesemia (19.4% each); decreased appetite, constipation, and vomiting (16.1% each); abdominal pain, arthralgia, back pain, and pruritus (12.9% each); and increased blood creatinine, diarrhea, dyspnea, muscle spasms, pleural effusion, and rash (9.7% each).

For complete list of adverse event for TSR-042, please refer to the current Investigator's Brochure of TSR-042.

7.2 Adverse Event Characteristics

- **CTCAE term (AE description) and grade:** The descriptions and grading scales found in the revised NCI Common Terminology Criteria for Adverse Events (CTCAE) version 4.0 will be utilized for AE reporting. All appropriate treatment areas should have access to a copy of the CTCAE 4.0. A copy of the CTCAE 4.0 can be downloaded from the CTEP web site https://ctep.cancer.gov/protocolDevelopment/electronic_applications/ctc.htm

- **Attribution** of the AE:
  - Definite – The AE *is clearly related* to the study treatment.
  - Probable – The AE *is likely related* to the study treatment.
  - Possible – The AE *may be related* to the study treatment.
  - Unlikely – The AE *is doubtfully related* to the study treatment.
  - Unrelated – The AE *is clearly NOT related* to the study treatment.

**UHN Causality Term**	**UHN Causality Definition**	**TESARO/GSK Causality**
Unrelated	The AE *is clearly NOT related* to the study treatment.	Not Related
Unlikely	The AE *is doubtfully related* to the study treatment.	Unlikely Related
Possible	The AE *may be related* to the study treatment.	Possibly Related
Probable	The AE *is likely related* to the study treatment.	Related
Definite	The AE *is clearly related* to the study treatment.	Related

Associated with the use of the *drug/biologic:* There is a reasonable possibility that the experience may have been caused by the drug/biologic.

Life threatening adverse *drug/biologic* experience: Any adverse drug/biologic experience that places the subject, in the view of the investigator, at immediate risk of death from the reaction as it occurred.

Serious adverse *drug/biologic* experience: Any event is an AE occurring at any dose that results in any of the following outcomes:

- Death
- A life-threatening AE (The patient was, in the view of the Investigator, at immediate risk of death from the event as it occurred. It does not mean that the event, had it occurred in a more severe form, might have caused death).
- Hospitalization or prolongation of existing hospitalization (Complications that occur during hospitalization are AEs. If a complication prolongs hospitalization or fulfills any other serious criteria, the event is serious. Hospitalization for elective treatment of a pre-existing condition that did not worsen from baseline is not considered to be an AE).
- A persistent or significant disability/incapacity (A substantial disruption of a person's ability to conduct normal life functions. This definition is not intended to include experiences of relatively minor medical significance such as uncomplicated headache, nausea, vomiting, diarrhea, influenza, accidental trauma (i.e., sprained ankle) that may interfere or prevent everyday life functions but do not constitute a substantial disruption).
- A congenital anomaly/birth defect.
- Important medical events that may not result in death, be life-threatening, or require hospitalization may be considered serious when, based upon appropriate medical judgment, they may jeopardize the patient and may require medical or surgical intervention to prevent one of the outcomes listed in this definition (Examples include allergic bronchospasm requiring intensive treatment in an emergency room or at home, blood dyscrasias or convulsions that do not result in hospitalization or the development of drug dependency or drug abuse).

Any secondary malignancy possibly related to cancer treatment (including AML/MDS) should be reported as an SAE. A secondary malignancy is one related to the treatment of a prior malignancy (and is NOT a metastasis from the initial malignancy).

Events not considered to be serious adverse events are:

- hospitalizations for the routine treatment or monitoring of the studied indication, not associated with any deterioration in condition,
- treatment, which was elective or pre-planned, for a pre-existing condition that is unrelated to the indication under study and did not worsen,
- admission to a hospital or other institution for general care, not associated with any deterioration in condition, or
- treatment on an emergency, outpatient basis for an event not fulfilling any of the definitions of serious given above and not resulting in hospital admission.

Any SAE occurring after the patient has provided informed consent and until 30 days after the patient has stopped study participation must be reported. This includes the phase in which the study protocol interferes with the standard medical treatment given to a patient (e.g. treatment withdrawal during screening phase, change in treatment to a fixed dose of concomitant medication). Serious adverse events occurring more than 30 days after study drug discontinuation need only be reported if a relationship to niraparib or TSR-042 is suspected.

Unexpected adverse *drug/biologic* experience: Any adverse drug/biologic experience, the nature, frequency, or severity of which is not consistent with the product monograph, or not consistent with the risk information described above as a protocol-specific expected adverse event (see "Expected Adverse Events and Protocol-Specific Expedited Adverse Event Reporting Exclusions", above).

7.3 Serious Adverse Event Reporting

7.3.1 Sponsor Notification

Any serious adverse event must be reported to the Central Office within 24 hours of the Investigator at the site learning of the event by completion of an SAE form. The adverse event must be completely described in the SAE report form.

Signs and symptoms surrounding the SAE which occur during or following the course of drug administration must be reported in detail on the subject's SAE report. This description is to include the CTCAE term, time of onset, duration, CTCAE grade, and possible relationship to study treatment, required therapy, and outcome. The SAE Report Form must be signed by the investigator. The subject should be followed until the SAE is resolved, or until in the opinion of the Principal Investigator, reversal of the reaction is not likely to occur.

7.3.2 SAE Follow-up

Follow-up SAE reports are subject to the same timelines as the initial report, and are sent to the same parties to whom the original Serious Adverse Event Form was sent. A new serious adverse event form is completed for the follow-up, stating that this is a follow-up to the previously reported serious adverse event. Each re-occurrence, complication or progression of the original event should be reported as a follow-up to that event. The follow-up information should describe whether the event has resolved or continues, if and how it was treated, and whether the patient continued or discontinued study participation.

7.3.3 REB Notification of SAEs

Investigators must notify their Research Ethics Boards (according to their local REB policies) and file the report in their study files. Documentation as outlined below must be maintained for reportable SAEs. Documentation that serious adverse events (SAEs) have been reported to site REB must be forwarded to the Drug Development Program Central Office and kept on file at the Centre. Documentation can be any of the following:

- Letter, email or stamp from the REB acknowledging receipt
- Email, system generated message, or letter demonstrating the SAE was sent to the REB
- Letter or email demonstrating the SAE was sent to the REB

7.3.4 Health Canada SAE Reporting

All serious, unexpected adverse drug reactions must also be reported by the Drug Development Program Central Office to Health Canada within 15 days if the reaction is neither fatal nor life threatening, and within 7 days if the reaction is fatal or life threatening.

7.3.5 SAE Reporting to TESARO/GSK

To ensure patient safety, each serious adverse event must also be reported by the Central Office to TESARO/GSK (in English) within 24 hours of learning of its occurrence, even if it is not felt to be treatment-related. Follow-up information about a previously reported serious adverse event must also be reported within 24 hours to the Central Office. All SAEs deemed Health Canada reportable should be provided to TESARO / GSK on a MedWatch or CIOMS I form. For other SAEs, the institutional SAE form will be sent. Incomplete MedWatch/CIOMS forms are acceptable. For Suspected Unexpected Serious Adverse Reactions (SUSARs), a comprehensive narrative must be included.

TESARO/GSK AESI, SAE and Pregnancy Reporting Information
TESARO/GSK's Pharmacovigilance (PV) department<br>Email: OAX37649@gsk.com<br>Fax : +44(0) 208754 7822

7.4 Routine Adverse Event Reporting

Data on all adverse experiences/toxicities regardless of seriousness must be collected for documentation purposes only. Adverse events (AEs) will be collected from the time of informed consent to 30 days after last study drug administration. AEs that occur before the first study drug administration, concomitant illnesses, which existed before study entry, but did not worsen during the treatment period and any pre-existing conditions are known as "baseline symptoms" and by definition are "unrelated" to study drug. Any AEs related to biopsy will be recorded in source and CRFs. All AEs, regardless of the source of identification (e.g., physical examination, laboratory assessment, electrocardiograms [ECG], reported by patient), must be documented.

Clinical Laboratory Abnormalities:

All clinically significant CTCAE v4.0 gradable abnormal laboratory or hematologic events that are ≥ grade 2 will be recorded in source and in study CRFs. These abnormal laboratory results will be followed until the related AE resolves to ≤grade 1 or baseline as per Section 5.4.

Clinically significant laboratory AEs will include, but not be limited to, those that result in any of the following:

- Change in study drug dosing; or
- Administration of a concomitant medication; or
- AE considered clinically significant as per the Study Investigator as documented in their dictation

7.5 Documentation of Adverse Events

All AEs must be captured in the source documents, as well as reported in *electronic document capture (EDC) system*. AEs reported using SAE forms must also be reported in *EDC system*.

All serious and non-serious AEs occurring from informed consent to 30 days after last study drug administration must be recorded in source and CRFs. The Investigator should review all documentation (e.g., hospital progress notes, laboratory, or diagnostic reports) relative to the event being reported.

7.6 Follow-Up of AEs and SAEs

SAEs and AEs should be followed for 30 days after the last dosing of study drug/biologic or until they are resolved (< grade 2 or baseline values), stabilized, or the patient is lost to follow-up and cannot be contacted. Additional investigations (*e.g.*, laboratory tests, diagnostic procedures, or consultation with other healthcare professionals) may be required to completely investigate the nature and/or causality of an AE or SAE. If the patient dies during the study or within 30 days following the last dose of study medication, any postmortem findings (including histopathology) should be provided to the Central Office. CRF data should be updated with any new information as appropriate.

7.7 Pregnancy

Any pregnancy of a study subject or of a study subject's partner that occurs during study participation should be reported for pregnancies that occur up to 30 days after the last dose of study medication. To ensure patient safety each pregnancy must also be reported to TESARO/GSK within 24 hours of learning of its occurrence. The pregnancy should be followed up to determine outcome, including spontaneous or voluntary termination, details of birth, and the presence or absence of any birth defects, congenital abnormalities or maternal and newborn complications.

TESARO/GSK AESI, SAE and Pregnancy Reporting Information
TESARO/GSK's Pharmacovigilance (PV) department<br>Email: OAX37649@gsk.com<br>Fax : +44(0) 208754 7822

7.8 Investigator Notifications/Safety Notifications/Safety Reports

The Principal Investigator and Central Office will receive Investigator Notifications (INs) issued by TESARO/GSK for suspect, unexpected SAEs which have occurred in TESARO/GSK-sponsored studies with the study drug. The Central Office is responsible for forwarding these INs to all sites participating in the study. Sites are then responsible for forwarding these INs to their Research Ethics Boards, according to local practice.

7.9 Reporting Product Quality Complaints for Niraparib and TSR-042

Any written, electronic or oral communication that alleges dissatisfaction related to manufactured clinical drug product with regards to its manufacturing, testing, labeling, packaging, or shipping, must be reported by the Principal Investigator or qualified designee to TESARO/GSK within 1 working day of first becoming aware of the possible defect to TESARO/GSK QA at tesaro.qa@gsk.com.The product and packaging components in question, if available, must be stored in a secure area under specified storage conditions until it is determined whether the product is required to be returned for investigation of the defect. If the product complaint is associated with an SAE, the SAE must be reported separately in accordance with the protocol, and the SAE report should mention the product quality complaint.

7.10 Special Situations: Abuse, Misuse, Medication Errors, Overdose, and Accidental or Occupational Exposure

- **Abuse:** is the persistent or sporadic, intentional excessive use of the study treatment which is accompanied by harmful physical or psychological effects.
- **Misuse:** medicinal product is intentionally and inappropriately used not in accordance with the authorized/approved product information.
- **Medication error:** is any preventable incident that may cause or lead to inappropriate study treatment use or patient harm while the study treatment is in the control of the health care professionals or patients. Such incident may be due to health care professional practice, product labeling, packaging and preparation, procedures for administration, and systems, including the following: prescribing, order communication, nomenclature, compounding, dispensing, distribution, administration, education, monitoring, and use.
- **Overdose:** is a deliberate or accidental administration of study treatment to a study patient, at a dose greater than that which was assigned to that patient per the study protocol and under the direction of the Investigator. If an overdose with a TESARO/GSK product, the Sponsor Institution and TESARO/GSK should be notified immediately, and the patient should be observed closely for AEs. Associated AEs should be treated and monitored by the Investigator. The dosage of study drug administered, any associated AEs, and/or treatment provided to the patient because of the overdose, should be reported.
- **Accidental /Occupational exposure:** is the unintentional exposure to a study treatment as a result of one's professional or non-professional occupation, or accidental exposure to a non-professional to whom exposure was not intended (i.e., study product given to wrong patient).

Reporting Special Situations: All occurrences of abuse, misuse, medication error, overdose, and accidental or occupational exposure associated with a TESARO/GSK product must be reported on a Special Situations Report Form to the Sponsor Institution and to TESARO/GSK within 5 business days of awareness regardless of whether or not an AE or SAE has occurred. If the abuse, misuse, medication error, overdose, or accidental / occupational exposure is associated with an AE, an SAE Report Form must also be submitted to the Sponsor Institution and to TESARO/GSK within 24 hours of awareness.

7.11 Data Safety and Monitoring Board

The Drug Development Program Data Safety and Monitoring Board, an independent group of experts, will be reviewing the data from this research throughout the study to see if there are unexpected or more serious side effects than described in the consent.

8. PHARMACEUTICAL INFORMATION

A list of the adverse events and potential risks associated with the investigational agent administered in this study can be found in Section 7.1.

8.1 Investigational Agent

8.1.1 Niraparib

Niraparib, ([3S]-3-[4-{7-(aminocarbonyl)-2H-indazol-2-yl} phenyl] piperidine [tosylate monohydrate salt]), is an orally available, potent, highly selective PARP-1 and -2 inhibitor. The excipients for niraparib are lactose monohydrate and magnesium stearate.

Investigational agent Manufacturing

Niraparib is manufactured by TESARO/GSK.

Investigational agent Packaging and Labeling

Niraparib is supplied by TESARO/GSK as 100 mg capsules packaged in high-density polyethylene (HDPE) bottles with child-resistant plastic closures. TESARO/GSK provided Study drug will be meet all regulatory requirements and will be compliant with regulations.

Investigational agent Handling and Storage

Investigational agent will be stored under secure (with limited access), and temperature-controlled conditions for the duration of the study. Investigational agent bottles will be stored at ambient temperature (i.e.15-30°C) in an area accessible only to authorized staff. Patients will be asked to store their one-month supply of drug at ambient temperature. Study drug inventory forms will be kept by the Investigator, or designee.

Availability

Niraparib is provided under a Collaborative Agreement between the Sponsor and the agent manufacturer TESARO/GSK.

Investigational Agent Ordering and Shipping

Niraparib will be supplied by TESARO/GSK to each study site. The site Principal Investigator, or authorized study personnel, upon receipt of the study medication supplies, will conduct an inventory and acknowledge receipt to TESARO/GSK, or designee.

Study medication should only be dispensed once a patient has (1) signed an informed consent form (ICF), (2) met all eligibility criteria for entry into the study, (3) completed all screening and continuing eligibility requirements, and (4) been assigned a patient identification number.

Agent Accountability

The investigator, or a responsible party designated by the investigator, must maintain a careful record of the inventory and disposition of all agents received using their site-specific drug accountability log. The drug accountability log includes information including the enrollment number, amount dispensed, and amount returned to the pharmacy, if applicable. Product returned to the pharmacy will be stored under the same conditions as products not yet dispensed but will be marked as 'returned' and kept separate from the products not yet dispensed. All dispensing and accountability records will be available for Sponsor review. When the study monitor visits, he/she will reconcile the drug accountability log with the products stored in the pharmacy. The pharmacist will dispense study drug for each patient according to the protocol and pharmacy manual, if applicable.

8.1.2 TSR-042

TSR-042 is an IgG4 antibody and will be supplied as a solution in vials containing 500 mg (50 mg/ml).

Investigational Agent Manufacturing:

TSR-042 is manufactured by TESARO/GSK.

Investigational Agent Packaging, Labeling and Storage

TSR-042 for injection is supplied in vials containing 500 mg at a concentration of 50 mg/mL.

Investigational Agent Availability

TSR-042 is provided under a Collaborative Agreement between the Sponsor and the agent manufacturer TESARO/GSK.

Investigational Agent Ordering and Shipping

TSR-042 will be supplied by TESARO/GSK to each study site. The site Principal Investigator, or authorized study personnel, upon receipt of the study medication supplies, will conduct an inventory and acknowledge receipt to TESARO/GSK, or designee.

Study medication should only be dispensed once a patient has (1) signed an informed consent form (ICF), (2) met all eligibility criteria for entry into the study, (3) completed all screening and continuing eligibility requirements, and (4) been assigned a patient identification number.

Investigational Agent Accountability

The Investigator or designee is responsible for maintaining accurate dispensing records of the study treatment throughout the clinical study. The study treatment accountability log includes information including a patient identifier, amount and date dispensed, and amount and date returned to the pharmacy, if applicable. Product returned to the pharmacy will be stored under the same conditions as products not yet dispensed but will be marked as 'returned' and kept separate from the products not yet dispensed.

All dispensing and accountability records should be stored in accordance to the Sponsor institution regulations. The pharmacist will dispense study treatment for each participant according to the protocol and storage and handling manual, if applicable.

9. BIOMARKER, CORRELATIVE, AND SPECIAL STUDIES

9.1 Biomarker Studies

Correlative studies evaluating tumor biology relevant to endometrial cancer will focus on (1) evaluating baseline status of PTEN (using standard IHC to identify PTEN loss), (2) tumor MSI status using a custom IHC panel, (3) analysis of *BRCA1/2* and other HRD genes, and (4) genomic sequencing, and immune infiltration. All Correlative Studies will be conducted following developed standard operating procedures, for both sample acquisition and performing individual assays.

Patient who have undergone molecular profiling through different programs, including VENUS (Molecular and Immunological characterization of gynecological malignancies, NCT03420118), and OCTANE (Ontario-wide Cancer TArgeted Nucleic Acid Evaluation, NCT02906943), the molecular data will be recorded as part of this current trial.

9.2 Laboratory Correlative Studies

9.2.1 <u>Collection of Specimen(s)</u>

Paraffin embedded archival blocks are preferred, however if blocks cannot be released 25 unstained slides cut at 4 microns thick each mounted on positively charged (for IHC) or uncharged (sequencing) slides are also acceptable. All correlative analyses will be performed in the archival specimens of all patients.

9.2.2 PTEN Immunohistochemistry Analysis

An optimized PTEN antibody will be used and immunohistochemistry (IHC) analysis will be carried out on the Ventana Benchmark XT autostainer using the Ultra-view detection system for PTEN at the Applied Molecular Profiling Laboratory or on another system if employed at our institute at the time of analysis. Importantly, PTEN immunostaining has a built-in control on each section as the tumor stromal fibroblasts and endothelial cells show strongly positive (2+) staining. Positive staining is defined as >90%. Loss of PTEN expression is defined as <1% cytoplasmic staining in tumor cells. Intermediate levels of staining are scored as heterogeneous pattern [96]. For the purpose of analysis, cases with negative and heterogenous staining are considered to have PTEN loss, whereas positive cases PTEN retained [105].

9.2.3 MxIF Analysis

4 µm slides will be made from formalin-fixed and paraffin-embedded archival tumor tissues (or fresh tumor biopsies, when archival tissue is unavailable) for multiplex immunofluorescence (MxIF). Tissues will be stained using a MxIF platform developed by the General Electric Global Research Center (GE-GRC; New York) and adapted at the Sunnybrook Research Institute's Biomarker Imaging Research Lab (BIRL; Toronto). Tissues will be examined by MxIF for markers of various immune cell populations including: T cells (CD3, CD4, CD8), B cells (CD20), NK cells (CD56), epithelial cells (cytokeratin), myeloid cells (CD68), as well as for the expression of immune checkpoint markers (PD-1, PD-L1). MxIF images will be analyzed using software developed by the GE-GRC, which supports single-cell phenotyping using additional cell compartment segmentation markers: DAPI, ribosomal S6, and NaKATPase. Altogether, MxIF analysis of the tumor microenvironment in these patient samples will allow us to characterize the spatial distribution of infiltrating immune cells and correlate these data with clinical outcomes. Immunohistochemistry can be also performed for the immune markers.

9.2.4 MSI analysis

Archival paraffin slides will be stained immunohistochemically using primary antibodies to evaluate MSI status. These archived samples will then be evaluated by the specific companion diagnostic test at the Applied Molecular Profiling Laboratory. MSI testing will be performed in samples with >70% tumor cells, as determined by examination of a representative hematoxylin and eosin-stained section of the tumor. IHC staining using anti-MLH1, anti-MSH2, anti-MSH6, and anti-PMS2 will be performed on archival tumor samples. Loss of mismatch-repair protein expression will be defined by lack of nuclear staining in the lesional tissue. MSH-2 testing will be carried out on the Ventana Benchmark XT autostainer (already optimized, clone G219-1129, cat. no 760-4265, or equivalent if other antibodies are used in the future). All antibodies will be optimized for immunohistochemistry using the Ventana autostainer using positive/negative tissue

specimens at the Applied Molecular Profiling Laboratory. The analysis will be performed by an expert pathologist. MSI /MMR (microsatellite instability MSI / mismatch repair protein –MMR) will be collected for all Cohort II patients from previous pathology report.

9.2.5 HRD status

A screening panel of several genes involved in cell cycle regulation, DNA replication, DNA recombination and repair pathways will be developed. The analyses will be performed with next generation sequencing (NGS) for mutations in HRD genes and/or IHC for expression levels. Alternatively, the Myriad myChoice HRD test maybe employed to determine tumor HRD status.

9.2.6 Genomic analysis

Next Generation Sequencing technology will be used to profile tumor DNA to correlate genomic alterations with response. We propose targeted panel whole-exonic sequencing (UHN Advanced Molecular Diagnostics Lab - AMDL, CAP/CLIA; S. Kamel-Reid) or whole exome (or genome) sequencing (Genomics Program – Dr. Trevor Pugh), after examination of all slides available, the most suitable, representative blocks will be chosen. Where archival FFPE samples are utilized, blocks are preferred though 7 micron thick sections on 15-20 unstained slides and one H&E slide are acceptable. Biopsy samples will be assessed for quality and quantity of malignant cells. DNA will be extracted and checked for quality prior to genomic analysis.

9.2.7 Shipping of Specimen(s)

Archival samples and formalin-fixed samples should be sent at ambient temperature. Both kinds of specimens should be shipped to the Correlative Studies Program at the Princess Margaret Cancer Centre (See below). Samples and inventory sheet must be shipped by overnight delivery in designated shipping container. Shipment must be scheduled for a Monday, Tuesday or Wednesday only.

Archival tumor specimens should be shipped to:
Correlative Studies Program
Princess Margaret Cancer Centre
610 University Ave 7-420
Toronto, On
M5G 2M9
Phone: 416-946-4501 ext 5047
Fax: 416-946-4431

9.2.8 Site(s) Performing Correlative Studies:

Applied Molecular Profiling Laboratory (AMPL)/Drug Development Biomarker Laboratory (DDBL), Princess Margaret Cancer Centre

Department of Pathology – Immunohistochemistry Laboratory, Laboratory Medicine Program, Toronto General Hospital, University Health Network

Advanced Molecular Diagnostics Lab - AMDL, CAP/CLIA, and Genomics Programme

Princess Margaret Cancer Centre – OICR Translational Genomics Laboratory

Biomarker Imaging Research Lab (BIRL) – Sunnybrook Research Institute.

10. STUDY CALENDAR

Baseline (pre-study) evaluations are to be conducted within 28 days prior to start of protocol therapy, unless specified differently in the study calendar. Scans and x-rays must be done ≤28 days prior to the start of therapy. In the event that the patient's condition is deteriorating, laboratory evaluations should be repeated within 48 hours prior to initiation of the next cycle of therapy. The following schedule of assessments applies to all subjects. More frequent assessments should be obtained if clinically indicated. If pre-study assessments were done within 3 days prior to treatment initiation, they do not need to be repeated for cycle 1 day 1. Scheduled study visits are allowed to take place within 3 days of scheduled visit, unless otherwise specified.

10.1 Cohort I

A cycle is 28 (± 3) days long. Radiological assessment for tumor RECIST measurements is required at the end of every 2 cycles (8 weeks with a window of ± 7 days from the date of previous scan) through Cycle 12 (48 weeks), then at the end of every 3 cycles (12 weeks ± 7 days from the date of previous scan).
Niraparib will be administered on a continuous basis.

	Pre-Study	Day -7[6]	Cycle 1				Cycle 2		Cycle n	End of Study	30 Day Follow-up[12]	Follow-up[8]
			Day 1	Day 8	Day 15	Day 22	Day 1	Day 15	Day 1			
Niraparib[1]			X--X									
Informed consent	X											
Demographics	X											
Medical history	X											
Physical exam	X		X		X		X		X	X	X	
Vital signs[2]	X		X		X		X	X	X	X		
ECOG performance status	X		X		X		X	X	X	X	X	
Height	X											
Weight	X		X				X			X		
CBC with differentials	X	X	X	X	X	X	X	X	X	X	X	
Serum chemistry[3]	X	X	X		X		X		X	X	X	
PT or INR, APTT[9]	X									X		
MDS/AML testing [4]	X									X		
12 lead ECG[9]	X						X					
B-HCG[5]	X											
Adverse event evaluation	X		X--X								X	
Concurrent medications	X		X--X								X	
Radiological Evaluation[10]	X		Radiologic measurements should be performed every 8 weeks[7] for the first 12 cycles then every 12 weeks to progression.							X[11]		X

Tumor assessment	X		Tumor measurements are repeated every 8 weeks[7] for the first 12 cycles then every 12 weeks to progression. Documentation (radiologic) must be provided for patients removed from study for objective progression.	X[11]		X
Archival or Biopsy tumor tissue	X					

ECG = electrocardiogram; ECOG = Eastern Cooperative Oncology Group
[1]Niraparib dose as assigned; oral administration, continuous for 28 days, taken at the same time once every day
[2]Vital signs include blood pressure, temperature, pulse, O2 saturation, and respiratory rate.
If dose interruption or modification is required at any point on study because of hematologic toxicity, weekly blood draws for CBC will be monitored until the event has resolved to the specified levels, after which monitoring every 4 weeks may resume
[3]Chemistry panel includes albumin, alkaline phosphatase, ALT, amylase, AST, bicarbonate, blood urea nitrogen, calcium, chloride, creatinine, glucose, lipase, magnesium, phosphorus, potassium, sodium, total bilirubin, total protein
[4]Patients will have blood samples collected at baseline, to allow cytogenetic assessment in the event a patient later develops MDS/AML. If a subject is suspected to have developed MDS/AML either on-study or after end of treatment, an additional 6mL sample is taken to be analyzed together with the archived samples above in order to assess changes in the molecular signature, to determine whether MDS/AML developed during treatment with the PARP inhibitor.
[5]Serum pregnancy test (women of childbearing potential)
[6]CBC and Serum Chemistry at screening must be conducted within 7 days prior to study drug administration.
[7]Window of ± 7 days for radiological assessment
[8]Refer to Section 5.4 regarding details on follow-up procedures. For patients who come off study treatment without disease progression, radiological assessment is required every 12 weeks ± 7 days. Follow up for an adverse event is weekly in the first 4 weeks and then monthly, until resolution of the adverse event.
[9]Perform as per study calendar and as clinically indicated.
[10]For radiologic assessments by CT, imaging of chest/abdomen/pelvis (CAP) is expected.
[11]Not required if patient has come off treatment due to objective disease progression as per RECIST1.1 (see Section 11.1).
[12]30 days from end of study drug administration.

10.2 Cohort II

A cycle is 21 (± 3) days long. Radiological assessment for tumor RECIST measurements is required every 8 weeks with a window of ± 7 days from the date of previous scan. From 48 weeks on, the scans will be performed at 12 weeks ± 7 days from the date of previous scan.
Niraparib will be administered on a continuous daily basis until disease progression or unacceptable toxicity. From cycle 1 to 4 TSR-042 will be administered at 500 mg every cycle (every 3 weeks), followed by 1,000 mg every 2 cycles (every 6 weeks). For a maximum of 2 years.

	Pre - Study	Day -7[6]	Cycle 1			Cycle 2		Cycle 3-4	Cycle 5	Cycle 6	Cycle N	End of Study	30 Day Follow-up[12]	Follow Up8
			Day 1	Day 8	Day 15	Day 1	Day 15	Day 1	Day 1	Day 1	Day 1			
Niraparib[1]			X--X											
TSR-042[2]			X (500mg)			X (500mg)		X (500mg)	X (1000mg)		X (1000mg)			
Informed consent	X													
Demographics	X													
Medical history	X													
Physical exam	X		X		X	X		X	X	X	X	X	X	
Vital signs[3]	X		X[13]		X	X[13]		X[13]	X	X	X[13]	X		
ECOG performance status	X		X		X	X	X	X	X	X	X		X	
Height	X													
Weight	X		X			X								
CBC with differentials	X[6]	X	X	X	X	X	X	X	X	X	X	X	X	

Serum chemistry[4]	X[6]	X	X		X	X	X	X	X	X	X	X	X	
PT or INR, APTT	X											X		
12 lead ECG[9]	X					X								
B-HCG[5]	X													
Adverse event evaluation	X		X--X										X	
Concurrent medications	X		X--X										X	
Radiological Evaluation[10]	X		Radiologic measurements should be performed every 8 weeks[7] for the first 12 cycles then every 12 weeks to progression									X[11]		X
Tumor assessment	X		Tumor measurements are repeated every 8 weeks[7] for the first 12 cycles then every 12 weeks to progression. Documentation (radiologic) must be provided for patients removed from study for objective progression.											X
Archival or Biopsy tumor tissue	X													

ECG = electrocardiogram; ECOG = Eastern Cooperative Oncology Group

[1]Niraparib dose as assigned; oral administration, continuous for 21 days, taken at the same time once every day

[2]TSR-042 will be administered at 500mg every 3 weeks on cycles 1 to 4, and subsequently at 1000mg every 6 weeks on cycle 5 onwards. TSR-042 will be administered for a maximum of 2 years.

[3]Vital signs include blood pressure, temperature, pulse, O2 saturation, and respiratory rate.

[4]Chemistry panel includes albumin, alkaline phosphatase, ALT, amylase, AST, bicarbonate, blood urea nitrogen, calcium, chloride, creatinine, glucose, lipase, magnesium, phosphorus, potassium, sodium, total bilirubin, total protein

[5]Serum pregnancy test (women of childbearing potential)

[6]CBC and Serum Chemistry at screening must be conducted within 7 days prior to study drug administration.

[7]Window of ± 7 days for radiological assessment

[8]Refer to Section 5.4 regarding details on follow-up procedures. For patients who come off study treatment without disease progression, radiological assessment is required every 12 weeks ± 7 days. Follow up for an adverse event is weekly in the first 4 weeks and then monthly, until resolution of the adverse event.

[9]Perform as per study calendar and as clinically indicated.

[10]For radiologic assessments by CT, imaging of chest/abdomen/pelvis (CAP) is expected.

[11]Not required if patient has come off treatment due to objective disease progression as per RECIST1.1 (see Section 11.1).

[12]30 days from end of study drug administration.

[13] Day1 Monitoring pre and post infusion for all cycles will be done per section 5.1 Treatment plan cohort II (Vital sign monitoring during infusion of TSR-042)

Note-If dose interruption or modification is required at any point on study because of hematologic toxicity, weekly blood draws for CBC will be monitored until the event has resolved to the specified levels, after which monitoring every 4 weeks may resume

10.3 MDS/AML Testing

Patients will be assessed for MDS/AML regularly using CBC with differential tests at each visit and if any patient is diagnosed with MDS/AML while on study or after the end of treatment, a bone marrow aspirate/biopsy must be completed by a local hematologist. Testing completed as part of standard of care is sufficient as long as the site receives the local hematologist's report, which must include a classification according to World Health Organization criteria. The site must keep a copy of the report with the patient's study file.

11. MEASUREMENT OF EFFECT

11.1 Antitumor Effect – Solid Tumors

For the purposes of this study, patients should be evaluated for response every 8 weeks (±7 days) for 48 weeks, then every 12 weeks (±7 days) until progression. Frequency of scans will not change despite any treatment holds or delays.

Response and progression will be evaluated in this study using the international criteria proposed by the revised Response Evaluation Criteria in Solid Tumors (RECIST) guideline (version 1.1) [106]. Changes in the largest diameter (uni-dimensional measurement) of the tumor lesions and the shortest diameter in the case of malignant lymph nodes are used in the RECIST criteria.

11.1.1 Definitions

Evaluable for toxicity. All patients will be evaluable for toxicity from the time of their first treatment with study treatment.

Evaluable for objective response. Only those patients who have measurable disease present at baseline, have received at least one cycle of therapy, and have had their disease re-evaluated will be considered evaluable for response. These patients will have their response classified according to the definitions stated below. (Note: Patients who exhibit objective disease progression prior to the end of cycle 1 will also be considered evaluable.)

Evaluable Non-Target Disease Response. Patients who have lesions present at baseline that are evaluable but do not meet the definitions of measurable disease, have received at least one cycle of therapy, and have had their disease re-evaluated will be considered evaluable for non-target disease. The response assessment is based on the presence, absence, or unequivocal progression of the lesions.

11.1.2 Disease Parameters

Measurable disease. Measurable lesions are defined as those that can be accurately measured in at least one dimension (longest diameter to be recorded) as ≥20 mm by chest x-ray or as ≥10 mm with CT scan, MRI, or calipers by clinical exam. All tumor measurements must be recorded in millimeters (or decimal fractions of centimeters).

Note: Tumor lesions that are situated in a previously irradiated is only considered measurable if there has been progression in that lesion at study entry.

Malignant lymph nodes. To be considered pathologically enlarged and measurable, a lymph node must be ≥15 mm in short axis when assessed by CT scan (CT scan slice thickness recommended to be no greater than 5 mm). At baseline and in follow-up, only the short axis will be measured and followed.

Non-measurable disease. All other lesions (or sites of disease), including small lesions (longest diameter < 10 mm or pathological lymph nodes with ≥ 10 to < 15 mm short axis) as well as truly non-measurable lesions. Lesions considered truly non-measurable include: leptomeningeal disease, ascites, pleural/pericardial effusions, lymphangitic involvement of skin or lung, inflammatory breast disease, abdominal masses/abdominal organomegaly identified by physical exam that is not measurable by reproducible imaging techniques.

Note: Cystic lesions that meet the criteria for radiographically defined simple cysts should not be considered as malignant lesions (neither measurable nor non-measurable) since they are, by definition, simple cysts.

'Cystic lesions' thought to represent cystic metastases can be considered as measurable lesions, if they meet the definition of measurability described above. However, if non-cystic lesions are present in the same patient, these are preferred for selection as target lesions.

Target lesions. All measurable lesions up to a maximum of 2 lesions per organ and 5 lesions in total, representative of all involved organs, should be identified as **target lesions** and recorded and measured at baseline. Target lesions should be selected on the basis of their size (lesions with the longest diameter), be representative of all involved organs, but in addition should be those that lend themselves to reproducible repeated measurements. It may be the case that, on occasion, the largest lesion does not lend itself to reproducible measurement in which circumstance the next largest lesion which can be measured reproducibly should be selected. A sum of the diameters (longest for non-nodal lesions, short axis for nodal lesions) for all target lesions will be calculated and reported as the baseline sum diameters. If lymph nodes are to be included in the sum, then only the short axis is added into the sum. The baseline sum diameters will be used as reference to further characterize any objective tumor regression in the measurable dimension of the disease.

Non-target lesions. All other lesions (or sites of disease) including any measurable lesions over and above the 5 target lesions should be identified as **non-target lesions** and should also be recorded at baseline. Measurements of these lesions are not required, but the presence, absence, or in rare cases unequivocal progression of each should be noted throughout follow-up.

11.1.3 Methods for Evaluation of Measurable Disease

All measurements should be taken and recorded in metric notation using a ruler or calipers. The same method of assessment and the same technique should be used to characterize each identified and reported lesion at baseline and during follow-up.

Clinical Lesions: Clinical lesions will only be considered measurable when they are superficial (e.g., skin nodules and palpable lymph nodes) and ≥ 10 mm diameter as assessed using calipers (e.g. skin nodules). For the case of skin lesions, documentation by color photography including a ruler to estimate the size of the lesion is recommended. When lesions can be evaluated by both clinical exam and imaging, imaging evaluation should be undertaken since it is more objective and may also be reviewed at the end of the study.

Chest X-ray: Lesions on chest X-ray are acceptable as measurable lesions when they are clearly defined and surrounded by aerated lung. However, CT is preferable.

CT & MRI: CT is the best currently available and reproducible method to measure lesions selected for response assessment. CT should be performed with slice thickness of 5 mm or less. When CT scans have slice thickness greater than 5 mm, the minimum size for a measurable lesion should be twice the slice thickness. MRI is also acceptable in certain situations (e.g. for body scans).

Ultrasound: Ultrasound is not useful in assessment of lesion size and should not be used as a method of measurement. Ultrasound examinations cannot be reproduced in their entirety for independent review at a later date and, because they are operator dependent, it cannot be guaranteed that the same technique and measurements will be taken from one assessment to the next. If new lesions are identified by ultrasound in the course of the study, confirmation by CT or MRI is advised.

Cytology, Histology: These techniques can be used to differentiate between PR and CR in rare cases. The cytological confirmation of the neoplastic origin of any effusion that appears or worsens during treatment can be considered if the measurable tumor has met criteria for response or stable disease in order to differentiate between response (or stable disease) and progressive disease.

11.1.4 Response Criteria

11.1.4.1 Evaluation of Target Lesions

Complete Response (CR): Disappearance of all target lesions. Any pathological lymph nodes (whether target or non-target) must have reduction in short axis to <10 mm.

Partial Response (PR): At least a 30% decrease in the sum of the diameters of target lesions, taking as reference the baseline sum diameters.

Progressive Disease (PD): At least a 20% increase in the sum of the diameters of target lesions, taking as reference the smallest sum on study (this includes the baseline sum if that is the smallest on study). In addition to the relative increase of 20%, the sum must also demonstrate an absolute increase of at least 5 mm. (Note: the appearance of one or more new lesions is also considered progression).

Stable Disease (SD): Neither sufficient shrinkage to qualify for PR nor sufficient increase to qualify for PD, taking as reference the smallest sum diameters while on study (this includes the

baseline sum if that is the smallest on the study). Note that overall response will not go from PR to SD.

11.1.4.2 Evaluation of Non-Target Lesions

Complete Response (CR): Disappearance of all non-target lesions and normalization of tumor marker level. All lymph nodes must be non-pathological in size (<10 mm short axis).

Note: If tumor markers are initially above the upper normal limit, they must normalize for a patient to be considered in complete clinical response.

Non-CR/Non-PD: Persistence of one or more non-target lesion(s) and/or maintenance of tumor marker level above the normal limits.

Progressive Disease (PD): Appearance of one or more new lesions and/or *unequivocal progression* of existing non-target lesions. *Unequivocal progression* should not normally trump target lesion status. It must be representative of overall disease status change, not a single lesion increase.

Although a clear progression of "non-target" lesions only is exceptional, the opinion of the treating physician should prevail in such circumstances, and the progression status should be confirmed at a later time by the review panel (or Principal Investigator).

11.1.5 Duration of Response

Duration of overall response: The duration of overall response is measured from the time measurement criteria are met for CR or PR (whichever is first recorded) until the first date that recurrent or progressive disease is objectively documented (taking as reference for progressive disease the smallest measurements recorded since the treatment started).

The duration of overall CR is measured from the time measurement criteria are first met for CR until the first date that progressive disease is objectively documented.

Duration of stable disease: Stable disease is measured from the start of the treatment until the criteria for progression are met, taking as reference the smallest measurements recorded since the treatment started, including the baseline measurements.

11.1.6 Overall Response Rate

Overall response rate (ORR) is defined as the proportion of subjects in the analysis population who have complete response (CR) or partial response (PR) at any time during the study using RECIST 1.1 criteria.

11.1.7 Progression-Free Survival

Progression free survival (PFS) is defined as the duration of time from start of treatment to time of progression or death, whichever occurs first. If such an event is not observed after 6 months of follow up, patients are censored.

11.1.8 Overall Survival

Overall survival time (OS) is defined as the time of registration to the date of death by any cause. Following the treatment discontinuation visit, survival status will be collected for all patients using acceptable means including telephone contact.

11.1.9 Clinical Benefit Rate

Clinical benefit rate (CBR) is defined as the proportion of patients with prolonged stable disease (SD ≥ 16 weeks), complete or partial response by RECIST 1.1 criteria.

12. DATA REPORTING / REGULATORY REQUIREMENTS

Adverse event lists, guidelines, and instructions for AE reporting can be found in Section 7.0 (Adverse Events: List and Reporting Requirements).

12.1 Data Collection and Reporting

All data obtained in the clinical trial described in this protocol will be reported on eCRFs in the Medidata Electronic Document Capture system (Medidata). Data reported on eCRFs should be consistent with the source documents and verifiable. All data for the primary and secondary endpoints will source verified prior to publication. The Investigator will review the data and electronically sign the eCRFs to acknowledge agreement with the data entered. Data entered into Medidata will be used for developing tables and listings for the final study report.

Prior to the site activation, the Investigator will sign off on a completed Site Participants Log showing the signatures and handwritten initials of all individuals who are authorized to make or change entries on source documents and eCRFs.

12.2 Source Documents

Source documents refer to the original documents, data, and records where the first recording of a data point occurred. Examples of source documentation include, but are not limited to:

> Hospital records, clinical and office charts, laboratory notes, memoranda, subjects' diaries or evaluation checklists, pharmacy dispensing records, recorded data from automated instruments, copies or transcriptions certified after verification as being accurate copies, microfiches, photographic negatives, microfilm or magnetic media, x-rays, subject files, and records kept at the pharmacy, at the laboratories and at medico-technical departments involved in the clinical trial)

Please ensure that source document entries are attributable, legible, contemporaneous, original, and accurate. Note that sign-off of source documents should be attributable to a single record and "bracketing" multiple entries on source document pages for a single signature is not allowed. Corrections to source document entries should only be completed by drawing a single line through the previous entry and then recording the corrected data, initialing the change, and dating the change. Only the individual that initially recorded the data should make any corrections.

12.3 Retention of Patient Records and Study Files

The ICH guidance document, Good Clinical Practice: Consolidated Guidelines (ICH Guidance Document E6) (1997) states that the investigator and sponsor shall retain study records relating to the study until at least 2 years after the last approval of a marketing application and until there are no pending or contemplated marketing applications, or at least 2 years have elapsed since the formal discontinuation of clinical development of the investigational product. In the event of a trial discontinuation, sponsor records should also be kept for a minimum of 2 years. Per Health Canada, all original records should be maintained for 25 years after the above requirements are satisfied and the final report has been issued. Records contained in the Clinical Trial Application should be maintained on file for at least 25 years. We will comply with these regulations. The Sponsor will notify sites when documents are to be destroyed.

12.4 Site and Study Closure

Upon completion of the study, the following activities, when applicable, will be completed by the Central Office in conjunction with the Investigator, as appropriate:

- Collection of study materials (i.e., specimen collection kits, drug shippers, etc.)
- Data clarifications and/or resolutions
- Accounting, reconciliation, and final disposition of used and unused study medication
- Review of site study records for completeness

If the Principal Investigator or appropriate regulatory officials identify conditions arising during the study that indicate that the study should be halted or that the study center should be terminated, this action may be taken after appropriate consultation among the Sponsor and Investigator. Conditions that may warrant termination of the study include, but are not limited to, the following:

- The discovery of an unexpected, serious, or unacceptable risk to the patients enrolled in the study
- A decision on the part of the Sponsor to suspend or discontinue testing, evaluation, or development of the product
- Failure of the Investigator to enroll patients into the study at an acceptable rate
- Failure of the Investigator to comply with pertinent regulations of appropriate regulatory authorities
- Submission of knowingly false information to the Sponsor, or appropriate regulatory authority
- Insufficient adherence to protocol requirements

- Refusal of the Investigator to supply source documentation of work performed in this clinical trial

Study termination and follow-up will be performed in compliance with the conditions set forth in the International Conference on Harmonisation (ICH) sixth efficacy publication (E6) on Good Clinical Practice, Section 4.12, ICH E6 4.13, ICH E6 5.20, and ICH E6 5.21.

13. STATISTICAL CONSIDERATIONS

13.1 Study Design/Endpoints

The Simon two-stage design is employed. With the null hypothesis that Clinical benefit rate (CBR), $p \leq 0.10$ versus the alternative that $p \geq 0.35$ and setting alpha=beta=0.10, Stage I has a planned accrual of 10 patients. If at least 1 clinical benefit instance is observed at the end of stage I, the study will proceed to stage II with 12 additional patients to be accrued for the total of 22 patients. If at least 5 instances of clinical benefit are observed among the 22 patients, this agent would be considered worthy of further investigation.
The study will enroll patients with recurrent endometrial cancer unselected for PTEN status. Mandatory archival tissue is requested for retrospective analysis.

After the enrollment of a total of 22 patients in cohort I (niraparib alone), patients will be registered in cohort II with the combination of niraparib and TSR-042. A total of 22 patients will be enrolled in cohort II (combination niraparib/TSR-042). If at least 5 instances of clinical benefit are observed among the 22 patients, this agent would be considered worthy of further investigation.

If the clinical benefit rate does not reach the pre-defined level (positive ≥5/22 overall) after stage II in cohort I, PTEN analysis will be performed and the study will be considered to expand to PTEN-loss subgroup if this subgroup's CBR is not worse than the rest of the patients initially in cohort 1 with niraparib single agent. The same criteria as calculated by the design above (≥1/10 CBR go to stage II and positive ≥5/22 CBR overall) will be used.

13.2 Sample Size/Accrual Rate

The accrual in cohort I, stage I and II is expected to be 22 patients. The accrual in cohort II is expected to be 22 patients.

13.3 Analysis of Secondary and Exploratory Endpoints

Secondary endpoints to be evaluated include:

- To assess the safety and tolerability of single agent niraparib and the combination of niraparib and TSR-042 in the indicated target population.
- To determine overall response rate, duration of response, progression free survival (PFS) and overall survival (OS) of niraparib and the combination of niraparib and TSR-042 in recurrent endometrial cancer.

Potential predictors of clinical outcomes will be investigated using logistic regression, Chi-square or Fisher's Exact test as appropriate. Descriptive statistics and plotting of data will also be used to better understand potential relations.
Frequency and severity of adverse events will be tabulated using counts and proportions. 95% confidence interval may be reported.
Overall response rate will be reported with its 95% confidence interval. PFS and OS will be analyzed using Kaplan-Meier method.

Exploratory endpoints to be evaluated include:

- To assess whether the presence PTEN deficiency in archival tumor is predictive of response to treatment
- To explore whether the presence of MSI status is predictive of clinical benefit and response rate to the treatment of recurrent endometrial cancer.
- To assess whether HRD and immune infiltration is predictive of benefit to the treatment; and to examine for a relationship between HRD, PTEN and MSI, and presence of immune markers.

Logistic regression, Chi-square or Fisher's Exact test will be used to explore this association as appropriate.

REFERENCES

1. *American Cancer Society: Cancer Facts and Figures* 2011, American Cancer Society: Atlanta, GA.
2. *Canadian Cancer Society's Steering Committee on Cancer Statistics*. 2011, Toronto, ON: Canadian Cancer Society.
3. Key, T.J. and M.C. Pike, *The dose-effect relationship between 'unopposed' oestrogens and endometrial mitotic rate: its central role in explaining and predicting endometrial cancer risk.* Br J Cancer, 1988. **57**(2): p. 205-12.
4. Kaaks, R., A. Lukanova, and M.S. Kurzer, *Obesity, endogenous hormones, and endometrial cancer risk: a synthetic review.* Cancer Epidemiol Biomarkers Prev, 2002. **11**(12): p. 1531-43.
5. Renehan, A.G., et al., *Body-mass index and incidence of cancer: a systematic review and meta-analysis of prospective observational studies.* Lancet, 2008. **371**(9612): p. 569-78.
6. Amant, F., et al., *Endometrial cancer.* Lancet, 2005. **366**(9484): p. 491-505.
7. Creasman, W.T., et al., *Carcinoma of the corpus uteri. FIGO 26th Annual Report on the Results of Treatment in Gynecological Cancer.* Int J Gynaecol Obstet, 2006. **95 Suppl 1**: p. S105-43.
8. Wright, J.D., et al., *Contemporary management of endometrial cancer.* Lancet, 2012. **379**(9823): p. 1352-60.
9. Obel, J.C., G. Friberg, and G.F. Fleming, *Chemotherapy in endometrial cancer.* Clin Adv Hematol Oncol, 2006. **4**(6): p. 459-68.
10. Hogberg, T., *What is the role of chemotherapy in endometrial cancer?* Curr Oncol Rep, 2011. **13**(6): p. 433-41.
11. Bokhman, J.V., *Two pathogenetic types of endometrial carcinoma.* Gynecol Oncol, 1983. **15**(1): p. 10-7.
12. Brinton, L.A., et al., *Etiologic heterogeneity in endometrial cancer: evidence from a Gynecologic Oncology Group trial.* Gynecol Oncol, 2013. **129**(2): p. 277-84.
13. Zannoni, G.F., et al., *Does high-grade endometrioid carcinoma (grade 3 FIGO) belong to type I or type II endometrial cancer? A clinical-pathological and immunohistochemical study.* Virchows Arch, 2010. **457**(1): p. 27-34.
14. Cancer Genome Atlas Research, N., et al., *Integrated genomic characterization of endometrial carcinoma.* Nature, 2013. **497**(7447): p. 67-73.
15. Byron, S.A. and P.M. Pollock, *FGFR2 as a molecular target in endometrial cancer.* Future Oncol, 2009. **5**(1): p. 27-32.
16. Kuhn, E., et al., *Molecular characterization of undifferentiated carcinoma associated with endometrioid carcinoma.* Am J Surg Pathol, 2014. **38**(5): p. 660-5.
17. Fadare, O., et al., *The clinicopathologic significance of p53 and BAF-250a (ARID1A) expression in clear cell carcinoma of the endometrium.* Mod Pathol, 2013. **26**(8): p. 1101-10.
18. Hoang, L.N., et al., *Immunohistochemical characterization of prototypical endometrial clear cell carcinoma--diagnostic utility of HNF-1beta and oestrogen receptor.* Histopathology, 2014. **64**(4): p. 585-96.
19. Zhang, Z.M., et al., *The clinicopathologic significance of the loss of BAF250a (ARID1A) expression in endometrial carcinoma.* Int J Gynecol Cancer, 2014. **24**(3): p. 534-40.
20. Hussein, Y.R., et al., *Clinicopathological analysis of endometrial carcinomas harboring*

somatic POLE exonuclease domain mutations. Mod Pathol, 2015. **28**(4): p. 505-14.

21. Esteller, M., et al., *MLH1 promoter hypermethylation is associated with the microsatellite instability phenotype in sporadic endometrial carcinomas.* Oncogene, 1998. **17**(18): p. 2413-7.
22. Modica, I., et al., *Utility of immunohistochemistry in predicting microsatellite instability in endometrial carcinoma.* Am J Surg Pathol, 2007. **31**(5): p. 744-51.
23. Mei, Z., et al., *Tumour-infiltrating inflammation and prognosis in colorectal cancer: systematic review and meta-analysis.* Br J Cancer, 2014. **110**(6): p. 1595-605.
24. Salgado, R., et al., *Harmonization of the evaluation of tumor infiltrating lymphocytes (TILs) in breast cancer: recommendations by an international TILs-working group 2014.* Annals of oncology : official journal of the European Society for Medical Oncology / ESMO, 2014. **Sep 11**.
25. Schatton, T., et al., *Tumor-infiltrating lymphocytes and their significance in melanoma prognosis.* Methods Mol Biol, 2014. **1102**: p. 287-324.
26. Gooden, M.J., et al., *The prognostic influence of tumour-infiltrating lymphocytes in cancer: a systematic review with meta-analysis.* Br J Cancer, 2011. **105**(1): p. 93-103.
27. Schreiber, R.D., L.J. Old, and M.J. Smyth, *Cancer immunoediting: integrating immunity's roles in cancer suppression and promotion.* Science, 2011. **331**(6024): p. 1565-70.
28. Bremnes, R.M., et al., *The role of tumor-infiltrating immune cells and chronic inflammation at the tumor site on cancer development, progression, and prognosis: emphasis on non-small cell lung cancer.* J Thorac Oncol, 2011. **6**(4): p. 824-33.
29. Talmadge, J.E., *Immune cell infiltration of primary and metastatic lesions: mechanisms and clinical impact.* Semin Cancer Biol, 2011. **21**(2): p. 131-8.
30. Shirabe, K., et al., *Tumor-infiltrating lymphocytes and hepatocellular carcinoma: pathology and clinical management.* Int J Clin Oncol, 2010. **15**(6): p. 552-8.
31. Nosho, K., et al., *Tumour-infiltrating T-cell subsets, molecular changes in colorectal cancer, and prognosis: cohort study and literature review.* J Pathol, 2010. **222**(4): p. 350-66.
32. Bellati, F., et al., *Immunology of gynecologic neoplasms: analysis of the prognostic significance of the immune status.* Curr Cancer Drug Targets, 2009. **9**(4): p. 541-65.
33. Oble, D.A., et al., *Focus on TILs: prognostic significance of tumor infiltrating lymphocytes in human melanoma.* Cancer Immun, 2009. **9**: p. 3.
34. Uppaluri, R., G.P. Dunn, and J.S. Lewis, Jr., *Focus on TILs: prognostic significance of tumor infiltrating lymphocytes in head and neck cancers.* Cancer Immun, 2008. **8**: p. 16.
35. Dunn, G.P., I.F. Dunn, and W.T. Curry, *Focus on TILs: Prognostic significance of tumor infiltrating lymphocytes in human glioma.* Cancer Immun, 2007. **7**: p. 12.
36. Chang, W.J., et al., *Inflammation-related factors predicting prognosis of gastric cancer.* World J Gastroenterol, 2014. **20**(16): p. 4586-96.
37. Preston, C.C., et al., *The ratios of CD8+ T cells to CD4+CD25+ FOXP3+ and FOXP3- T cells correlate with poor clinical outcome in human serous ovarian cancer.* PLoS One, 2013. **8**(11): p. e80063.
38. Yoon, H.H., et al., *Prognostic impact of FoxP3+ regulatory T cells in relation to CD8+ T lymphocyte density in human colon carcinomas.* PLoS One, 2012. **7**(8): p. e42274.
39. Kim, S.T., et al., *Tumor-infiltrating lymphocytes, tumor characteristics, and recurrence in patients with early breast cancer.* Am J Clin Oncol, 2013. **36**(3): p. 224-31.

40. Mathai, A.M., et al., *Role of Foxp3-positive tumor-infiltrating lymphocytes in the histologic features and clinical outcomes of hepatocellular carcinoma.* Am J Surg Pathol, 2012. **36**(7): p. 980-6.
41. Liu, F., et al., *CD8(+) cytotoxic T cell and FOXP3(+) regulatory T cell infiltration in relation to breast cancer survival and molecular subtypes.* Breast Cancer Res Treat, 2011. **130**(2): p. 645-55.
42. Kirk, R., *Risk factors. CD8+:FOXP3+ cell ratio is a novel survival marker for colorectal cancer.* Nat Rev Clin Oncol, 2010. **7**(6): p. 299.
43. Pedoeem, A., et al., *Programmed death-1 pathway in cancer and autoimmunity.* Clin Immunol, 2014. **153**(1): p. 145-52.
44. Zhang, X., et al., *Structural and functional analysis of the costimulatory receptor programmed death-1.* Immunity, 2004. **20**(3): p. 337-47.
45. Lazar-Molnar, E., et al., *Crystal structure of the complex between programmed death-1 (PD-1) and its ligand PD-L2.* Proc Natl Acad Sci U S A, 2008. **105**(30): p. 10483-8.
46. Lin, D.Y., et al., *The PD-1/PD-L1 complex resembles the antigen-binding Fv domains of antibodies and T cell receptors.* Proc Natl Acad Sci U S A, 2008. **105**(8): p. 3011-6.
47. Cheng, X., et al., *Structure and interactions of the human programmed cell death 1 receptor.* J Biol Chem, 2013. **288**(17): p. 11771-85.
48. Sheppard, K.A., et al., *PD-1 inhibits T-cell receptor induced phosphorylation of the ZAP70/CD3zeta signalosome and downstream signaling to PKCtheta.* FEBS Lett, 2004. **574**(1-3): p. 37-41.
49. Ott, P.A., F.S. Hodi, and C. Robert, *CTLA-4 and PD-1/PD-L1 blockade: new immunotherapeutic modalities with durable clinical benefit in melanoma patients.* Clin Cancer Res, 2013. **19**(19): p. 5300-9.
50. Yao, S. and L. Chen, *PD-1 as an immune modulatory receptor.* Cancer Journal, 2014. **20**(4): p. 26.
51. Nishimura, H., et al., *Developmentally regulated expression of the PD-1 protein on the surface of double-negative (CD4−CD8−) thymocytes.* Int Immunol, 1996. **8**(5): p. 773-80.
52. Huang, X., et al., *PD-1 expression by macrophages plays a pathologic role in altering microbial clearance and the innate inflammatory response to sepsis.* Proc Natl Acad Sci U S A, 2009. **106**(15): p. 6303-8.
53. Pena-Cruz, V., et al., *PD-1 on immature and PD-1 ligands on migratory human Langerhans cells regulate antigen-presenting cell activity.* J Invest Dermatol, 2010. **130**(9): p. 2222-30.
54. Keir, M.E., et al., *PD-1 and its ligands in tolerance and immunity.* Annu Rev Immunol, 2008. **26**: p. 677-704.
55. Guan, J., et al., *Programmed death ligand-1 (PD-L1) expression in the programmed death receptor-1 (PD-1)/PD-L1 blockade: a key player against various cancers.* Arch Pathol Lab Med, 2017. **141**(6): p. 851-861.
56. Taube, J.M., et al., *Colocalization of inflammatory response with B7-h1 expression in human melanocytic lesions supports an adaptive resistance mechanism of immune escape.* Sci Transl Med, 2012. **4**(127): p. 127ra37.
57. Sanmamed, M.F. and L. Chen, *Inducible expression of B7-H1 (PD-L1) and its selective role in tumor site immune modulation.* Cancer J, 2014. **20**(4): p. 256-61.
58. Topalian, S.L., C.G. Drake, and D.M. Pardoll, *Targeting the PD-1/B7-H1(PD-L1)*

pathway to activate anti-tumor immunity. Curr Opin Immunol, 2012. **24**(2): p. 207-12.
59. Gargiulo, P., et al., *Tumor genotype and immune microenvironment in POLE-ultramutated and MSI-hypermutated Endometrial Cancers: New candidates for checkpoint blockade immunotherapy?* Cancer Treat Rev, 2016. **48**: p. 61-8.
60. Le, D.T., et al., *PD-1 Blockade in Tumors with Mismatch-Repair Deficiency.* N Engl J Med, 2015. **372**(26): p. 2509-20.
61. Le, D.T., et al., *Mismatch repair deficiency predicts response of solid tumors to PD-1 blockade.* Science, 2017. **357**(6349): p. 409-413.
62. Santin, A.D., et al., *Regression of Chemotherapy-Resistant Polymerase epsilon (POLE) Ultra-Mutated and MSH6 Hyper-Mutated Endometrial Tumors with Nivolumab.* Clin Cancer Res, 2016. **22**(23): p. 5682-5687.
63. Ott, P.A., et al., *Safety and Antitumor Activity of Pembrolizumab in Advanced Programmed Death Ligand 1-Positive Endometrial Cancer: Results From the KEYNOTE-028 Study.* J Clin Oncol, 2017. **35**(22): p. 2535-2541.
64. Makker, V., et al., *A phase Ib/II trial of lenvatinib (LEN) plus pembrolizumab (Pembro) in patients (Pts) with endometrial carcinoma.* Journal of Clinical Oncology, 2017. **35**(15_suppl): p. 5598-5598.
65. Durkacz, B.W., et al., *(ADP-ribose)n participates in DNA excision repair.* Nature, 1980. **283**(5747): p. 593-6.
66. El-Khamisy, S.F., et al., *A requirement for PARP-1 for the assembly or stability of XRCC1 nuclear foci at sites of oxidative DNA damage.* Nucleic Acids Res, 2003. **31**(19): p. 5526-33.
67. Hassa, P.O. and M.O. Hottiger, *The diverse biological roles of mammalian PARPS, a small but powerful family of poly-ADP-ribose polymerases.* Front Biosci, 2008. **13**: p. 3046-82.
68. Masson, M., et al., *XRCC1 is specifically associated with poly(ADP-ribose) polymerase and negatively regulates its activity following DNA damage.* Mol Cell Biol, 1998. **18**(6): p. 3563-71.
69. Farmer, H., et al., *Targeting the DNA repair defect in BRCA mutant cells as a therapeutic strategy.* Nature, 2005. **434**(7035): p. 917-21.
70. Patel, A.G., J.N. Sarkaria, and S.H. Kaufmann, *Nonhomologous end joining drives poly(ADP-ribose) polymerase (PARP) inhibitor lethality in homologous recombination-deficient cells.* Proc Natl Acad Sci U S A, 2011. **108**(8): p. 3406-11.
71. Turner, N., A. Tutt, and A. Ashworth, *Hallmarks of 'BRCAness' in sporadic cancers.* Nat Rev Cancer, 2004. **4**(10): p. 814-9.
72. Cancer Genome Atlas Research, N., *Integrated genomic analyses of ovarian carcinoma.* Nature, 2011. **474**(7353): p. 609-15.
73. Kaelin, W.G., Jr., *The concept of synthetic lethality in the context of anticancer therapy.* Nat Rev Cancer, 2005. **5**(9): p. 689-98.
74. Fong, P.C., et al., *Inhibition of poly(ADP-ribose) polymerase in tumors from BRCA mutation carriers.* N Engl J Med, 2009. **361**(2): p. 123-34.
75. Gelmon, K.A., et al., *Olaparib in patients with recurrent high-grade serous or poorly differentiated ovarian carcinoma or triple-negative breast cancer: a phase 2, multicentre, open-label, non-randomised study.* Lancet Oncol, 2011. **12**(9): p. 852-61.
76. Kummar, S., et al., *Advances in using PARP inhibitors to treat cancer.* BMC Med, 2012. **10**: p. 25.

77. Ledermann, J., et al., *Olaparib maintenance therapy in platinum-sensitive relapsed ovarian cancer.* N Engl J Med, 2012. **366**(15): p. 1382-92.
78. Kaufman, B., et al., *Olaparib monotherapy in patients with advanced cancer and a germline BRCA1/2 mutation.* J Clin Oncol, 2015. **33**(3): p. 244-50.
79. Xing, D. and S. Orsulic, *A mouse model for the molecular characterization of brca1-associated ovarian carcinoma.* Cancer Res, 2006. **66**(18): p. 8949-53.
80. Huang, J., et al., *The PARP1 inhibitor BMN 673 exhibits immunoregulatory effects in a BRCA1(-/-) murine model of ovarian cancer.* Biochem Biophys Res Commun, 2015. **463**(4): p. 551-6.
81. Meade, M., *Efficacy evaluation of niraparib and TSR-042 alone and in combination against subcutaneous BRKras-luc murine ovarian carcinoma in female FVB mice*. 2016, Molecular Imaging.
82. Mills, G., *Efficacy of niraparib as a single agent and in combination with PD-1 antibody in LPA1-T22 tumor model*. 2017, TESARO, Inc.
83. *Efficacy and PD study in KLN205 subcutaneous lung squamous cell cancer model*. 2016, Pharmaron.
84. Romero I, R.M., Medina M, Serrano R, Guerra EM, Cortes Salgado A, Pérez B, Minig L, Casado A, Coronado P, Cueva JF, Vilar A, Torné A, Ordi J, Cros S, Salinas G, Santacana M, Schoenenberger-Arnaiz JA, Llombart-Cussac A, Poveda A, *Preoperative olaparib in early-stage endometrial cancer (EC): A phase 0, window of opportunity trial to evaluate the PARP inhibition effect, targeting cell cycle-related proteins (POLEN study).* J Clin Oncol, 2018. **36**((suppl; abstr 5598)).
85. Shen, J., et al., *ARID1A deficiency promotes mutability and potentiates therapeutic antitumor immunity unleashed by immune checkpoint blockade.* Nat Med, 2018. **24**(5): p. 556-562.
86. Dedes, K.J., et al., *PTEN deficiency in endometrioid endometrial adenocarcinomas predicts sensitivity to PARP inhibitors.* Sci Transl Med, 2010. **2**(53): p. 53ra75.
87. Mendes-Pereira, A.M., et al., *Synthetic lethal targeting of PTEN mutant cells with PARP inhibitors.* EMBO Mol Med, 2009. **1**(6-7): p. 315-22.
88. Brown, J.S., R. Sundar, and J. Lopez, *Combining DNA damaging therapeutics with immunotherapy: more haste, less speed.* Br J Cancer, 2018. **118**(3): p. 312-324.
89. Shen, W.H., et al., *Essential role for nuclear PTEN in maintaining chromosomal integrity.* Cell, 2007. **128**(1): p. 157-70.
90. Li, P., et al., *P90 RSK arranges Chk1 in the nucleus for monitoring of genomic integrity during cell proliferation.* Mol Biol Cell, 2012. **23**(8): p. 1582-92.
91. Miyasaka, A., et al., *Anti-tumor activity of olaparib, a poly (ADP-ribose) polymerase (PARP) inhibitor, in cultured endometrial carcinoma cells.* BMC Cancer, 2014. **14**: p. 179.
92. Stracker, T.H. and J.H. Petrini, *The MRE11 complex: starting from the ends.* Nat Rev Mol Cell Biol, 2011. **12**(2): p. 90-103.
93. Koppensteiner, R., et al., *Effect of MRE11 loss on PARP-inhibitor sensitivity in endometrial cancer in vitro.* PLoS One, 2014. **9**(6): p. e100041.
94. Mackay, H.J., et al., *Molecular determinants of outcome with mammalian target of rapamycin inhibition in endometrial cancer.* Cancer, 2014. **120**(4): p. 603-10.
95. Mutter, G.L., et al., *Altered PTEN expression as a diagnostic marker for the earliest endometrial precancers.* J Natl Cancer Inst, 2000. **92**(11): p. 924-30.

96. Djordjevic, B., et al., *Clinical assessment of PTEN loss in endometrial carcinoma: immunohistochemistry outperforms gene sequencing.* Mod Pathol, 2012. **25**(5): p. 699-708.
97. Risinger, J.I., et al., *PTEN/MMAC1 mutations in endometrial cancers.* Cancer Res, 1997. **57**(21): p. 4736-8.
98. Tashiro, H., et al., *Mutations in PTEN are frequent in endometrial carcinoma but rare in other common gynecological malignancies.* Cancer Res, 1997. **57**(18): p. 3935-40.
99. Pallares, J., et al., *Immunohistochemical analysis of PTEN in endometrial carcinoma: a tissue microarray study with a comparison of four commercial antibodies in correlation with molecular abnormalities.* Mod Pathol, 2005. **18**(5): p. 719-27.
100. Garg, K., et al., *Pathologic scoring of PTEN immunohistochemistry in endometrial carcinoma is highly reproducible.* Int J Gynecol Pathol, 2012. **31**(1): p. 48-56.
101. Mohindra, A., et al., *Defects in homologous recombination repair in mismatch-repair-deficient tumour cell lines.* Hum Mol Genet, 2002. **11**(18): p. 2189-200.
102. Slichter, S.J., *Evidence-based platelet transfusion guidelines.* Hematology Am Soc Hematol Educ Program, 2007: p. 172-8.
103. Julie R. Brahmer, et al., *Management of Immune-Related Adverse Events in Patients Treated With Immune Checkpoint Inhibitor Therapy: American Society of Clinical Oncology Clinical Practice Guideline.* Journal of Clinical Oncology 36, no. 17 (June 10, 2018) 1714-1768
104. Smith, T.J., et al., *2006 update of recommendations for the use of white blood cell growth factors: an evidence-based clinical practice guideline.* J Clin Oncol, 2006. **24**(19): p. 3187-205.
105. Yanagawa, N., et al., *Loss of phosphatase and tensin homolog protein expression is an independent poor prognostic marker in lung adenocarcinoma.* J Thorac Oncol, 2012. **7**(10): p. 1513-21.
106. Eisenhauer, E.A., et al., *New response evaluation criteria in solid tumours: revised RECIST guideline (version 1.1).* Eur J Cancer, 2009. **45**(2): p. 228-47.

APPENDIX 1 PERFORMANCE STATUS CRITERIA

ECOG Performance Status Scale	
Grade	Descriptions
0	Normal activity. Fully active, able to carry on all pre-disease performance without restriction.
1	Symptoms, but ambulatory. Restricted in physically strenuous activity, but ambulatory and able to carry out work of a light or sedentary nature (*e.g.*, light housework, office work).
2	In bed <50% of the time. Ambulatory and capable of all self-care, but unable to carry out any work activities. Up and about more than 50% of waking hours.
3	In bed >50% of the time. Capable of only limited self-care, confined to bed or chair more than 50% of waking hours.
4	100% bedridden. Completely disabled. Cannot carry on any self-care. Totally confined to bed or chair.
5	Dead.

APPENDIX 2 DATA MANAGEMENT GUIDELINES

Case Report Form Submission Schedule

The Eligibility Checklist will be a paper CRF that will be provided by the Drug Development Central Office and all other data required for the study will be collected in eCRFs in Medidata. The form submission schedule is outlined below.

Case Report Form	Submission Schedule
Eligibility Checklist	At the time of registration
Baseline Form	Within 3 weeks of on study date
On Treatment Form	Within 3 weeks of the end of each cycle of treatment
Off Treatment Form	Within 3 weeks of the patient coming off-study
Short Follow-up Form	Within 3 weeks of the patient coming to clinic.
Final Report Form	Within 3 weeks of the patient's death being known to the investigator unless this constitutes a reportable adverse event when it should be reported according to expedited guidelines (see Section 7.3)

Monitoring

This is an investigator initiated study and study monitoring will be performed by the Drug Development Program Central Office or its designate.

Data in the Medidata Rave eCRFs will be monitored on a regular basis and quality assurance measures will be performed. Electronic data queries as well as paper query letters may be issued to the site.

The Drug Development Program Central Office Coordinator is responsible for remotely reviewing eCRFs as the Data Manager according to a study specific Data Management Plan. The Data manager is to complete their review within 3 weeks of data entry. The Central Office Coordinator will collect the following source documentation:

- Copies of all signed informed consent forms (ICFs): initial, optional, re-consents, etc.
- Documentation of the consent process and the re-consent process (verbal and written) as appropriate
- Response / Tumor Assessment Worksheets
- Radiology Reports
- Pathology Reports
- PTEN Analysis Reports

A third-party designate will be contracted as the Clinical Research Associate to perform onsite monitoring and source data verification according to the terms outlined in the Monitoring Plan.

Regulatory Requirements

- Please submit all required documents to the Drug Development Program Central Office.
- Canadian Principal Investigators must submit a completed Qualified Investigator Undertaking.
- All investigators must have an up-to-date CV (signed within 2 years) on file with the Drug Development Program Central Office.
- Laboratory certification/accreditation and normal ranges are required
- Confirmation of all investigators having undergone training in the Protection of Human Research Subjects is required. It is preferred that other staff involved in the trial also undergoes such training.
- Investigators and site staff are required to complete Medidata eCRF training modules depending on delegated tasks
- Consent forms must be reviewed by the Central Office before submission to the local ethics regulatory board (REB/IRB) and must include a statement that 1) information will be sent to and 2) medical records will be reviewed by the Drug Development Program Central Office.
- A Membership list of the local ethics board is required.
- A copy of the initial approval letter from the ethics board must be submitted to the Drug Development Program Central Office.
- A completed Site Participant List/Training Log is required and must be submitted to Drug Development Program Central Office.
- Continuing approval will be obtained at least yearly until follow-up on patients is completed and no further data is being obtained for research purposes.

APPENDIX 3 WHO MDS CLASSIFICATION CRITERIA

The 2008 World Health Organization (WHO) classification of myelodysplastic syndromes (MDS), as well as the International Prognostic Scoring System (IPSS), are provided below.

Myelodysplastic syndromes are a group of clonal myeloid neoplasms characterized by ineffective hematopoiesis that present clinically as cytopenia(s), dysplasia in one or more hematopoietic cell lines in the bone marrow, and risk of transformation to acute myeloid leukemia (AML)

The WHO classification system of MDS relies on incorporating clinical features, peripheral blood and bone marrow findings, and cytogenetic analysis. This classification also includes a collection of heterogeneous neoplasms that share features of MDS and myeloproliferative neoplasms.

Refractory cytopenia with uniineage dysplasia (RCUD):

Blood: single cytopenia or bicytopenia

Bone marrow: dysplasia in ≥ 10% of 1 cell line, < 5% blasts

Refractory anemia with ring sideroblasts (RARS):

Blood: anemia, no blasts

Bone marrow: ≥ 15% of erythroid precursors with ring sideroblasts, erythroid dysplasia only, < 5% blasts

Refractory cytopenia with multilineage dysplasia (RCMD):

Blood: cytopenia(s), $< 1 \times 10^{9}$/L monocytes

Bone marrow: dysplasia in ≥ 10% of cells in ≥ 2 hematopoietic lineages, ±15% ring sideroblasts, < 5% blasts

Refractory anemia with excess blasts-1 (RAEB-1):

Blood: cytopenia(s) ≥ 2-4% blasts, $< 1 \times 10^{9}$/L monocytes

Bone marrow: unilineage or multilineage dysplasia, no Auer rods, 5-9% blasts

Refractory anemia with excess blasts-2 (RAEB-2):

Blood: cytopenia(s), 5-19% blasts, $< 1 \times 10^{9}$/L monocytes

Bone marrow: unilineage or multilineage dysplasia, Auer rods, ± 10-19% blasts

Myelodysplastic syndrome – unclassified (MDS-U):

Blood: cytopenias

Bone marrow: unilineage dysplasia or no dysplasia but characteristic MDS cytogenetics, < 5% blasts

MDS associated with isolated del(5q):

Blood: anemia, platelet levels normal or increased

Bone marrow: unilineage erythroid dysplasia, isolated del(5q), < 5% blasts

Therapy-related MDS (t-MDS):

Blood and bone marrow findings of 1 of the above diagnostic categories (frequently with multilineage dysplasia)

Previous history of exposure to cytotoxic chemotherapy and/or radiation therapy administered for treatment of cancer or nonneoplastic disease

2008 WHO terminology: therapy-related myeloid neoplasm
